# Supplementary material for: The bound growth of induced earthquakes could de-risk hydraulic fracturing
Source: Commun Earth Environ. 2025 Nov 22;6(1):995. doi: 10.1038/s43247-025-02881-2 (PMC12685737; doi:10.1038/s43247-025-02881-2)
Supplement: Supplementary file 2 — Supplementary Information [file 43247_2025_2881_MOESM2_ESM.pdf]

Supplementary Information for the article

"The bound growth of induced earthquakes could de-risk hydraulic fracturing"

by

Ryan Schultz, Federica Lanza, Ben Dyer, Dimitrios Karvounis, Rémi Fiori,

Peidong Shi, Vanille Ritz, Linus Villiger, Peter Meier, & Stefan Wiemer

published in

communications earth & environment

Contents of this file

Supplementary Text S1-S8
Supplementary Figures S1-S42
Supplementary Tables S1-S2

Supplementary Text

Text S1: A general definition of terms

Here, we provide a brief definition of relevant terms. The variable M is the magnitude of an earthquake while M_{LRG} refers to the largest event observed from a sample catalogue of size *N*. If a catalogue is unbound, the relationship for the modal estimate of the expected largest event is $M_{LRG} = log_{10}(N)/b + M_C$ [van der Elst et al., 2016], which depends on the *b*-value and the magnitude-of-completeness (Mc). Sequential differences in magnitudes are denoted as ΔM. This allows for composite terms to be constructed, such as ΔM_{LRG} – the 'jumps' in magnitude of the largest observed events. All the terms introduced in this paragraph are empirically observable quantities, given a sample catalogue. Note that for a given sample, ΔM_{LRG} will have fewer elements than the parent catalogue of magnitudes M.

In this study, M_{MAX} is defined as the maximum possible magnitude. This means that M_{MAX} is not directly observable from a catalogue. Theoretical models may make hypotheses as to M_{MAX}, based on various geophysical principles. This paper will focus on two M_{MAX} hypotheses that depend on the total injected volume V: either being McGarr-like (*i.e.*, seismic moment is proportional to V^1) [McGarr, 2014; Hallo et al., 2014], or based on Galis-like (*i.e.*, seismic moment is proportional to $V^{3/2}$) [Galis et al., 2017]. A visual depiction of this terminology is given in Figure S1.

Text S2: Simple indicators of M_{MAX}

Here, we briefly define some simple tests to provide an initial assessment of M_{MAX}. This is accomplished through an examination of the Gutenberg-Richter magnitude frequency distribution (GR-MFD) [Gutenberg & Richter, 1944] and discrepancies between observed-expected largest events. This section constitutes the first step in our overall workflow (Figure 2).

2.1 Fitting the Gutenberg-Richter magnitude frequency distribution (GR-MFD)

The GR-MFD is a relationship that describes the frequency and amount of earthquake magnitudes: $N = 10^{a}10^{-b\mathrm{M}}$. The *a*-value describes the scaling to the total amount of earthquakes *N*, while the *b*-value is the proportionality of big-to-small events. Given a catalogue of earthquake magnitudes M, this relationship can be fit through Maximum Likelihood Estimators [Marzocchi & Sandri, 2003]. For real datasets, a lower bound truncation called the magnitude-of-completeness (Mc) is introduced to account for incompleteness of event detection. Many methods exist to evaluate Mc robustly, to account for the incompleteness in the detection of low magnitude events, thusly to avoid biases during the GR-MFD fitting process. In this study, Mc is selected by searching for the value that maximizes the goodness-of-fit metrics like R^2 [Schultz et al., 2018] or minimizes the negative log-likelihood of the GR-MFD. In cases with a goodness-of-fit plateaus/valleys, we select the value of Mc closest to plateau start. Sometimes we are conservative in our Mc choice by selecting a value slightly larger (never more than +0.1) than optimal. Note that this conservativism will have a detrimental effect on finding bound cases. Furthermore, in later sections, we will perform a perturbation analysis on the Mc choice. The Mc value selected by this process is typically near (but skewed right-ward of) the peak bin of the non-cumulative GR-MFD. In this study, we are fortunate to have many large and well-behaved datasets.

As part of the simple indicators of M_{MAX}, a visual comparison of the GR-MFD fit against the observed data is used to qualitatively examine for the presence of M_{MAX}. Catalogues that are relatively deficient in large magnitudes (compared to their fits) are possible candidates for an M_{MAX}. We note that this deficiency in large magnitude events is a key metric for identifying if/when M_{MAX} can be constrained [Holschneider et al., 2011; Schultz, 2024].

2.2 Examining discrepancies between the observed and expected largest event magnitudes

The expected value of the largest event magnitude (M_{LRG}) can be quantified by assuming a GR-MFD and then using order statistics to determine the modal value [van der Elst et al., 2016]. The formulation is as follows: $\mathrm{M_{LRG}} = \mathrm{Mc} + log_{10}(N)/b$. Where N is the total number of events larger than Mc. To provide some small quantification for the presence of an M_{MAX}, we examine the degree-of-truncation (δM_{LRG}), which is discrepancy between the observed largest magnitude event and the expected largest event magnitude. We can use the prior GR-MFD fits to determine the expected value of M_{LRG} and then compare that against the observed value to determine δM_{LRG}. We also use the inverted cumulative distribution function [van der Elst et al., 2016] to determine the percentile of the δM_{LRG} discrepancy – or the likelihood of this discrepancy occurring, assuming an unbound catalogue.

As part of the simple indicators of M_{MAX}, catalogues that exhibit negative observed-expected discrepancies (δM_{LRG}) potentially suggest the presence of an M_{MAX}. We note that δM_{LRG} discrepancies are an important proxy metric for the resolvability of CAP-tests. Usually, M_{LRG}-M_{MAX} differences of -0.5 M or less are required to confidently assert the presence of an M_{MAX}. Note that this M_{LRG}-M_{MAX} difference is the expected value where quantitative inferences of M_{MAX} can start being made with 95% confidence, from theoretical considerations [Equation 15; Holschneider et al., 2011].

Text S3: Definition of CAP-tests

The CAP-tests are a novel suite of statistical methods aimed at discerning the influence of M_{MAX} on a catalogue; each test is rooted in fundamentally different statistical frameworks, to ensure the robust cross-validation of results. In this sense, when all the CAP/simple-tests suggest a similar bound/unbound result, we can be (more) confident that we have reached the right interpretation – even if there might be data/method issues

In this section, we explicitly define each of the CAP-tests. We also refer readers to a past study that defines the CAP-tests in detail, provides comprehensive sensitivity tests, and highlights real-data applications [Schultz, 2024]. These tests build upon the simple pre-screening assessments (Text S2), logically answering a sequence of questions to discern the influence of M_{MAX} more rigorously (Figure 2).

3.1 The Kolmogorov-Smirnov test (KS-test)

The first test is rooted in the statistical framework of hypothesis testing. Here, we take advantage of the fact that the distribution of magnitudes (M) and the distribution of jumps in the sequence of large events (ΔM_{LRG}) is the same if unbound, but differ when there is an M_{MAX} upper bound [Schultz, 2024]. This fact ideally lends itself to hypothesis testing via the KS-test [Berger & Zhou, 2014]. Given a catalogue, both M and ΔM_{LRG} can be observed (Figure S1). We can then compare these two observations against each other to test if they are drawn from the same distribution (or not), via the KS-test. This approach is advantageous in that it is non-parametric – *i.e.*, it is completely data-driven and imposes no assumptions about the kind of distributions M or ΔM_{LRG} were drawn from. Because of this, we do not need to fit the data to a GR-MFD or have any knowledge/estimates of the *b*-value to perform our KS-test. Confidence in the KS-test is reported as compliments of standard *p*-values, where 95% is a common threshold used to declare statistical significance.

Since this test is only interested in discerning the existence of an M_{MAX}, additional catalogue realizations can be drawn through reshuffling the order of events. In this sense, bootstrapping can be employed to generate numerous catalogue realizations in which the KS-test is repeated. This provides more robust *p*-value estimates. Testing on both synthetic and real datasets suggests that the KS-test is significantly more sensitive to discerning M_{MAX} than approaches that attempt to appraise GR-MFD fits [Schultz, 2024]. Testing on synthetic catalogues

also demonstrates that this reshuffling process does not overinflate false-positives [Schultz, 2024]. That said, to be able to discern the influence of M_{MAX}, M_{LRG}-M_{MAX} differences of -0.5 M or better are usually required. Note that this M_{LRG}-M_{MAX} difference is the expected value where quantitative inferences of M_{MAX} can start being made with 95% confidence, from theoretical considerations [Equation 15; Holschneider et al., 2011]. As well, synthetic testing on unbound cases shows that this KS-test produces false-positives at the rate expected for a *p*-value distribution [Schultz, 2024].

While this formulation of the KS-test is powerful, we also provide a word of caution towards a potential interpretation pitfall: this particular method is testing for differences between the distributions M and ΔM_{LRG}. The presence of an M_{MAX} is one possible reason for this difference, but others may also confound a clear interpretation (*e.g.*, temporal changes in *b*-value, kinked distributions). Thus, the KS-test should be suitably pre-processed or complemented with other tests to increase the certainty of an M_{MAX} interpretation. This is part of the rationale for including both the simple tests (Text S2) and complementary CAP-tests – to help cover for deficiencies like this (Figure 2).

3.2 The Maximum Likelihood Estimator (MLE-test)

The next test is rooted in the statistical framework of Maximum Likelihood Estimation. If there is some suggestive evidence for the existence of an M_{MAX}, the next natural step is to quantify this value. The log-likelihood function is defined as follows:

$$\ln\big(\mathcal{L}(\mathrm{M};\boldsymbol{\theta})\big) = \sum_{i} \ln\big(\, f_M(\mathrm{M}; b, \mathrm{Mc}, \mathrm{M}_{\mathrm{MAX}})\,\big) - \sum_{j} \ln\big(\, f_M(\Delta\mathrm{M}_{\mathrm{LRG}}; b, 0, \mathrm{M}_{\mathrm{MAX}} - \mathrm{M}_{\mathrm{LRG}})\,\big)$$

Where the probability density function of the GR-MFD is given by $f_M(\mathrm{M})$, with a set of model parameters $\boldsymbol{\theta}$ [Schultz, 2024]. This function essentially entails two parts: the 'standard' log-likelihood for the catalogue magnitudes M (first term, right-hand side of equation) and the log-likelihood of the jumps in largest events ΔM_{LRG} (second term, right-hand side of equation). The optimal set of model parameters $\boldsymbol{\theta}$ are then solved for via numerical methods to maximize the log-likelihood, given the observed catalogue data M and ΔM_{LRG}. This is performed in two steps. First, using standard approaches to constrain the *b*-value [Marzocchi & Sandri, 2003], with the optimal Mc estimate (Text S2.1). Second, using the above log-likelihood to constrain M_{MAX}. In this study,

we consider the simplest M_{MAX} truncation variant for f_M(M) [Schultz, 2024]; in later sections (Section 4.4 & Figure 9) we considered the impact of tapered GR-MFDs [Kagan, 2002].

If this test is only interested in discerning a stationary value of M_{MAX}, additional catalogue realizations can be drawn through reshuffling the order of events. Similar to the KS-test, bootstrapping can be employed to generate numerous catalogue realizations in which the MLE-test is repeated. This provides more robust M_{MAX} estimates. Testing on both synthetic and real datasets suggests that the MLE-test is sensitive to quantifying M_{MAX} within a hundredth of a magnitude unit (or better) when M_{MAX}-M_{LRG} discrepancies are better than -0.5 M. Note that this M_{LRG}-M_{MAX} difference is the expected value where quantitative inferences of M_{MAX} can start being made with 95% confidence, from theoretical considerations [Equation 15; Holschneider et al., 2011].

In cases where the MLE-test is applied to unbound catalogues, bootstrapped estimates of M_{MAX} will be much larger than M_{LRG} and standard deviations can be on the order of 1 magnitude unit.

3.3 The Ensemble Weighting test (EW-test)

The third and final test is rooted in the statistical framework of likelihood inference. Together, the two prior tests provide suitable evidence for the existence and single-value of M_{MAX}. However, M_{MAX} may be a function of time or injected volume, becoming some non-stationary value throughout the catalogue duration. Certainly, M_{MAX} processes relevant for induced seismicity and hydraulic fracturing have been proposed in the past [McGarr, 2014; Hallo et al., 2014; Galis et al., 2017]. In this sense, having an approach that can distinguish the best proposed M_{MAX} model (given the data) would be insightful.

The EW-test starts by considering an ensemble of proposed M_{MAX} models to explain the catalogue data [Schultz, 2024]. Using the previously defined log-likelihood function, both Akaike Information Criterion (AIC) and Bayesian Information Criterion (BIC) can be defined for each M_{MAX} model [Schwarz, 1978; Akaike, 1998]; note that in this study, we have used the small sample size corrections for both AIC/BIC scores [Sugiura, 1978; McQuarrie, 1999]. Next, we compute the differences in AIC/BIC scores, by subtracting the score of the best M_{MAX} model. These score differences can be translated into relative model weights by an exponential function [Wagenmakers & Farrell, 2004]. We combine AIC/BIC weights into a single weight by taking an

average between the two. Each M_{MAX} model will have different numbers of model parameters *K*, for use in computing AIC/BIC scores. The unbound model is the simplest with *K*=3 (m_1, *b*-value, GR-MFD variance), while bound models [McGarr, 2014; Hallo et al., 2014; Galis et al., 2017] are slightly more complex with *K*=4.

The interpretation of the M_{MAX} model weights is straightforward: larger model weights indicate a better explanation of the data. The model with the largest weight is the best explanation of the data (within the ensemble). To quantify the statistical significance of weight differences between two models, the relative odds ratio can be computed as the ratio of the two model weights (larger/smaller). Ratios of 1+ are insignificant, 3+ are substantial/positive, 10+ are strong, and 100+ is decisive [Kass & Raftery, 1995]. In this study we solely focus on odds ratios of bound models relative to the unbound null hypothesis. We note that the best model in an ensemble does not necessarily imply the veracity of the model; there could be another (unknown) model that explains the data better than all of those yet considered.

In synthetic testing, the EW-test can accurately and confidently discern the true M_{MAX} model [Schultz, 2024]. Usually, only a handful of ΔM_{LRG} observations are required to confidently identifying the true model (*i.e.*, with odds ratios of 3-10 or better). However, like the prior CAP-tests, M_{MAX} must be influencing the catalogue for meaningful inferences to be made. Said another way, if the M_{MAX} is significantly larger than M_{LRG}, then the EW-test will not be able to distinguish this particular bound case. Because of this, we feel justified in labelling the unbound hypothesis as the null hypothesis from an inference perspective. Correspondingly, from a risk management perspective, it would also be prudent to handle all cases assuming an unbound distribution – and then only react differently if there is serious evidence for a bound distribution.

Text S4: Demonstration of CAP-tests via synthetic tests

Within this supplementary section, we synthetically demonstrate the efficacy of CAP-tests for discerning bound/unbound cases. We also use this section to pedagogically demonstrate the interpretation of CAP-test results. Thus, this subsection will only consider catalogues randomly drawn via inverse transform sampling [Zhuang et al., 2012] – to better understand CAP-tests in a controlled setting first.

4.1 Building an intuition for the composite log-likelihood function

First, we show how the subtraction of the ΔM_{LRG} term from the 'standard' log-likelihood function (Text S3.2) helps in regularizing for M_{MAX} fitting/inferences. This starts by drawing catalogues of 10^3 events with a *b*-value of 1.0 and Mc of 0.0. Two catalogues are considered: one bound at an M_{MAX} of 2.0 and the other is unbound. We then compute the negative log-likelihood for a range of possible M_{MAX} estimates, normalized against the negative log-likelihood for an unbound case. This normalization means that a (normalized) negative log-likelihood score of zero is equivalent to an unbound M_{MAX} score; thus, (normalized) negative log-likelihood scores less than zero explain the data better than an unbound hypothesis.

When considering the bound case (Figure S2; top panel), the addition of the ΔM_{LRG} term improves upon the 'standard' term. MLE fitting with only the standard term will always solve for the trivial solution of the current M_{LRG}, since this will always produce the best/minimum negative log-likelihood scores. On the other hand, the addition of the ΔM_{LRG} term provides a penalty to the negative log-likelihood – creating a minimum near the true M_{MAX} value. The addition of the ΔM_{LRG} term makes an unbiased estimator of M_{MAX} during Maximum Likelihood Estimation [Schultz, 2024].

When considering the unbound case (Figure S2; bottom panel), the addition of the ΔM_{LRG} term also improves upon discernment. For example, just the 'standard' term still suggests that M_{LRG} is the best estimate of M_{MAX}. Thus, discerning between bound/unbound cases would need to rely solely on model complexity penalties (*e.g.*, AIC/BIC). By including the ΔM_{LRG} term, we now correctly recognize M_{MAX} as infinite, just from the catalogue data.

Overall, the ΔM_{LRG} term helps to regularize the M_{MAX} fitting process by providing an overfitting penalty that is absent from the 'standard' form. Based on this, we feel justified in the use of the ΔM_{LRG} term. It is important to point out that the inclusion of this penalty guarantees

that our EW-test estimates are always less confident than those considering only the 'standard' form. Thus, any bound cases we identify via EW-tests would be even more strongly stated using standard approaches that ignore the ΔM_{LRG} term.

4.2 Demonstration of CAP-tests

Here, we consider simulated scenarios to demonstrate the efficacy of CAP-tests. Again, catalogues of 10^3 events with a *b*-value of 1.0 and Mc of 0.0 are drawn. Four catalogue scenarios are considered: three which are bound by McGarr-like, Galis-like, or tectonic conditions and the last being unbound. Given one of these catalogues, we then perform our entire suite of simple-tests and CAP-tests to the synthetic data. This includes perturbed bootstrapping, initial fitting of *b*-values, and estimation of M_{MAX} models. Full (pre-)processing details of the simple-tests and CAP-tests are given in the next supplementary section (Text S5). We repeat these tests for each of the four true M_{MAX} model scenarios. Note that each scenario will impose an M_{MAX} truncation similar in degree to those inferred from the real-data results.

The first scenario we consider is the unbound catalogue (Figure S3). The CAP-tests correctly identify this scenario as unbound, in all the subset tests. For example, KS-tests confidences are much lower than 95%, the MLE-test produces large values of M_{MAX} standard error, and the EW-test identifies the unbound model as the winner. The declaration of the unbound model being the winner is supported by the unbound model having the dominant weight from N=10^1 and onwards. Initially, much of this weighting skew towards the unbound model comes from complexity penalties in the AIC/BIC formulations (since unbound models are simpler). However, penalties from the ΔM_{LRG} term in the log-likelihood are also observable as sudden shifts towards unbound-favoured weights when new M_{LRG} observations are made close to their expected values. The reason for this phenomenon was described previously (Text S4.1).

The second scenario we consider is the tectonically bound catalogue (Figure S4). This scenario considers a constant value of 2.3 as the M_{MAX}; for comparison, we would expect M_{LRG} to be 3.0 (on average), for an unbound catalogue. The CAP-tests correctly identify this scenario as bound, in all the subset tests. For example, KS-tests confidences are typically much higher than 95%, the MLE-test produces M_{MAX} values close to the true value with small standard errors, and the EW-test identifies the tectonic model as the winner. It is noteworthy to point out that the EW-test initially considers the sequence as unbound. After about 10 events, this is predominantly the

result of the resolvability of CAP-tests – only after M_{LRG} is within ~0.5 magnitude units of M_{MAX} can serious inferences about M_{MAX} be made. For example, only after the ~500th event, when the observed M_{LRG} events start becoming deficient does the tectonic model become favoured.

The third scenario we consider is the McGarr-like bound catalogue (Figure S5). This scenario considers an M_{MAX} proportional to injected volume, which ranges from 0.0 to 2.0 M over the sequence of events. The CAP-tests correctly identify this scenario as bound, in all the subset tests. For example, KS-tests confidences are significantly higher than 95%, the MLE-test has small standard errors, and the EW-test identifies the McGarr-like model as the winner. While the M_{LRG} sequence is always deficient, enough evidence needs to be collected before complexity penalties from AIC/BIC are overcome – only then (*i.e.*, by the ~25th event) does the McGarr-like model become favoured.

The fourth and final scenario we consider is applying CAP-tests to the Galis-like bound catalogue (Figure S6). This scenario considers an M_{MAX} proportional to injected volume, which ranges from -0.5 to 2.5 M over the sequence of events. These tests correctly identify this scenario as bound, in all the subset tests. For example, KS-tests confidences are typically higher than 95%, the MLE-test has small standard errors, and the EW-test identifies the Galis-like model as the winner. The sequence of EW-test weights follows a similar pattern to the McGarr-like results. However, we note that Galis-like models are generally more difficult to discern, since their M_{MAX} can progress similarly to an unbound M_{LRG} sequence.

Additional methodological testing results are available in prior studies [Schultz, 2024]. We have shared these codes online at GitHub, to help interested readers get an intuition for the CAP-test process and replicate/build-upon our results.

Text S5: Expanded details of study results

Within this supplementary section we expand upon the details of our study results. The subsections are first divided into the cases that we have focused on (Figure S7): PNR1-z, FORGE, PNR-2, and Soultz-sous-Forêts. Within these sub-sections we further stratify into a justification of our clustering choices, results from the simple tests, and then results from the CAP-tests.

5.1 Results from PNR-1z

5.1.1 Clustering justifications for PNR-1z

The judicious handling of earthquake clustering is important for correctly interpreting the bound/unbound growth of magnitudes in a sequence. Here, we provide some justifications for our clustering choices. Prior results [Clarke et al., 2019] have suggested that all stages at PNR-1z are hydraulically linked together (Figure S8). Thus, we have considered the entire operation at PNR-1z as a single hydraulically connected cluster.

5.1.2 Simple M_{MAX} indicators at PNR-1z

To assess the potential for bound/unbound earthquake growth, we first employ the simple GR-MFD analyses. Overall, the entire operation appears to be visually deficient in large magnitude events (Figures S9). The fit to the GR-MFD is based on the catalogues available online. To account for possible errors in magnitudes, the GR-MFD fitting follows a 50-trial bootstrap process, in which the catalogue magnitudes are dithered by ±0.1 (this includes a dithered truncation magnitude). The Mc is -0.70; shifting the Mc by ±0.2 does not meaningfully change the results here (or in the following subsections). The fits to the dataset are well-constrained with a *b*-value of 1.25±0.04, as reported from the mean and standard deviation of the bootstrapped *b*-value distribution. Correspondingly, the δM_{LRG} discrepancy is -0.47 M_W. Assuming an unbound catalogue, this δM_{LRG} discrepancy would be less than a 2nd percentile event. These initial assessments are suggestive of some M_{MAX} upper bound restricting the PNR-1z catalogue growth.

Further CAP-tests will be needed, to provide robust and reliable estimates of M_{MAX} beyond these initial suggestions.

5.1.3 CAP-test results for PNR-1z

Next, we use the CAP-tests to detect and assess the potential for M_{MAX} more rigorously. The KS-test is performed 50 times, in which the catalogue magnitudes are dithered (including a dithered truncation magnitude). The KS-test also performs 100 reshuffles within each trial. The geometric means of KS-test *p*-value compliments are 99.82%. Similarly, we do 50 MLE-tests using dithered catalogues and 100 reshuffles within each trial. The fitted M_{MAX} has a standard deviation <0.01 M_W. Together, KS-test and MLE-test results show strong evidence for an M_{MAX}.

Next, we perform the EW-tests on the PNR-1z data. Earthquake magnitudes are truncated based on the Mc. We then propose three M_{MAX} models to explain the data: linearly proportional to volume [McGarr, 2014], proportional to volume with a 3/2 exponent [Galis et al., 2017], and unbound as the null hypothesis. Bound M_{MAX} models are fit to the data, such that the smallest difference between predicted M_{MAX} and observed M_{LRG} is 0.1 M_W. The results of the EW-tests are shown in plots (Figures S10). The Galis-like model is >100 times more likely than the unbound model. This is another strong indication for the bound models explaining the data better than the unbound model.

Together, these results are strong evidence for a physical process bounding the M_{MAX} at PNR-1z. The KS-test and MLE-test provide strong evidence for the existence of an M_{MAX}. This is also corroborated by the simple analysis (Text S5.1.2; Figure S9). We have summarized these results in Table S1.

5.2 Results from Helsinki St1

5.2.1 Clustering justifications for Helsinki St1

The judicious handling of earthquake clustering is important for correctly interpreting the bound/unbound growth of magnitudes in a sequence. Here, we provide some justifications for our clustering choices. Prior results [Kwiatek et al., 2019] have suggested that all stages at St1 are hydraulically linked together (Figure S11). Thus, we have considered the entire operation at St1 as a single hydraulically connected cluster.

5.2.2 Simple M_{MAX} indicators at Helsinki St1

To assess the potential for bound/unbound earthquake growth, we first employ the simple GR-MFD analyses. Overall, the entire operation appears to be visually deficient in large magnitude events (Figures S12). The fit to the GR-MFD is based on the catalogues available

online. To account for possible errors in magnitudes, the GR-MFD fitting follows a 50-trial bootstrap process, in which the catalogue magnitudes are dithered by ±0.1 (this includes a dithered truncation magnitude). The Mc is 0.40; shifting the Mc by ±0.2 does not meaningfully change the results here (or in the following subsections). The fits to the dataset are well-constrained with a *b*-value of 1.31±0.06, as reported from the mean and standard deviation of the bootstrapped *b*-value distribution. Correspondingly, the δM_{LRG} discrepancy is -0.69 M_W. Assuming an unbound catalogue, this δM_{LRG} discrepancy would be less than a 1st percentile event. These initial assessments are suggestive of some M_{MAX} upper bound restricting the Helsinki St1 catalogue growth.

Further CAP-tests will be needed, to provide robust and reliable estimates of M_{MAX} beyond these initial suggestions.

5.2.3 CAP-test results for Helsinki St1

Next, we use the CAP-tests to detect and assess the potential for M_{MAX} more rigorously. The KS-test is performed 50 times, in which the catalogue magnitudes are dithered (including a dithered truncation magnitude). The KS-test also performs 100 reshuffles within each trial. The geometric mean of the KS-test *p*-value compliments is 98.17%. Similarly, we do 50 MLE-tests using dithered catalogues and 100 reshuffles within each trial. The fitted M_{MAX} has a standard deviation 0.03 M_W. Together, KS-test and MLE-test results show strong evidence for an M_{MAX}.

Next, we perform the EW-tests on the St1 data. Earthquake magnitudes are truncated based on the Mc. We then propose three M_{MAX} models to explain the data: linearly proportional to volume [McGarr, 2014], proportional to volume with a 3/2 exponent [Galis et al., 2017], and unbound as the null hypothesis. Bound M_{MAX} models are fit to the data, such that the smallest difference between predicted M_{MAX} and observed M_{LRG} is 0.1 M_W. The results of the EW-tests are shown in plots (Figures S13). The McGarr-like model is >100 times more likely than the unbound model. The Galis-like model is ~57 times more likely than the unbound model. This is another strong indication for the bound models explaining the data better than the unbound model.

Together, these results are strong evidence for a physical process bounding the M_{MAX} at St1. The KS-test and MLE-test provide strong evidence for the existence of an M_{MAX}. This is also corroborated by the simple analysis (Text S5.2.2; Figure S12). We have summarized these results in Table S1.

5.3 Results from FORGE

5.3.1 Clustering justifications for FORGE

The judicious handling of earthquake clustering is important for correctly interpreting the bound/unbound growth of magnitudes in a sequence. Here, we provide some justifications for our clustering choices. We consider information from both seismological data and hydraulic stimulations to support our clustering choices.

The operations at FORGE took place over two main periods: the first in 2022 that stimulated the first 3 stages of the 16A well and the second in 2024 that stimulated the remaining 7 stages (including a restimulation of stage 3). For reference, we have plotted the timings/locations of earthquakes/stages at FORGE based on our clustering choices (Figures S14 & S15).

Partly, this choice of clustering is driven by the spacing between stages and the amount/spacing of perforations within a stage (Figure S14). The first two toe stages (*i.e.*, stages 1-2) start with simple (single) perforations within the open-hole segment of the well. The following stages (*i.e.*, stages 3-6) reduce the inter-stage distances (~15 m), this means these stages act as a hydraulically linked system. Correspondingly, the microseismic locations show connections between these stages (Figure S15). Stage 6 encountered difficulties during stimulation due to the tightness of the host rock: the first perforation set was unable to achieve formation breakdown. Because of this, a second set of impromptu perforations were used to amend the stimulation plans, without success. This encounter has been interpreted as a geological barrier. Ultimately, the stage 6 plans were shifted to an *ad hoc* stage 10. The remaining stages (*i.e.*, stages 7-10) used increasingly complex multi-perforations, creating a hydraulically linked system for the heel stages. Correspondingly, the microseismic locations show connections between these heel stages. We note that our stage/cluster associations are consistent with other studies [Niemz et al., 2025].

Together, these results provide the justification for our separation of the FORGE catalogue into 3 hydraulically independent clusters: stages 1-2, stages 3-6, and stages 7-10 (with stage 6 delineating a hydraulic barrier).

5.3.2 Simple M_{MAX} indicators at FORGE

First, we consider the earthquake catalogue for the 2022 and 2024 stimulations of well 16A at FORGE. The fit to the GR-MFD is based on the catalogues from Geo-Energie Suisse [Dyer et al., 2023]. There events were detected using STA/LTA approaches and then located with a grid search. Large events were manually repicked. Here, we only consider the subset of well-constrained events (*i.e.*, with L2 relocation labels). To account for possible errors in magnitudes, the GR-MFD fitting follows a 50-trial bootstrap process, in which the catalogue magnitudes are dithered by ±0.1 (this includes a dithered truncation magnitude). For 2022, the Mc is -1.20. For 2024, the Mc is +0.15. Shifting the Mc by ±0.2 does not meaningfully change the results here (or in the following subsections). The fits to both datasets are well-constrained (Figure S16). The *b*-values are 1.13±0.02 and 1.78±0.05, for the 2022 and 2024, respectively. The *b*-values are reported as the mean and standard deviation of bootstrapped results. Both catalogues are suggestive of an M_{MAX} influencing the overall catalogues, since there is an appreciable visual deficiency (or skew) in the number of large magnitude earthquakes. For 2022 and 2024, the quantified δM_{LRG} discrepancy is -1.29 M_W and +0.02 M_W, respectively. This is suggestive of an M_{MAX} influencing parts of the FORGE catalogue.

Next, we separate these catalogues into five distinct clusters: cluster 1 (stages 1-2, 2022), cluster 2a (stage 3, 2022), cluster 2 (stages 3-6, 2024), and cluster 3 (stages 7-10, 2024). The previously outlined workflow is used to fit each of these datasets and produces well-constrained results (Figure S17). The *b*-values are 1.55±0.07, 1.08±0.03, 1.70±0.08, and 1.73±0.08 for clusters 1-3, respectively. All clusters (except cluster 3) are suggestive of an M_{MAX} influencing the overall catalogues, since there is an appreciable visual deficiency in the number of large magnitude earthquakes. For clusters 1-3, the quantified δM_{LRG} discrepancy is -0.76 M_W, -1.31 M_W, -0.43 M_W, and +0.11 M_W, respectively. Assuming unbound catalogues, these would be <1st, <1st, ~1st, and ~50th percentile events, respectively. These further results are also suggestive of an M_{MAX} influencing the catalogues at FORGE.

Further CAP-tests will be needed, to provide more robust and reliable estimates of M_{MAX} beyond these initial suggestions.

5.3.3 CAP-test results for FORGE

For FORGE, we again separate the catalogue into the discrete clusters described previously. The KS-test is performed 50 times, in which the catalogue magnitudes are dithered

(including a dithered truncation magnitude). The KS-test also performs 100 reshuffles. The geometric means of KS-test *p*-value compliments are >99.99%, >99.99%, 99.55%, and 65.34%, for clusters 1-3, respectively. Similarly, we do 50 MLE-tests using dithered catalogues and 100 reshuffles. All clusters (except cluster 3) indicate a fitted M_{MAX} near the value of M_{LRG}, with standard deviations <0.01 M_W.

Next, we perform the EW-tests on the aforementioned FORGE clusters. Catalogues are taken as all the earthquakes that occur during the period between the start of stage stimulation until the start of an out-of-cluster stage. Earthquake magnitudes are truncated based on the Mc. We then propose three M_{MAX} models to explain the data: linearly proportional to volume [McGarr, 2014], proportional to volume with a 3/2 exponent [Galis et al., 2017], and unbound as the null hypothesis. Bound M_{MAX} models are fit to the data, such that the smallest difference between predicted M_{MAX} and observed M_{LRG} is 0.1 M_W. The results of the EW-tests are shown in plots (Figures S18-S21). The McGarr-like model is >100, >100, 39, and 0.02 times more likely than the unbound model for each cluster, respectively. These are strong indications for the McGarr-like M_{MAX} model explaining the data better than the unbound model. The Galis-like model is 1.8, >100, >100, and 0.01 times more likely than the unbound model for each cluster, respectively. In most bound cases, the McGarr-like model is favoured over the Galis-like model. Cluster 2 (2024) prefers the Galis-like model to McGarr-like model, although this is likely biased by not including the events from stage 3 in 2022.

Together, these results are strong (and consistent) evidence for some process bounding the M_{MAX} at FORGE clusters 1-2. The KS-test and MLE-test provide strong evidence for the existence of an M_{MAX}. This is also corroborated by the simple analysis (Text S5.3.2; Figures S16 & S17). The EW-test begins to provide some evidence for the functional form of M_{MAX}, hinting at the potential physics underlying the bounding process. Of the clusters tested, only cluster 3 does not show evidence for M_{MAX}. All the other cases indicate an M_{MAX}. We have summarized these results in Table S1.

5.4 Results from PNR-2

5.4.1 Clustering justifications for PNR-2

The stimulations at PNR-2 took place over roughly 2 weeks' time in August of 2019. For reference, we have plotted the timings/locations of earthquakes/stages at PNR-2 based on our clustering choices (Figure S22).

Our choice of clustering is largely driven by the locations/timings of the microseismicity data recorded. Two separate fault structures have been reactivated there: a ~NW-SE striking western-most structure near the toe of the well and a ~N-S striking eastern-most structure closer to the heel of the well (Figure S22). Roughly, stage 4 delineates the start of fault reactivation for the eastern-most structure. The largest earthquakes take place on this eastern-most cluster. This clustering interpretation is the same as prior studies [Kettlety et al., 2021].

Together, these results provide the justification for our separation of the PNR-2 catalogue into 2 discrete clusters: stages 1-3, and stages 5-7 (with stage 4 delineating the boundary).

5.4.2 Simple M_{MAX} indicators at PNR-2

First, we consider the whole catalogue for the PNR-2 stimulations. The fit to the GR-MFD is based on the catalogues available online [Kettlety et al., 2021]. To account for possible errors in magnitudes, the GR-MFD fitting follows a 50-trial bootstrap process, in which the catalogue magnitudes are dithered by ±0.1 (this includes a dithered truncation magnitude). The Mc is -1.0; shifting the Mc by ±0.2 does not meaningfully change the results here (or in the following subsections). The fits to the dataset are well-constrained (Figure S23). The *b*-value is 1.18±0.04. The *b*-values are reported as the mean and standard deviation of bootstrapped results. The full catalogue is suggestive of some unbound process for the overall catalogue, since there is an appreciable visual abundance in the number of large magnitude earthquakes. The quantified δM_{LRG} discrepancy is +0.86 M_W. Together, these are suggestive of an unbound process for the largest events in the PNR-2 catalogue.

Next, we separate these catalogues into two distinct clusters: the western-most (stages 1-3) and eastern-most (stages 5-7). The fits to each of these datasets are well-constrained (Figure S24), by using the previously outlined workflow. The *b*-values are 1.21±0.05 and 1.14±0.04 for the western/eastern-most clusters, respectively. Only one cluster is suggestive of an M_{MAX} influencing the overall catalogues, since there is an appreciable visual deficiency in the number of large magnitude earthquakes. For the western- and eastern-most clusters, the quantified δM_{LRG} discrepancy is -1.26 M_W and +0.84 M_W, respectively. Assuming unbound catalogues, these would

be <1st and ~89th percentile events, respectively. These results are suggestive of an M_{MAX} influencing the initial parts of the catalogue at PNR-2, but not the later parts.

Further CAP-tests will be needed, to provide robust and reliable estimates of M_{MAX} beyond these initial suggestions.

5.4.3 CAP-test results for PNR-2

For PNR-2, we again separate the catalogue into the same 2 clusters described previously. The KS-test is performed 50 times, in which the catalogue magnitudes are dithered (including a dithered truncation magnitude). The KS-test also performs 100 reshuffles. The geometric means of KS-test p-value compliments are >99.99%, and 33.7%, for the western-most and eastern-most clusters, respectively. Similarly, we do 50 MLE-tests using dithered catalogues and 100 reshuffles. The western-most cluster has a fitted M_{MAX} near the value of M_{LRG}, with a standard deviation <0.01 M_W. The eastern-most cluster is unable to adequately fit M_{MAX}, with standard deviation of ~2.5 M_W

Next, we perform the EW-tests on the aforementioned PNR-2 clusters. Catalogues are taken as all the earthquakes that occur during the period between the start of stage stimulation until the start of an out-of-cluster stage. Earthquake magnitudes are truncated based on the Mc. We then propose three M_{MAX} models to explain the data: linearly proportional to volume [McGarr, 2014], proportional to volume with a 3/2 exponent [Galis et al., 2017], and unbound as the null hypothesis. Bound M_{MAX} models are fit to the data, such that the smallest difference between predicted M_{MAX} and observed M_{LRG} is 0.1 M_W. The results of the EW-tests are shown in plots (Figures S25-S26). The McGarr-like model is >100 and 0.01 times more likely than the unbound model for western-most and eastern-most clusters, respectively. The Galis-like model is ~70, and <0.01 times more likely than the unbound model for western-most and eastern-most clusters, respectively. This is a strong indication for the McGarr-like M_{MAX} model explaining the data better than the unbound model for the western-most cluster.

Together, these PNR-2 results are strong (and consistent) evidence for a transition from a process bounding the M_{MAX} for the western-most cluster into an unbound process for the eastern-most cluster. The KS-test and MLE-test provide strong evidence for the existence of an M_{MAX} in the western-most case, but not the eastern-most case. This is also corroborated by the simple analysis (Text S5.4.2; Figures S23-S24). The EW-test begins to provide some evidence for the

functional form of M_{MAX}, hinting at the potential physics underlying the bounding process. We have summarized these results in Table S1.

5.5 Supporting results from Soultz-sous-Forêts

Here we detail additional supporting cases that also show evidence for bound growth of induced earthquake magnitudes. These cases span the operations at Soultz-sous-Forêts (SSFS) in France. SSFS is an EGS operations in the Rhine Graben of France (Figure S7), aimed at extracting geothermal energy from the granitic basement [Dorbath et al., 2009]. Hydraulic stimulations were performed periodically since the 1990s. One of the largest events encountered was M_W 2.9 [Dorbath et al., 2009]. The catalogues used in this study are readily available online [Leptokaropoulos et al., 2019] for the 1993, 2000, 2003, 2004, and 2005 stimulations. In particular, we use the homogenized moment magnitude catalogue from a recent study [Drif et al., 2024].

We have chosen not to focus on this case in the main text of this article largely because of suspicions of catalogue artifacts. In the data, we note that there are large discretization of magnitudes, apparent temporal gaps, and potentially upper magnitude truncation. These artifacts likely obfuscate our ability to make a clean bound/unbound discernment. With these caveats in mind, we share these results that appear to favour bound interpretations for SSFS.

5.5.1 Clustering choice for SSFS

We consider all events to be hydraulically linked within a single period. Subsequent periods are considered as independent.

5.5.2 Simple M_{MAX} indicators at SSFS

To assess the potential for bound/unbound earthquake growth, we first employ the simple GR-MFD analyses. Overall, each of the five considered stimulations appears to be visually deficient in large magnitude events (Figures S27, S29, S31, S33, & S35). To fit each GR-MFD, Mc of -0.50, 0.10, 0.20, -0.90, and -0.20 are used, respectively. The fits to the dataset are constrained with a *b*-values of 1.11±0.04, 1.12±0.07, 0.79±0.04, 0.55±0.03, and 0.58±0.02, respectively. Correspondingly, the δM_{LRG} discrepancies are -0.62 M_W, -1.09 M_W, -1.21 M_W, -3.10

M_W, and -2.80 M_W, respectively. These initial assessments are suggestive of some M_{MAX} upper bound restricting the catalogue growth for SSFS stimulations.

5.5.3 CAP-test results for SSFS

Next, we use the CAP-tests to detect and assess the potential for M_{MAX} more rigorously. The KS-test is performed 50 times, in which the catalogue magnitudes are dithered (including a dithered truncation magnitude). The KS-test also performs 100 reshuffles. The geometric means of KS-test *p*-value compliments are >99.99%, >99.99%, >99.99%, >99.99%, and >99.99%, respectively. Similarly, we do 50 MLE-tests using dithered catalogues and 100 reshuffles. The fitted M_{MAX} has a standard deviation of <0.01 M_W, <0.01 M_W, 0.03 M_W, 0.01 M_W, and 0.02 M_W for each of the SSFS stimulations, respectively. Together, KS-test and MLE-test results show strong evidence for an M_{MAX}.

Next, we perform the EW-tests on the SSFS data. Earthquake magnitudes are truncated based on the Mc. We then propose three M_{MAX} models to explain the data: linearly proportional to volume [McGarr, 2014], proportional to volume with a 3/2 exponent [Galis et al., 2017], and unbound as the null hypothesis. Bound M_{MAX} models are fit to the data, such that the smallest difference between predicted M_{MAX} and observed M_{LRG} is 0.1 M_W. The results of the EW-tests are shown in plots (Figures S28, S30, S32, S34, & S36). Respectively, the McGarr-like model is >100, >100, ~2.6, >100, and >100 times more likely than the unbound model. Respectively, the Galis-like model is >100, ~0.2, ~0.2, >100, and >100 times more likely than the unbound model. This is another strong indication for the bound models explaining the data better than the unbound model.

Together, these SSFS results are consistent evidence for a physical process bounding the M_{MAX} at SSFS. The KS-test and MLE-test provide strong evidence for the existence of an M_{MAX}. This is corroborated by the simple analysis. The EW-test begins to provide some evidence for the functional form of M_{MAX}, hinting at the potential physics underlying the bounding process. We have summarized these results in Table S1.

5.6 Summary of results

Here we organize our results for all cases, clusters, and tests – for convenience to the reader. These results are summarized below (Table S1).

Case		Simple tests		CAP-tests			
**Location**	**Cluster**	***b*-value**	**δM_{LRG}**	**KS-test**	**MLE-test**	**EW-test**	**M_{MAX} model**
PNR-1z	*All*	1.25±0.04	-0.47	99.82%	<0.01	>100	Galis
St1	*All*	1.31±0.06	-0.69	98.17%	0.03	>100	McGarr/Galis
FORGE	*1*	1.55±0.07	-0.76	>99.99%	<0.01	>100	McGarr
FORGE	*2a*	1.08±0.03	-1.31	>99.99%	<0.01	>100	McGarr
FORGE	*2*	1.70±0.08	-0.43	99.55%	<0.01	~26	Galis
FORGE	*3*	1.73±0.08	+0.11	65.34%	~0.80	0.02	Unbound
PNR-2	*West*	1.21±0.05	-1.24	>99.99%	<0.01	>100	McGarr/Galis
PNR-2	*East*	1.14±0.04	+0.84	33.7%	~2.50	0.01	Unbound
SSFS	*1993*	1.11±0.04	-0.62	>99.99%	<0.01	>100	McGarr
SSFS	*2000*	1.12±0.07	-1.09	>99.99%	<0.01	>100	McGarr
SSFS	*2003*	0.79±0.04	-1.21	>99.99%	0.03	~2.6	McGarr
SSFS	*2004*	0.55±0.03	-3.10	>99.99%	0.01	>100	McGarr
SSFS	*2005*	0.58±0.02	-2.80	>99.99%	0.02	>100	McGarr

Table S1. Summary of study results. All the prior results of our simple tests and CAP-tests are compiled here for convenience. Additionally, we have coordinated individual entries according to their interpretation: blue for bound, pink for unbound, and uncoloured for indeterminate.

Text S6: Sensitivity of CAP-test results to perturbations

Here we do additional sensitivity testing on the results of our CAP-tests, to build further confidence about the veracity of our results. The two perturbations we consider are an expanded set of hypothesized M_{MAX} models and the substitution of a new catalogue. Both sensitivity tests are performed on the data from FORGE for the 2022 stage 3 (*i.e.*, cluster 2a) stimulation.

6.1 Expanded EW-testing of M_{MAX} hypotheses on Stage 3 (2022) at FORGE

The first sensitivity test considers a perturbation to the list of candidate M_{MAX} models. We consider the data already available in the fit to the spatiotemporal expansion of induced earthquakes. This study highly scrutinized the 2022 stage 3 stimulation at FORGE, fitting the spatiotemporal data to three models: assuming a pore pressure diffusion process, an aseismically propagating fault, and the propagation of a penny-shaped crack. The spatiotemporal data were fit to the functional forms expected from each of these three models, providing potential explanations for the expansion of the seismicity front. Each of these models were then translated into an estimated M_{MAX} via the finite fault size, assuming a constant stress drop of 3 MPa, and a simple scaling relationship [Hanks & Kanamori, 1979]. In summary, the size of the seismicity front is assumed to place bounds on how large earthquakes could grow.

We include these three additional M_{MAX} models in a repetition of our CAP-tests, specifically the EW-test since this depends on the choice of candidate models input (Figure S37). Similar to prior results, we find that all of these bound models outperform the unbound null hypothesis. Of all of the models' input, the McGarr-like model currently explains the data best. These results are in correspondence to those found in the main text. Because of this, we do not repeat fits of the other models to the spatiotemporal data to include them as additional M_{MAX} models in the EW-tests.

6.2 Catalogue substitution on Stage 3 (2022) at FORGE

The second sensitivity test perturbs the input catalogue, to accommodate the possibility of processing artifacts. Here, we use an alternative catalogue of events for the 2022 stage 3 stimulation at FORGE. This catalogue uses the same waveform dataset but used a different (machine learning based) workflow to build the catalogue [Shi et al., 2022].

We apply the same set of CAP-tests to this catalogue. The geometric means of our KS-test *p*-value compliments are >99.99%. The MLE-test is able to quantify the value of M_{MAX} confidently (*i.e.*, <0.01 M_W). The EW-test also produces similar results (Figure S38). We find that the bound models are significantly more likely than the unbound model and that the M_{MAX} which is linearly proportional to volume explains the data best. Overall, the results from this machine learning based catalogue are the same as those derived from the GES catalogue. Based on this, the results of the main text document focus on the catalogue from GES. While the machine learning based catalogue is more complete, CAP-tests are predominantly sensitive to magnitudes of events approaching M_{MAX}. Thus, additional completeness in smaller magnitude events doesn't necessarily improve M_{MAX} resolving power, depending on the M_{MAX} model. Additional details on sensitivity testing can be found in prior works [Schultz, 2024].

Text S7: CAP-test subtleties and the need for accurate earthquake-stage clustering

Here we highlight some of the subtleties of our analysis. Largely, this section highlights results that occur when stages are hydraulically linked (but the user fails to link them accurately). These parts are included for any readers who may follow-up with our work. This section provides some cautionary points for interpretation and utilization. We feel that these subtleties also bolster the overall interpretation we have provided in the main text.

7.1 Example at PNR-1z

7.1.1 Appropriately recognizing hydraulically linked stages at PNR-1z

The first test is to show that the sequencing of earthquakes is important. We demonstrate this on the PNR-1z data. When all the stages at PNR-1z are considered linked, our CAP-tests showed that this cluster is bound (Text S5.1; Figures 3 & S10). Correspondingly, if we considered the first stage as independent, then we still arrive at the same result. The geometric means of our KS-test *p*-value compliments are >99.99%. The MLE-test is able to quantify the value of M_{MAX} confidently. The EW-test also produces similar results (Figure S39), with an odds ratio of >100 favouring the bound models. Overall, the results from this first stage agree with the full cluster results, just with a lesser degree of statistical confidence. Similarly, we can repeat this process, keeping only the first *X* stages (Table S2). All of these subset CAP-tests show the same bound result; it is noteworthy that statistical confidence generally increases alongside the increase of data.

We interpret this as due to the first stage starting the process of fracture stimulation; thus, this stage appears to be bound, since event magnitudes are restricted by the size of the fracture network. Similarly, all subsequent stages continue the fracturing process, expanding the fracture network, allowing for larger magnitudes with time. Thus, the inclusion of linked stages together continues to increase the statistical confidence from the CAP-tests.

7.1.2 Failing to recognize hydraulically linked stages at PNR-1z

Next, we cover issues that arise from failing to recognize when multiple stages are hydraulically linked into a single cluster. For example, if we failed to recognize that the first five stages at PNR-1z were linked with the subsequent stages, then this could lead to diminished interpretations. The geometric means of our KS-test *p*-value compliments are 98.9%. The EW-test is less confident in a bound relationship (Figure S40) with an odds ratio of ~9. Importantly,

the observation of a bound inference (via EW-tests) only starts to become apparent after the M_{LRG} from the first five stages (*i.e.*, 0.90 M_W) is surpassed. For completeness's sake, we repeat this process, ignoring the first *X* stages (Table S2). Generally, statistical confidence shrinks as more stages are omitted; eventually, we are unable to discern the bound process at PNR-1z, after enough stages are omitted.

The reason for how this result arises is consistent with our overall interpretation. The initial stages start creating the fracture network; this bound fracture network restricts the magnitude of large events. Subsequent (hydraulically linked) stages continue to expand this fracture network, building upon the stimulations of prior stages. This means that if the first *X* stages were (erroneously) ignored, then the apparent 'growth' of these earthquakes would appear as unbound – since the fracture network is already pre-existing. Any subsequent events would only start to appear as bound after it reached M_{LRG} magnitudes greater than those from the initial/ignored stages. It appears that mixed/conflicting individual CAP-test results could be indicative of this type of error (Table S2).

These results highlight the subtleties and importance of accurately quantifying hydraulic connections during operations. They also flag immediate concerns for the proper identification of bound cases. Accurately capturing smaller magnitude events appears to be important for EW-tests, when considering volume-based M_{MAX} models. Fortunately, this requirement is effectively limited to the M_{LRG} sequence. Thus, future operations using CAP-tests would likely benefit from increased quality control when cataloging the M_{LRG} event sequence.

Cluster Definition		KS-test	MLE-test	EW-test	M_{LRG}
Start Stage	End Stage			odds ratio	
1	1	>99.99%	<0.01	>100	0.44 M_W
1	2	>99.99%	<0.01	>100	0.44 M_W
1	3	>99.99%	<0.01	>100	0.70 M_W
1	12	>99.99%	<0.01	>100	0.90 M_W
1	13	>99.99%	<0.01	>100	0.90 M_W
1	14	>99.99%	<0.01	>100	1.10 M_W
1	18	93.05%	<0.01	>100	1.10 M_W
1	22	>99.99%	<0.01	>100	1.10 M_W
1	30	>99.99%	<0.01	>100	1.30 M_W
1	31	>99.99%	<0.01	>100	1.40 M_W
1	32	99.56%	<0.01	>100	1.60 M_W
1	35	99.80%	<0.01	>100	1.60 M_W
1	37	>99.99%	<0.01	>100	1.60 M_W
1	38	99.62%	<0.01	>100	1.90 M_W
1	39	99.88%	<0.01	>100	1.90 M_W
1	40	99.80%	<0.01	>100	1.90 M_W
1	41	99.86%	<0.01	>100	1.90 M_W
2	41	99.50%	<0.01	~55	1.90 M_W
3	41	99.80%	<0.01	~16	1.90 M_W
12	41	98.20%	<0.01	~0.03	1.90 M_W
13	41	98.70%	<0.01	~9	1.90 M_W
14	41	97.80%	<0.01	~19	1.90 M_W
18	41	98.40%	<0.01	~0.04	1.90 M_W
22	41	94.26%	<0.01	~4.6	1.90 M_W
30	41	87.35%	<0.01	~0.17	1.90 M_W
31	41	58.90%	<0.01	~0.05	1.90 M_W
32	41	55.08%	0.02	~0.04	1.90 M_W
35	41	62.78%	<0.01	~0.09	1.90 M_W
37	41	52.25%	<0.01	~0.03	1.90 M_W
38	41	59.84%	0.04	~0.03	1.90 M_W
39	41	69.31%	0.01	~0.56	1.90 M_W
40	41	44.30%	0.02	~0.02	1.90 M_W
41	41	64.72%	0.01	~0.02	1.90 M_W

Table S2. Clustering subset analysis. We report the EW-test results when keeping only the first/last *X* stages at PNR-1z. Note that some planned stages were skipped, so the stage numbers 'jump' in their ordering. Additionally, we have coordinated individual entries according to their interpretation: blue for bound, pink for unbound, and uncoloured for indeterminate.

<u>Text S8: Comparing event likelihoods for bound/unbound induced seismicity sequences</u>

To display the potential differences in bound/unbound cases, with respect to their potential for hazards from large magnitude events, we quantify a hypothetical scenario. Here, we impose a traffic light protocol where the red-light is at M_W 3.0 and the yellow light is at M_W 1.0. Specifically, we contrast bound/unbound stages that are otherwise identical. Both stages have a *b*-value of 1.0, seismogenic index of -2.0 [Shapiro et al., 2010], and a volume of 10^4 m^3 injected, resulting in an expected value of 10^2 events induced or an *a*-value of 2.0. One stage is bounded at an M_{MAX} of 2.5, while the other is unbound. The differences between the cumulative GR-MFD are significant for these cases (Figure S41). Both cases would expect an M_{LRG} somewhere around M_W 2.0-2.2 with a 50% probability of exceedance. However, the likelihood of encountering events larger than this diverges significantly: the unbound case is far more likely to encounter large events, meaning greater potential for ground shaking hazards. The unbound case also has the potential to exceed the red-light threshold (~10%), while the bound does not. An operator encountering this situation would benefit from being able to discern between these two possibilities (*e.g.*, via CAP-tests).

We note that this hypothetical example is highly simplistic. We have provided it only to illustrate a simple point. We acknowledge that it differs from the bounded cases we have already examined, in that the M_{MAX} value increases alongside the progression of stage stimulations.

Supplementary Figures

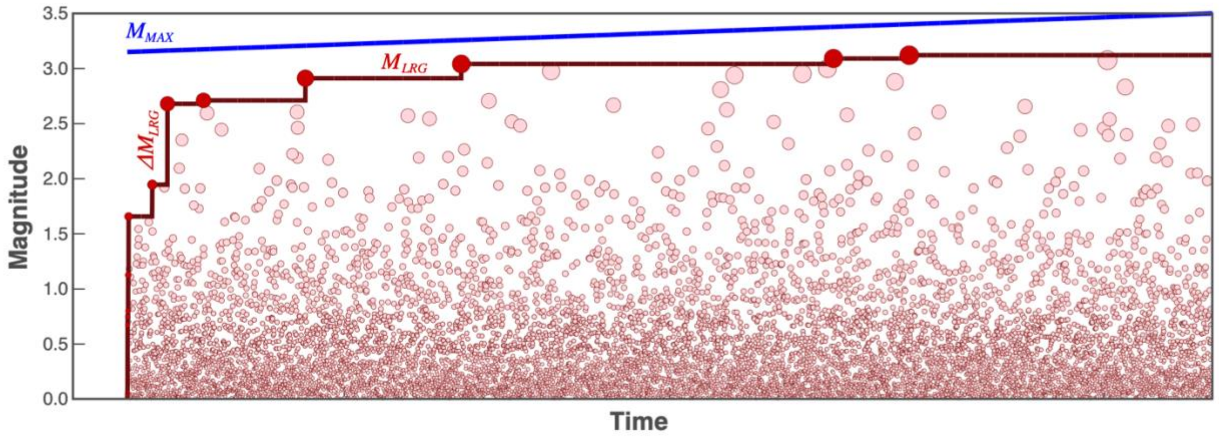

Figure S1. Visual definition of terminology for an earthquake sequence. A catalogue of earthquakes (red/pink circles) is order into the largest observed earthquakes M_{LRG} (red lines). Earthquake magnitudes could be bounded by a maximum possible value M_{MAX} (blue line).

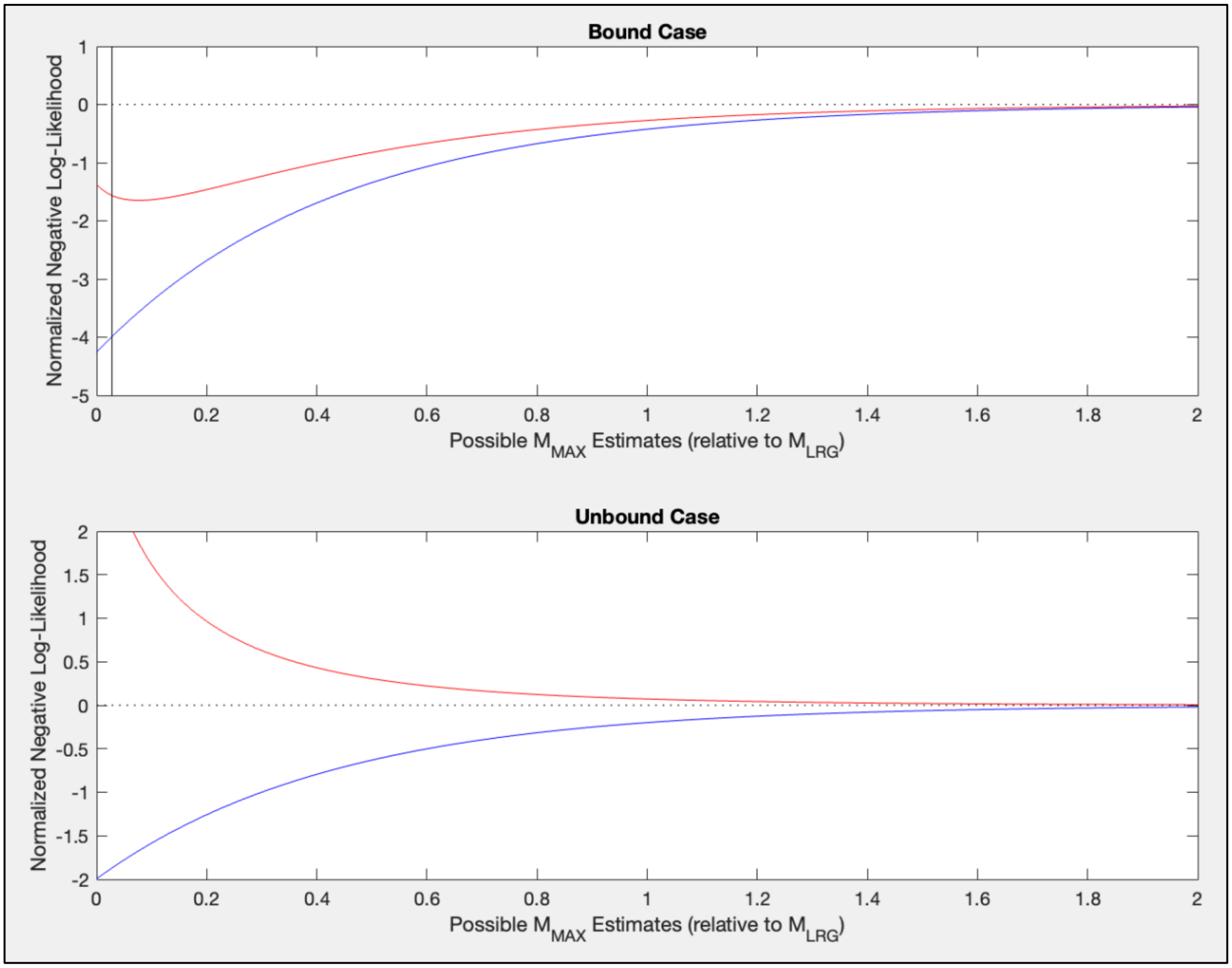

Figure S2. Synthetic examination of the log-likelihood. The negative log-likelihood as a function of M_{MAX} possibilities, both for the 'standard' GR-MFD (blue lines) and when including the ΔM_{LRG} term (red lines). Since the log-likelihoods are normalized, anything below zero (dashed line) indicates an M_{MAX} fitting the better than unbound. This test is repeated for a bound (top panel) and unbound (bottom panel) catalogue. The true M_{MAX} value is shown (black line).

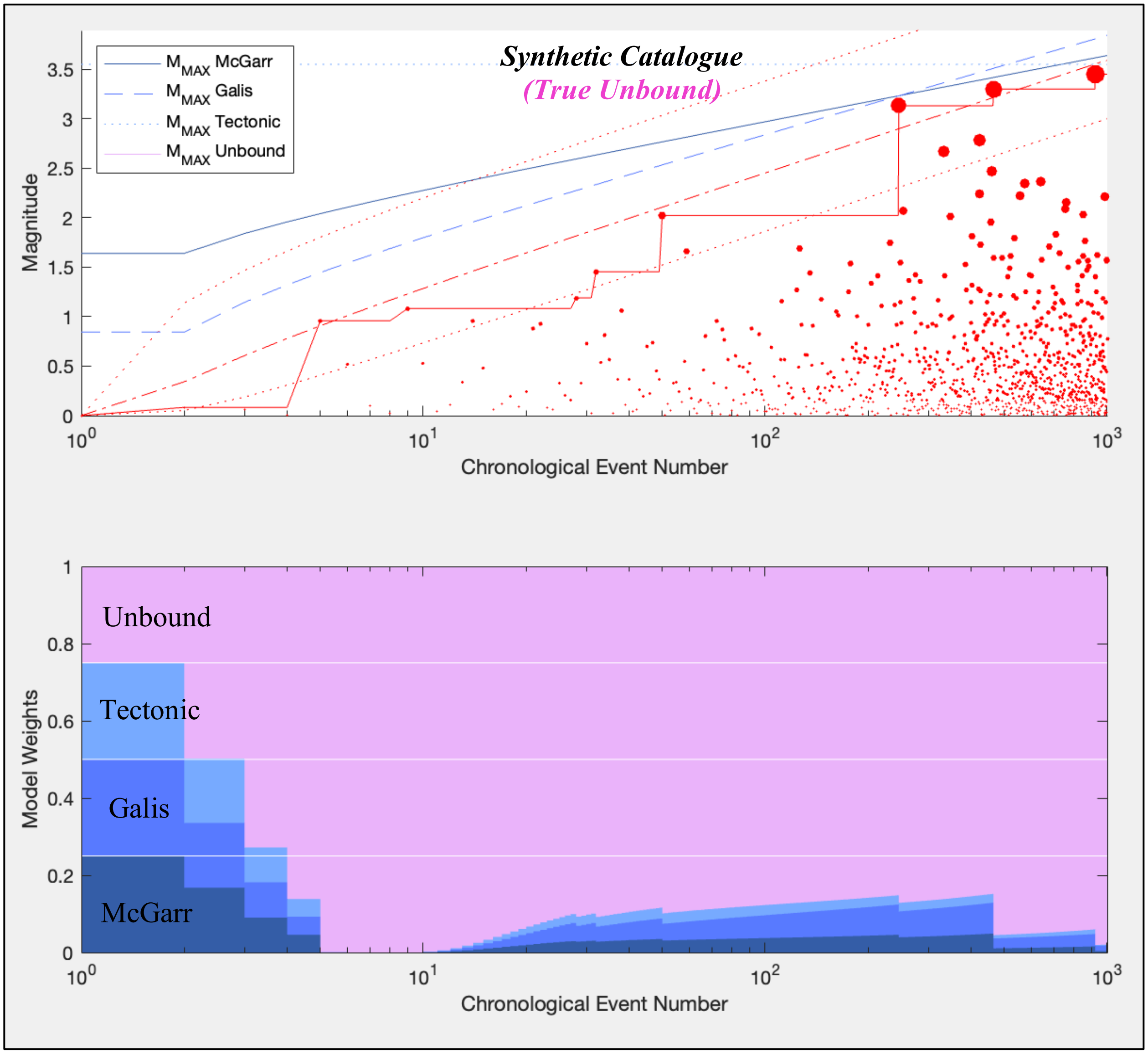

Figure S3. Using the EW-test to discern between M_{MAX} models for a synthetic catalogue (unbound true). In the top panel, the catalogue of earthquake magnitudes (red circles), the observed M_{LRG} sequence (red lines & circles), and expected M_{LRG} at the 10/50/90 percentiles (red dashed lines) are tested using four M_{MAX} assumptions (blue lines). In the bottom panel, AIC/BIC-based ensemble model weights (coloured bars) using all data prior to each new event are shown.

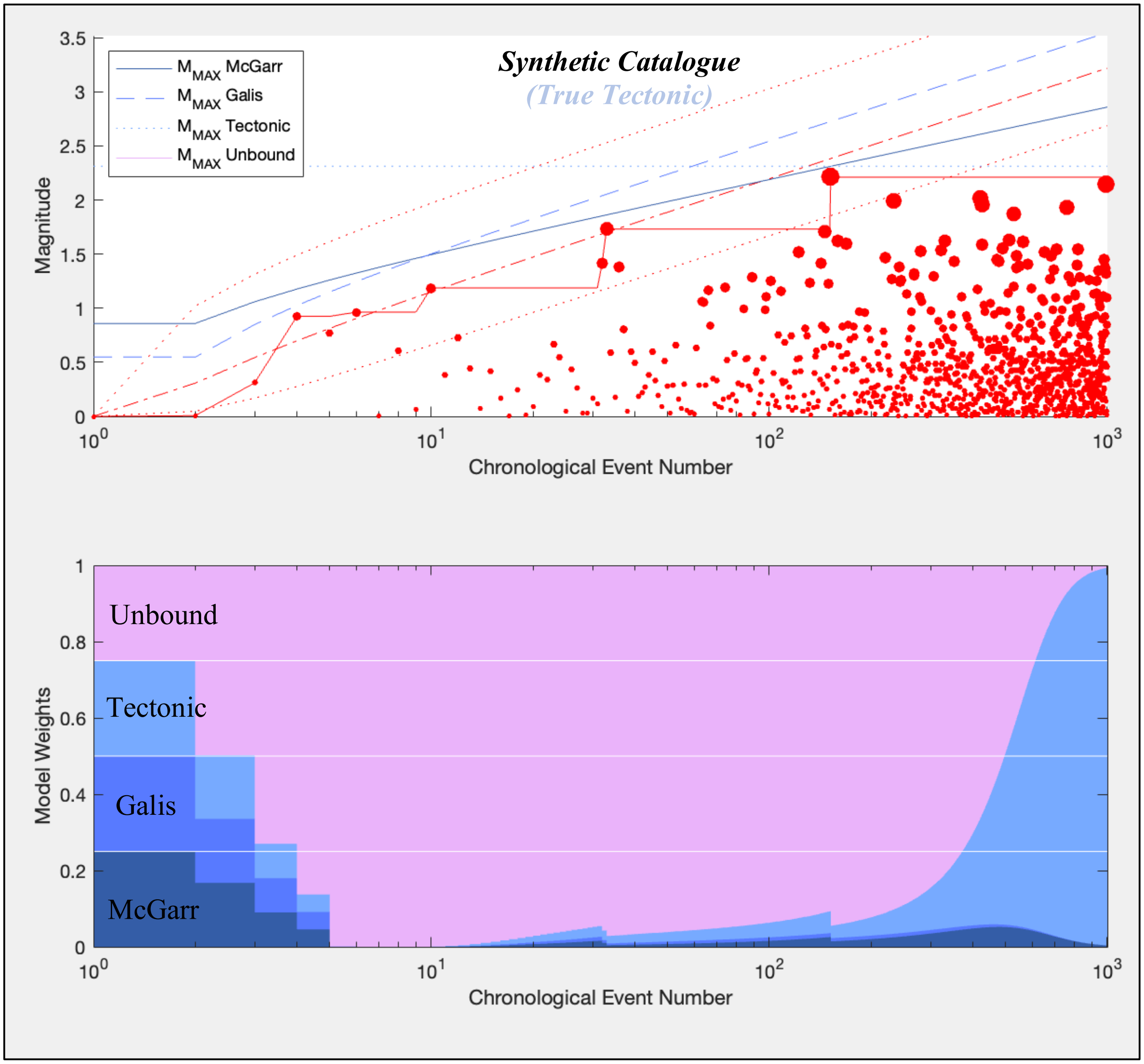

Figure S4. Using the EW-test to discern between MMAX models for a synthetic catalogue (tectonic true). In the top panel, the catalogue of earthquake magnitudes (red circles), the observed MLRG sequence (red lines & circles), and expected MLRG at the 10/50/90 percentiles (red dashed lines) are tested using four MMAX assumptions (blue lines). In the bottom panel, AIC/BIC-based ensemble model weights (coloured bars) using all data prior to each new event are shown.

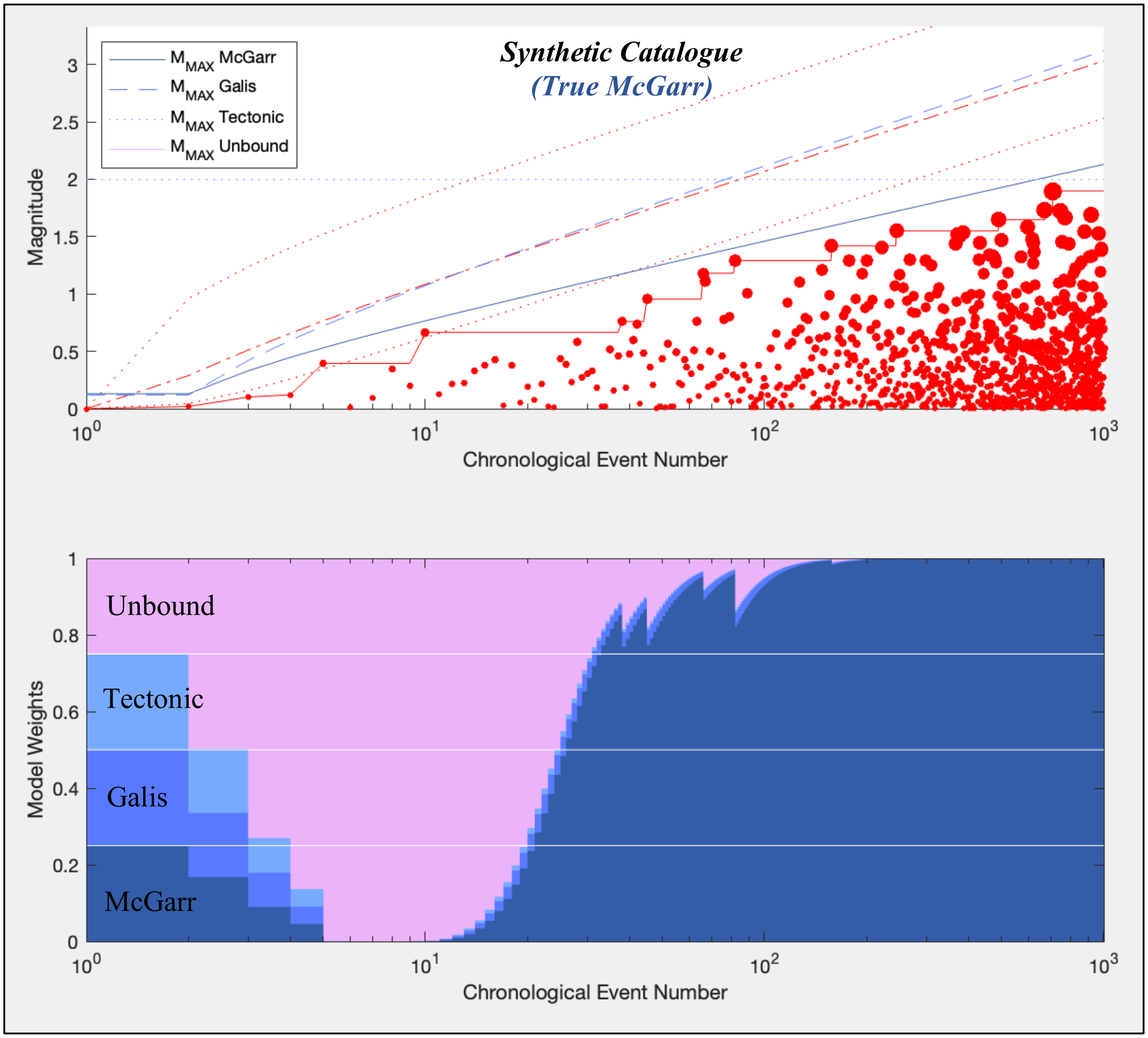

Figure S5. Using the EW-test to discern between M_{MAX} models for a synthetic catalogue (McGarr true). In the top panel, the catalogue of earthquake magnitudes (red circles), the observed M_{LRG} sequence (red lines & circles), and expected M_{LRG} at the 10/50/90 percentiles (red dashed lines) are tested using four M_{MAX} assumptions (blue lines). In the bottom panel, AIC/BIC-based ensemble model weights (coloured bars) using all data prior to each new event are shown.

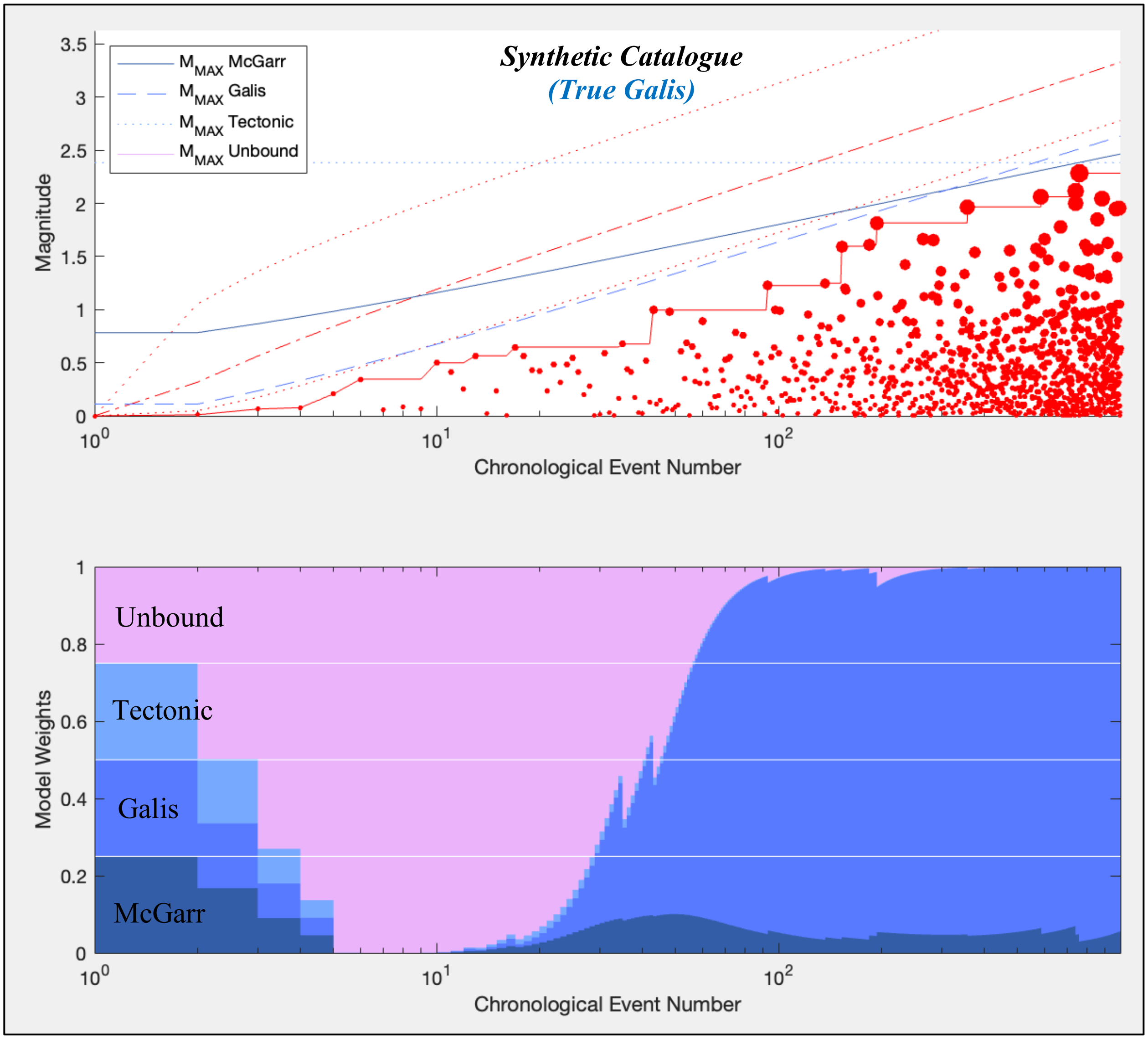

Figure S6. Using the EW-test to discern between M_{MAX} models for a synthetic catalogue (Galis true). In the top panel, the catalogue of earthquake magnitudes (red circles), the observed M_{LRG} sequence (red lines & circles), and expected M_{LRG} at the 10/50/90 percentiles (red dashed lines) are tested using four M_{MAX} assumptions (blue lines). In the bottom panel, AIC/BIC-based ensemble model weights (coloured bars) using all data prior to each new event are shown.

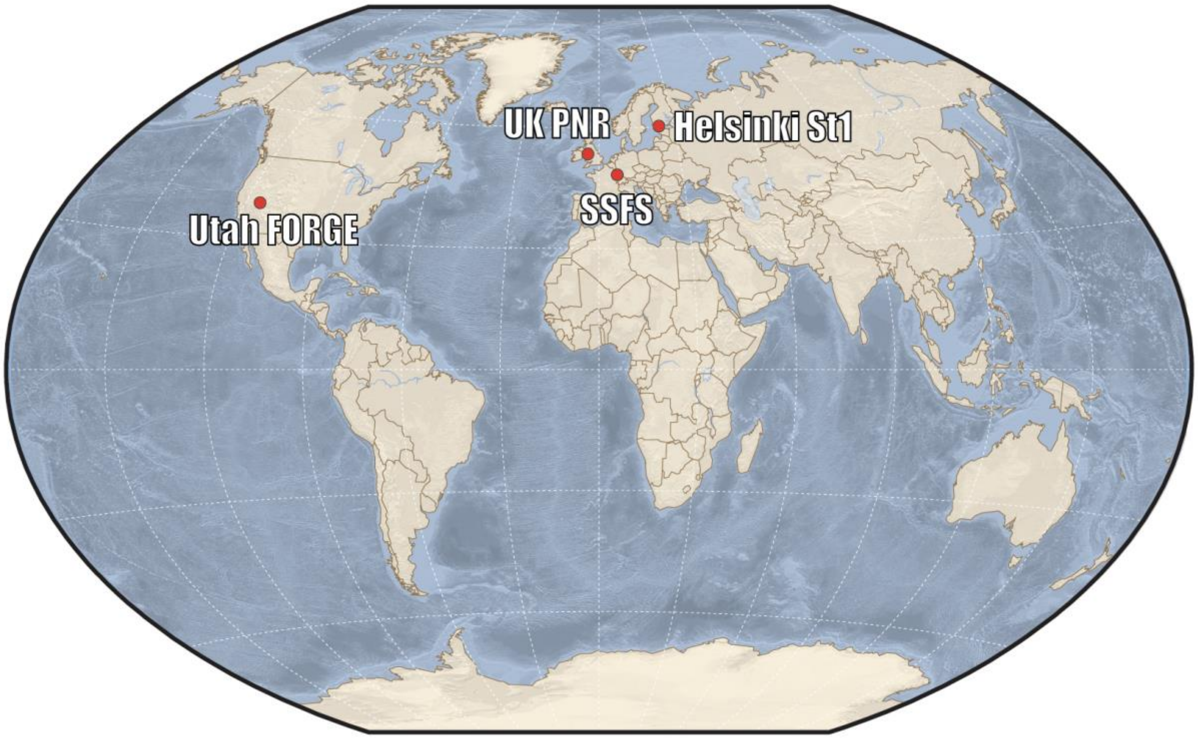

Figure S7. Global locations of datasets. Locations of the test sites considered: the EGS operations at Utah FORGE (USA), shale gas HF at PNR (UK), and EGS operations at Soultz-sous-Forêts (France).

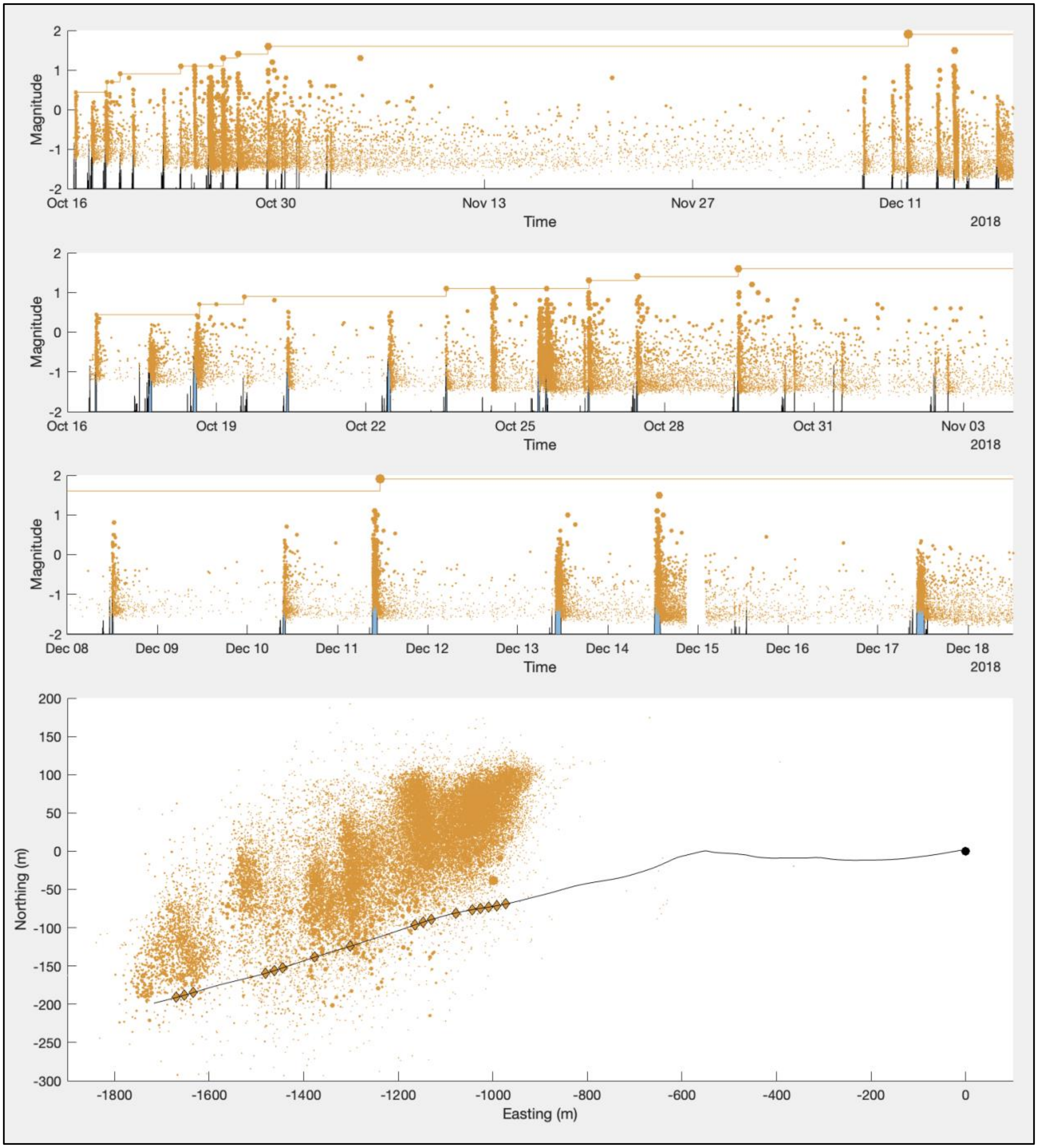

Figure S8. Spatiotemporal clustering of earthquakes during the PNR-1z stimulation. (top panels) The timing of stage stimulations (blue area) against the timings/magnitudes of induced earthquakes (circles) and the sequence of M_{LRG} (coloured line). (bottom panel) The map locations of stage stimulations (diamond) along the well bore (black line), in comparison. In all plots, earthquakes are colour coordinated with their respective clusters.

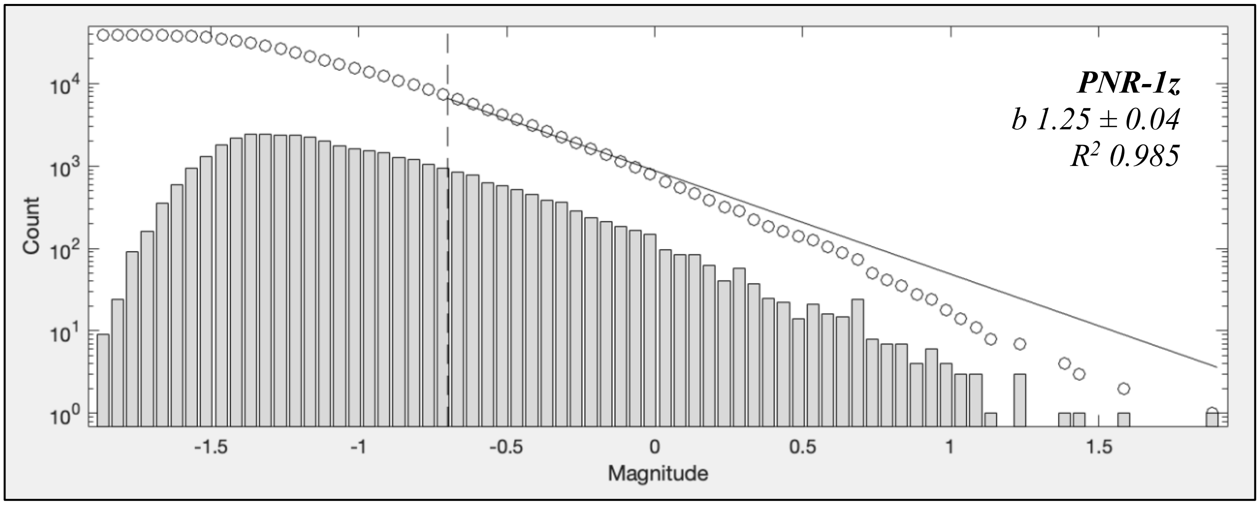

Figure S9. Magnitude statistics at PNR-1z (full). Magnitude frequency distribution of events in the regional catalogue (grey): data counts are plotted as both the cumulative (circles) and non-cumulative (bars) distributions, alongside the best fit to the data (solid line) and the magnitude-of-completeness Mc (dashed line). Data is from the full catalogue at PNR-1z.

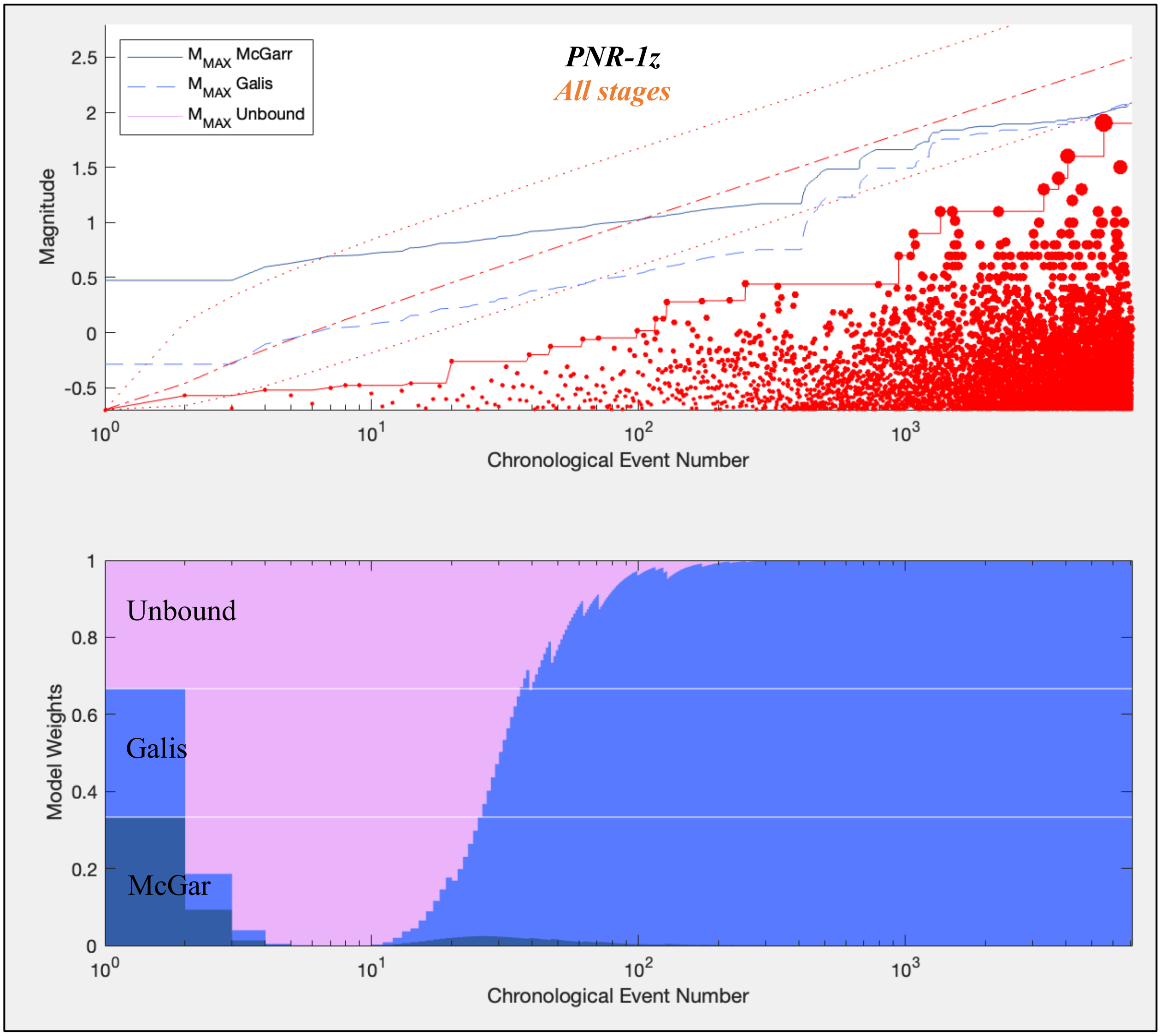

Figure S10. Using the EW-test to discern between M_{MAX} models for all stages at PNR-1z. In the top panel, the catalogue of earthquake magnitudes (red circles), the observed M_{LRG} sequence (red lines & circles), and expected M_{LRG} at the 10/50/90 percentiles (red dashed lines) are tested using three M_{MAX} assumptions (blue lines). In the bottom panel, AIC/BIC-based ensemble model weights (coloured bars) using all data prior to each new event are shown.

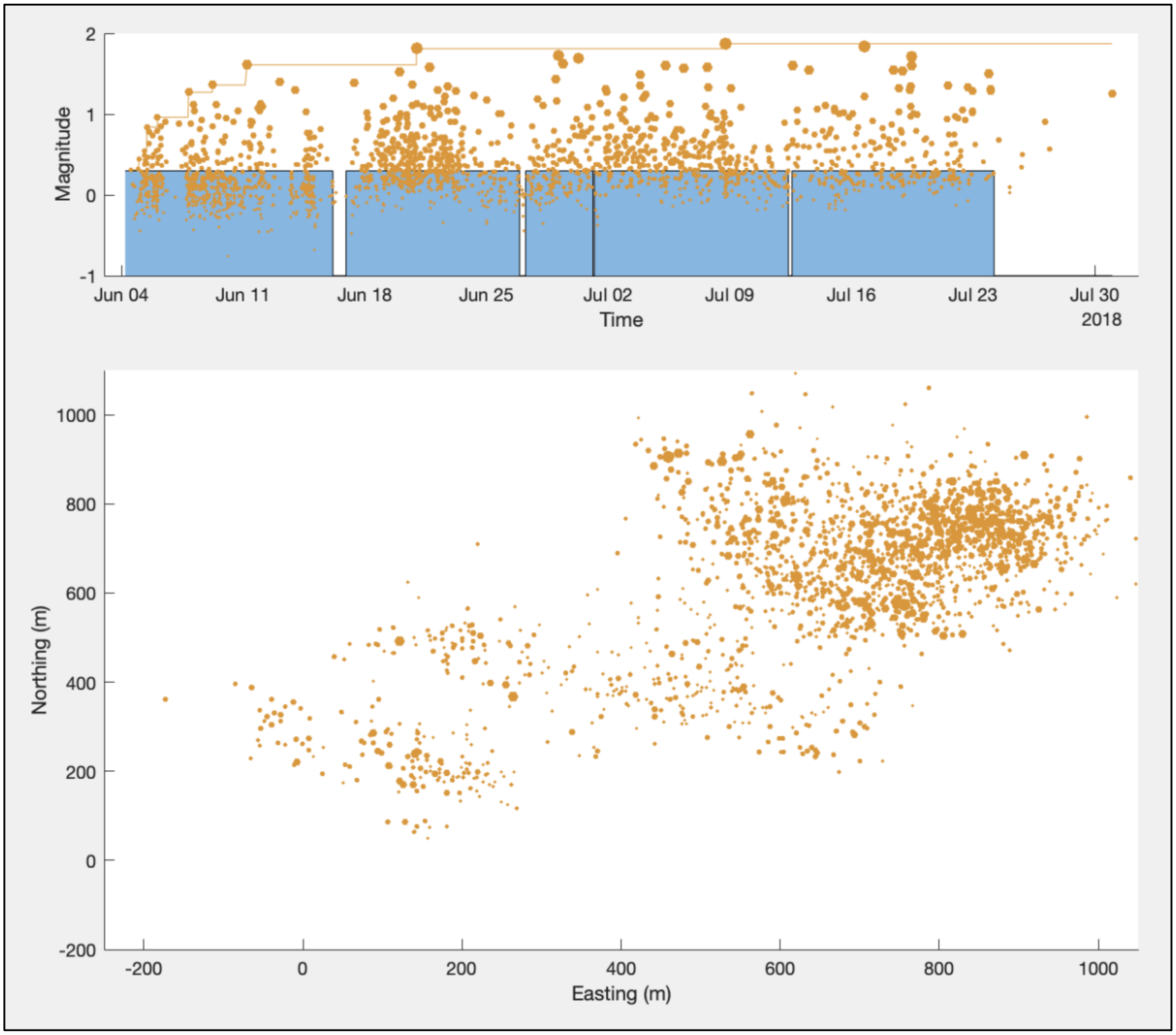

Figure S11. Spatiotemporal clustering of earthquakes during the Helsinki St1 stimulation. (top panels) The timing of stage stimulations (blue area) against the timings/magnitudes of induced earthquakes (circles) and the sequence of M_{LRG} (coloured line). (bottom panel) The map locations of stage stimulations (diamond) along the well bore (black line), in comparison. In all plots, earthquakes are colour coordinated with their respective clusters.

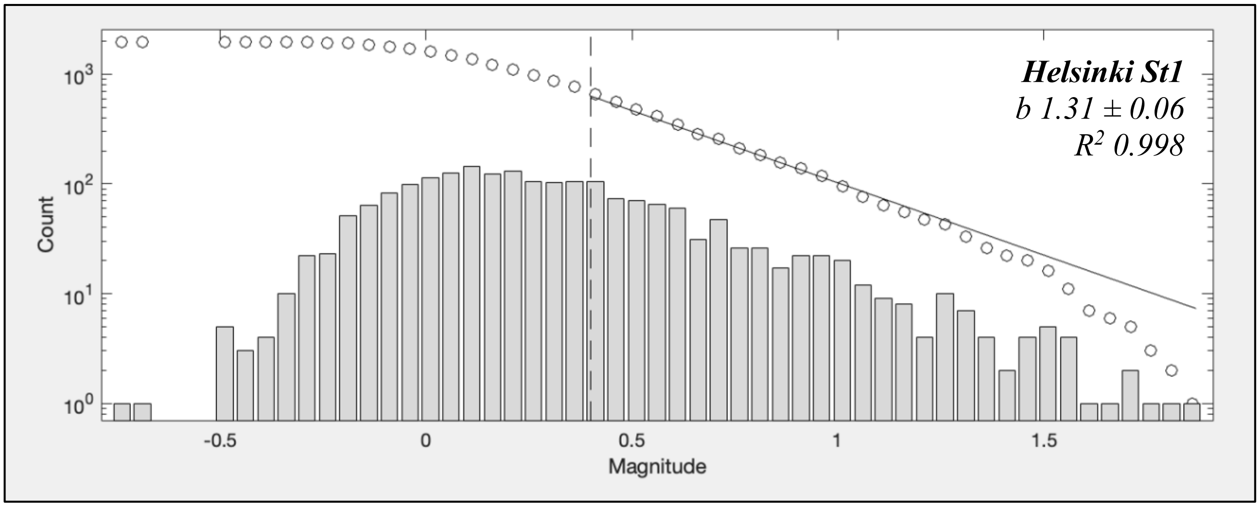

Figure S12. Magnitude statistics at St1 (full). Magnitude frequency distribution of events in the regional catalogue (grey): data counts are plotted as both the cumulative (circles) and non-cumulative (bars) distributions, alongside the best fit to the data (solid line) and the magnitude-of-completeness Mc (dashed line). Data is from the full catalogue at St1.

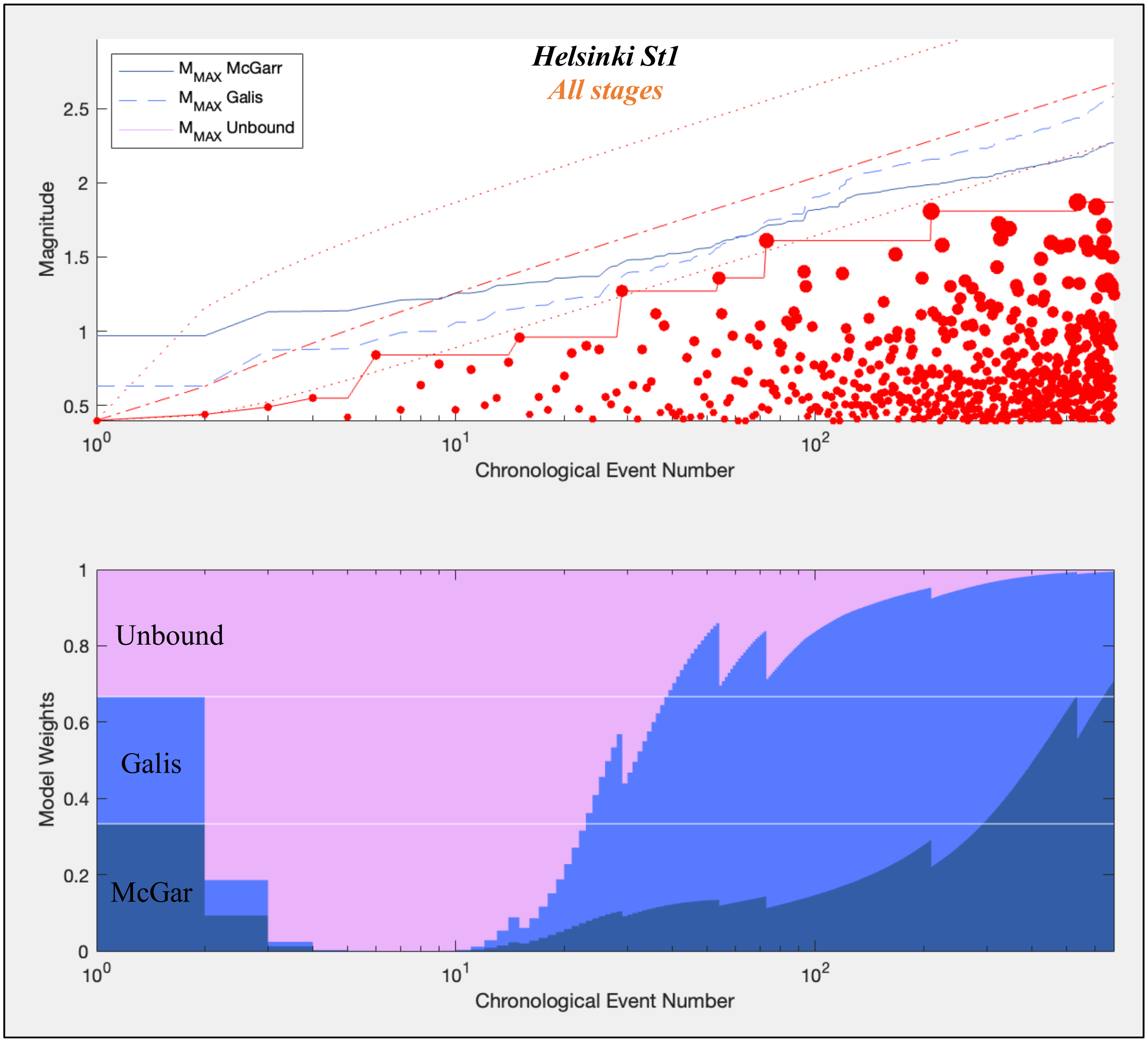

Figure S13. Using the EW-test to discern between M_{MAX} models for all stages at St1. In the top panel, the catalogue of earthquake magnitudes (red circles), the observed M_{LRG} sequence (red lines & circles), and expected M_{LRG} at the 10/50/90 percentiles (red dashed lines) are tested using three M_{MAX} assumptions (blue lines). In the bottom panel, AIC/BIC-based ensemble model weights (coloured bars) using all data prior to each new event are shown.

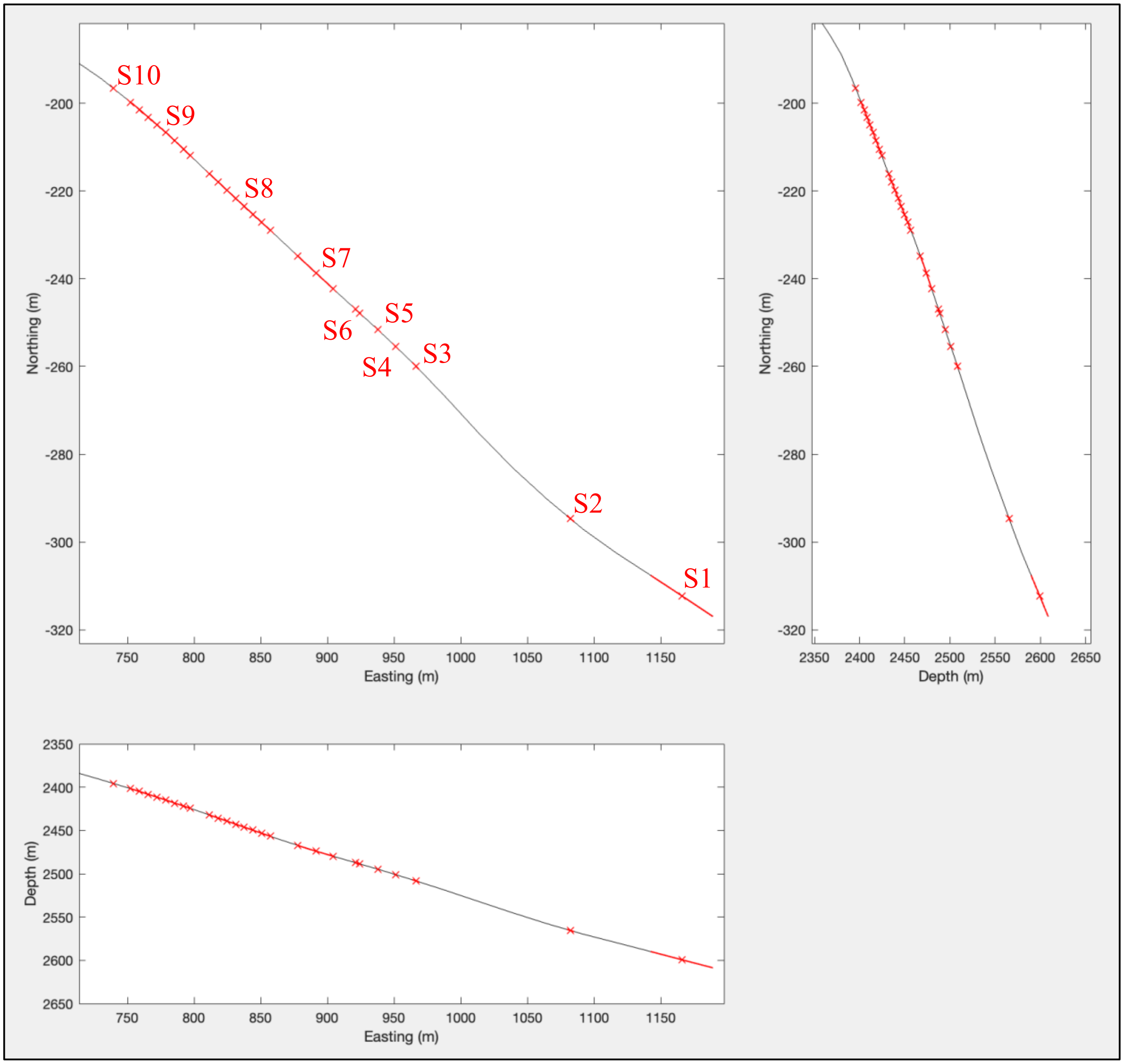

Figure S14. FORGE well 16A completion design. The locations of perforations (red x's) within a single stage (red line) are shown for their locations along the well trajectory (black line). Plotted in map view (top left panel), N-S depth profile (top right panel), and E-W depth profile (bottom left panel).

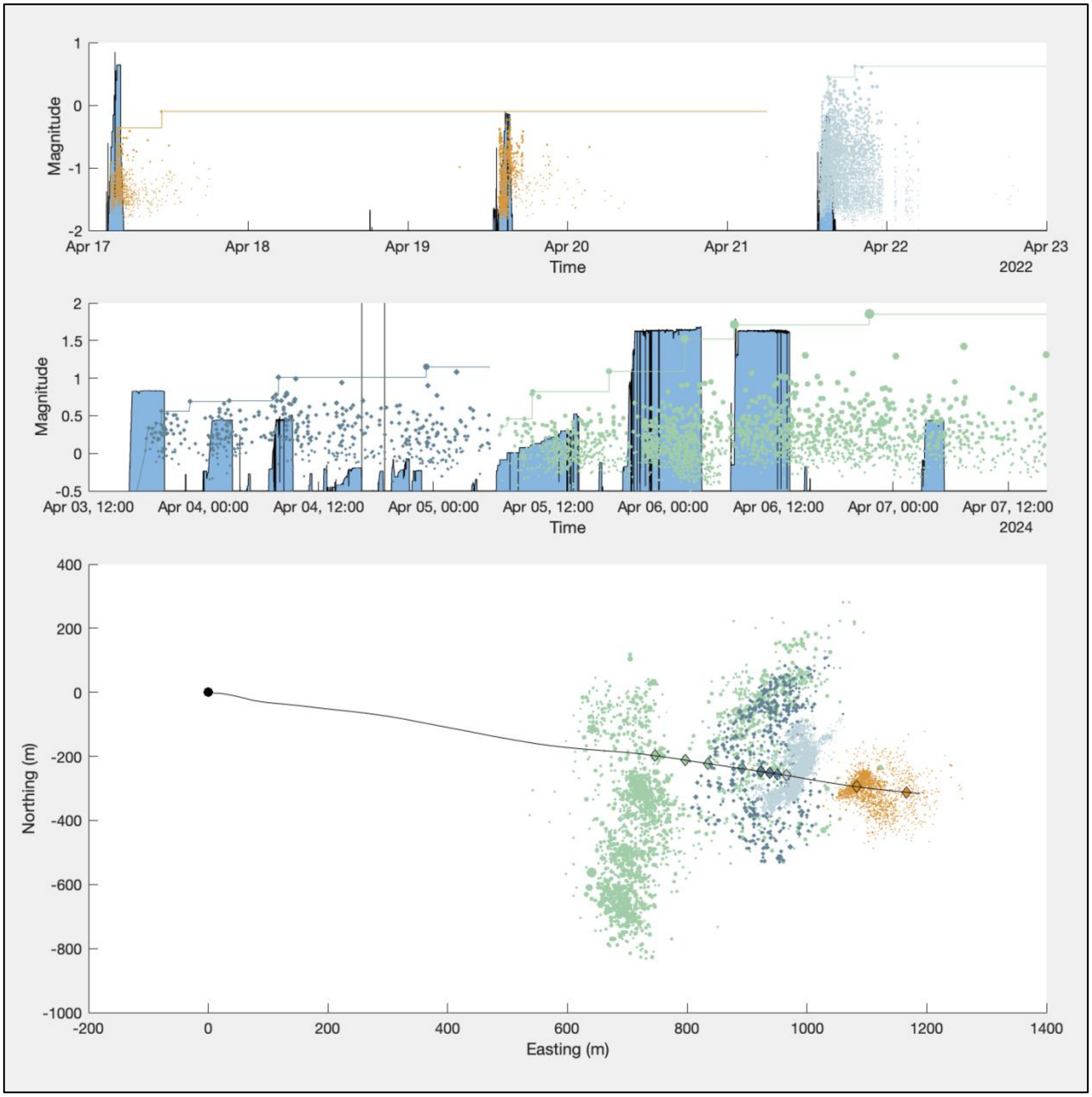

Figure S15. Spatiotemporal clustering of earthquakes during the FORGE stimulation. (top panels) The timing of stage stimulations (blue area) against the timings/magnitudes of induced earthquakes (circles) and the sequence of M_{LRG} (coloured line), for each cluster. (bottom panel) The map locations of stage stimulations (diamonds) along the well bore (black line). In all plots, earthquakes are colour coordinated with their respective clusters.

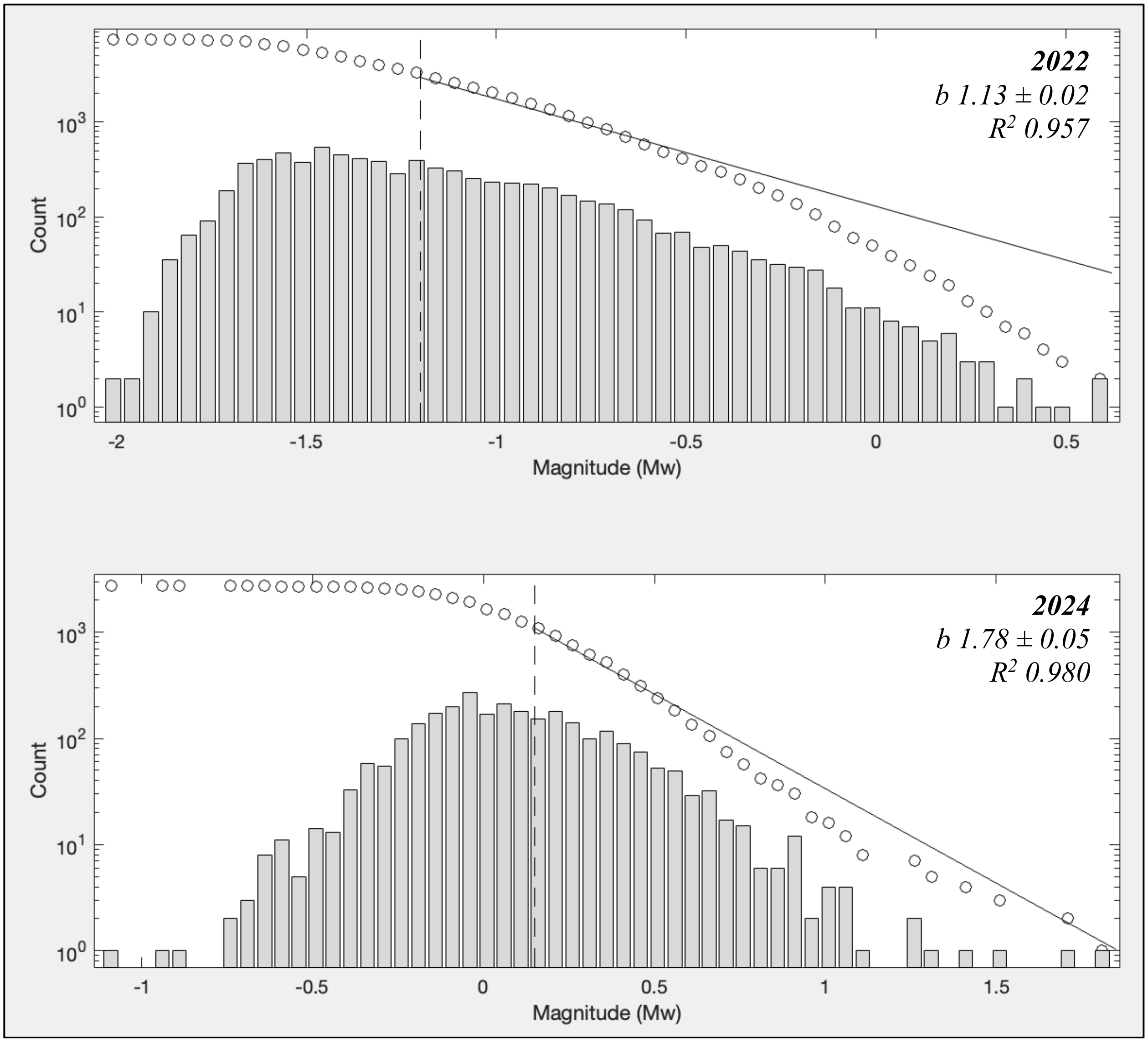

Figure S16. Magnitude statistics at FORGE (full). Magnitude frequency distribution of events in the regional catalogue (grey): data counts are plotted as both the cumulative (circles) and non-cumulative (bars) distributions, alongside the best fit to the data (solid line) and the magnitude-of-completeness Mc (dashed lines). Data is from the full catalogue at FORGE, for the 2022 (top panel) and 2024 (bottom panel) stimulations.

FORGE

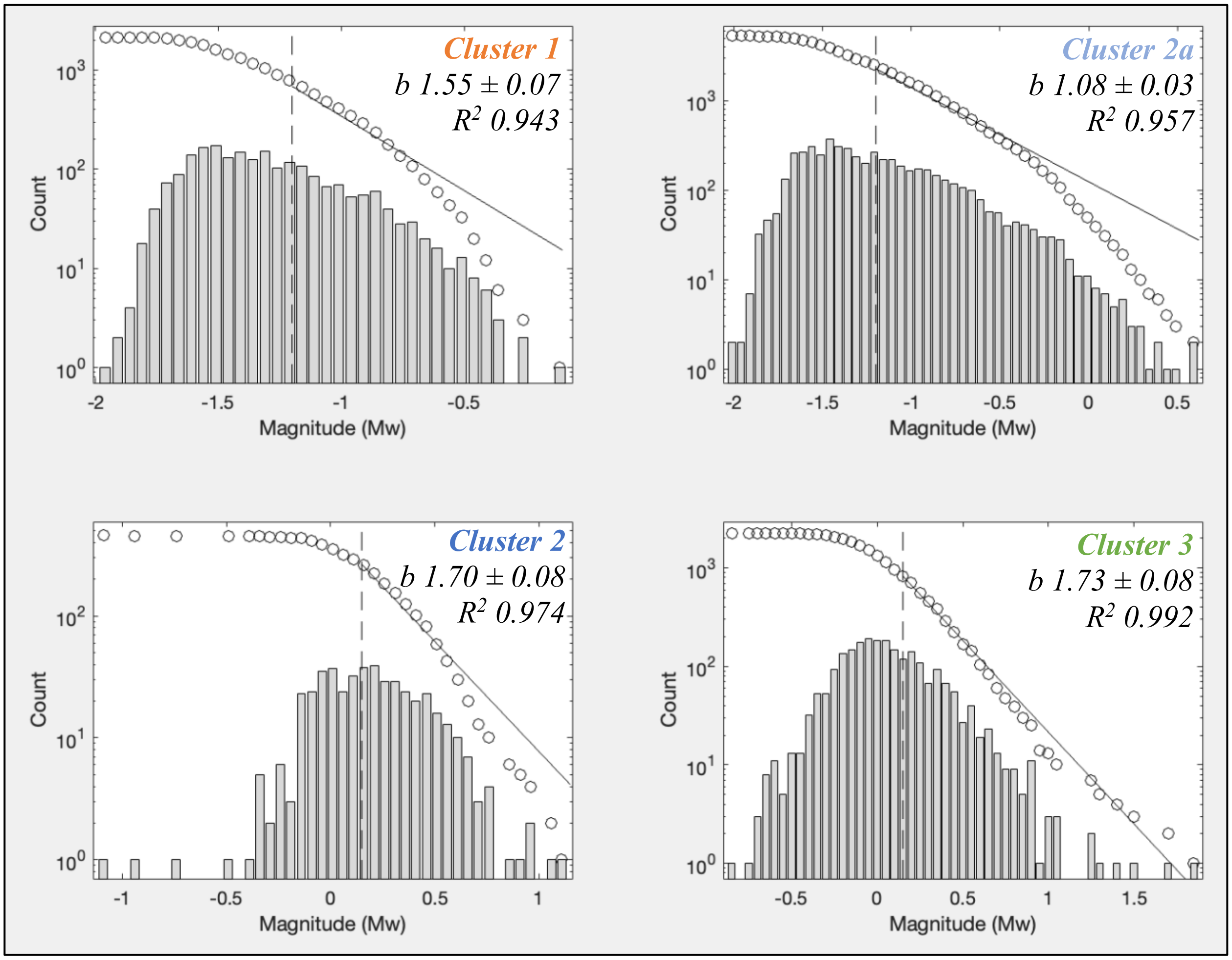

Figure S17. Magnitude statistics at FORGE (clustered). Magnitude frequency distribution of events in the regional catalogue (grey): data counts are plotted as both the cumulative (circles) and non-cumulative (bars) distributions, alongside the best fit to the data (solid line) and the magnitude-of-completeness Mc (dashed lines). Data is from the clustered catalogue at FORGE, for cluster 1 (2022, stages 1-2; top-left panel), cluster 2a (2022, stage 3; top-right panel), cluster 2 (2024, stages 3-6; bottom-left panel), and cluster 3 (2024, stages 7-10; bottom-right panel) stimulations.

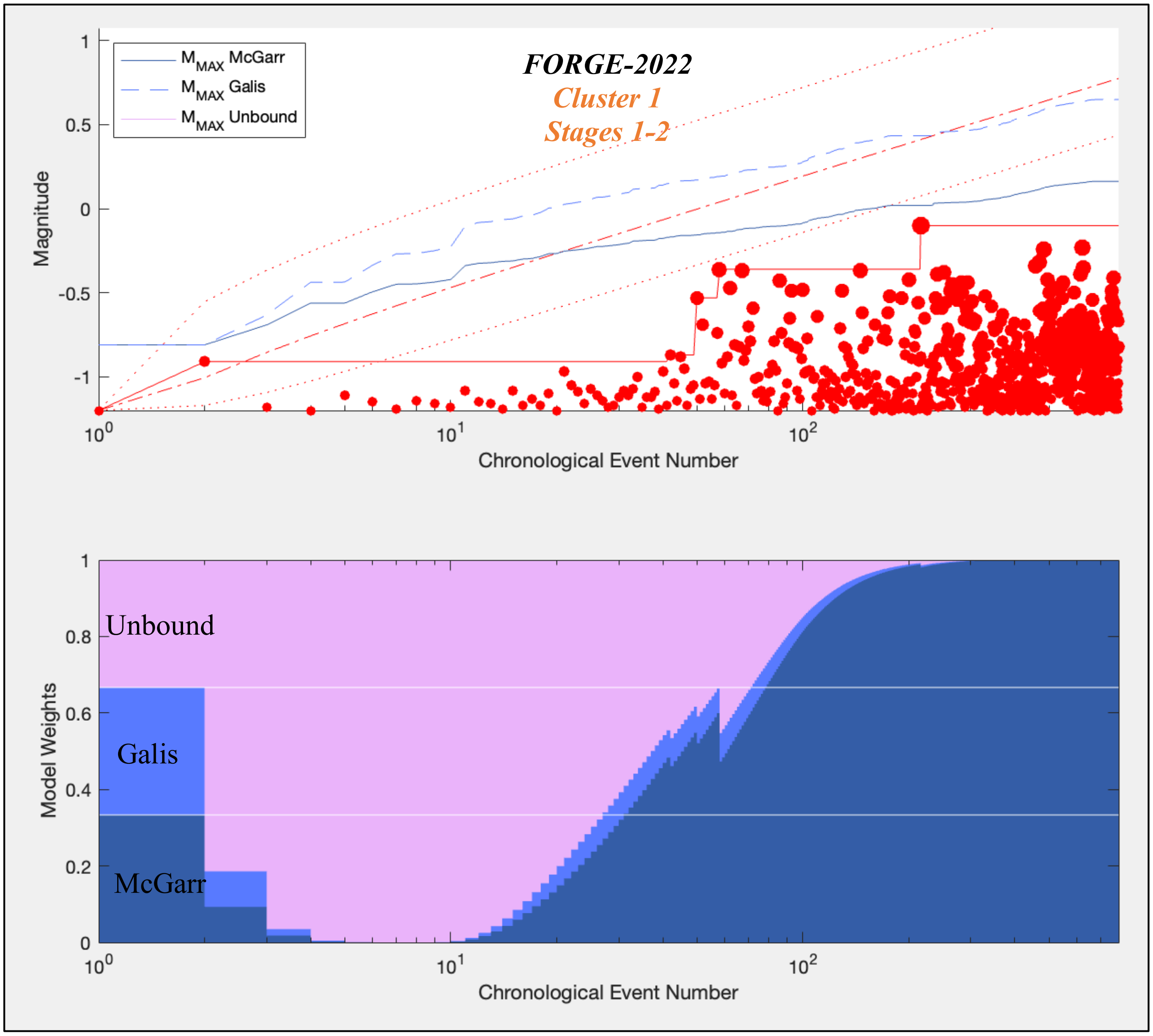

Figure S18. Using the EW-test to discern between M_{MAX} models for cluster 1 at FORGE (2022). In the top panel, the catalogue of earthquake magnitudes (red circles), the observed M_{LRG} sequence (red lines & circles), and expected M_{LRG} at the 10/50/90 percentiles (red dashed lines) are tested using three M_{MAX} assumptions (blue lines). In the bottom panel, AIC/BIC-based ensemble model weights (coloured bars) using all data prior to each new event are shown.

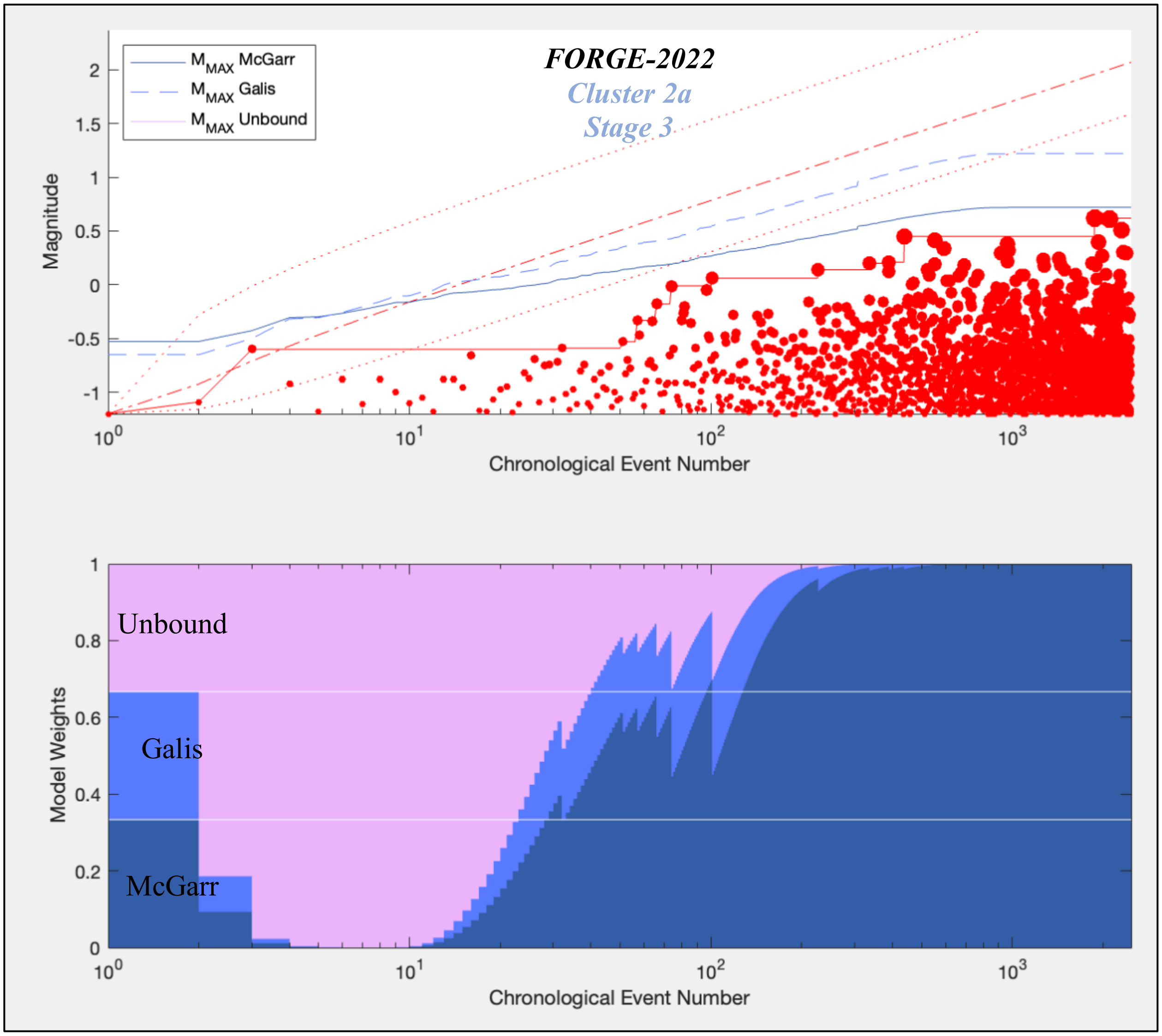

Figure S19. Using the EW-test to discern between M_{MAX} models for cluster 2a at FORGE (2022). In the top panel, the catalogue of earthquake magnitudes (red circles), the observed M_{LRG} sequence (red lines & circles), and expected M_{LRG} at the 10/50/90 percentiles (red dashed lines) are tested using three M_{MAX} assumptions (blue lines). In the bottom panel, AIC/BIC-based ensemble model weights (coloured bars) using all data prior to each new event are shown.

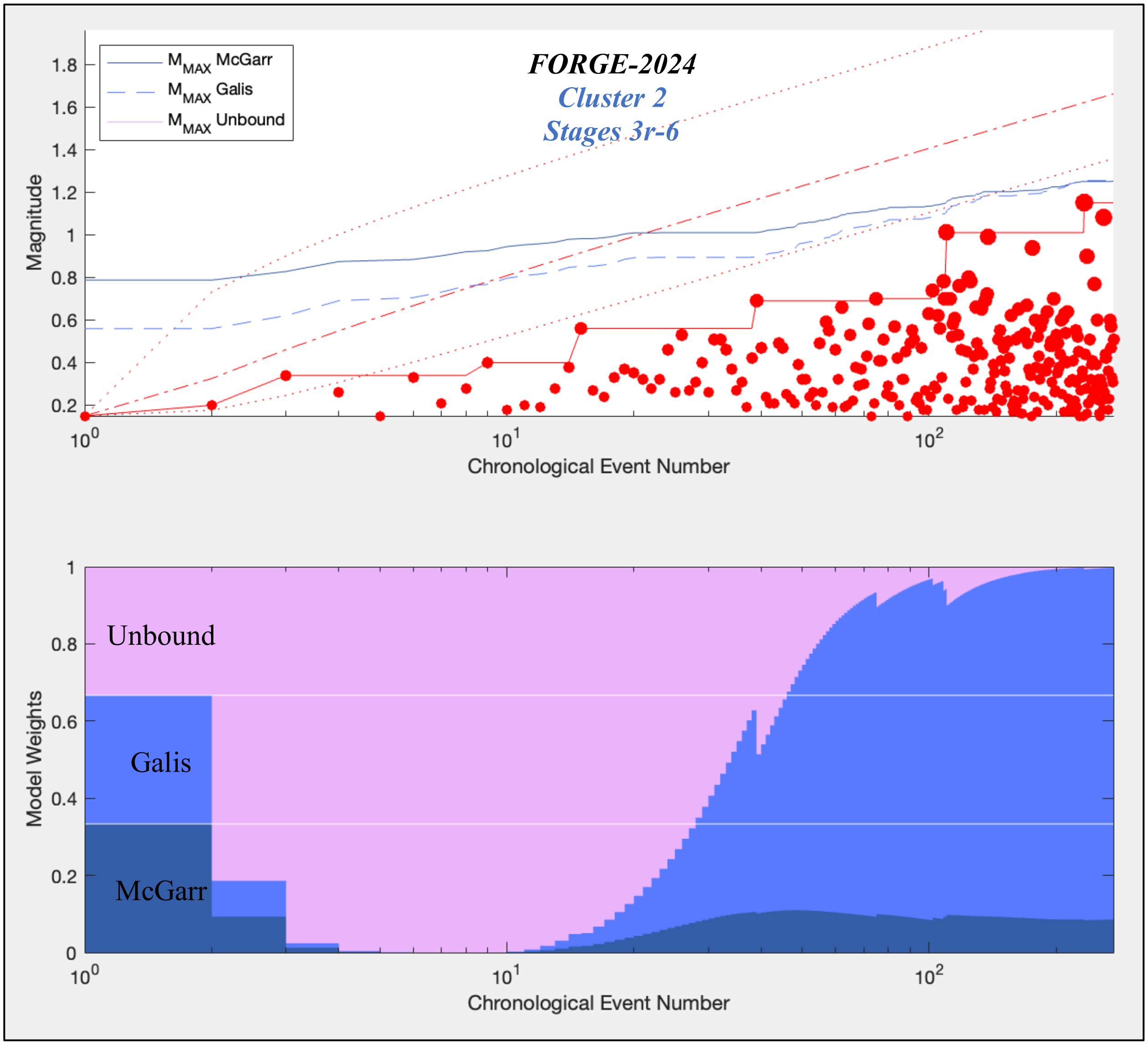

Figure S20. Using the EW-test to discern between M_{MAX} models for cluster 2 at FORGE (2022). In the top panel, the catalogue of earthquake magnitudes (red circles), the observed M_{LRG} sequence (red lines & circles), and expected M_{LRG} at the 10/50/90 percentiles (red dashed lines) are tested using three M_{MAX} assumptions (blue lines). In the bottom panel, AIC/BIC-based ensemble model weights (coloured bars) using all data prior to each new event are shown.

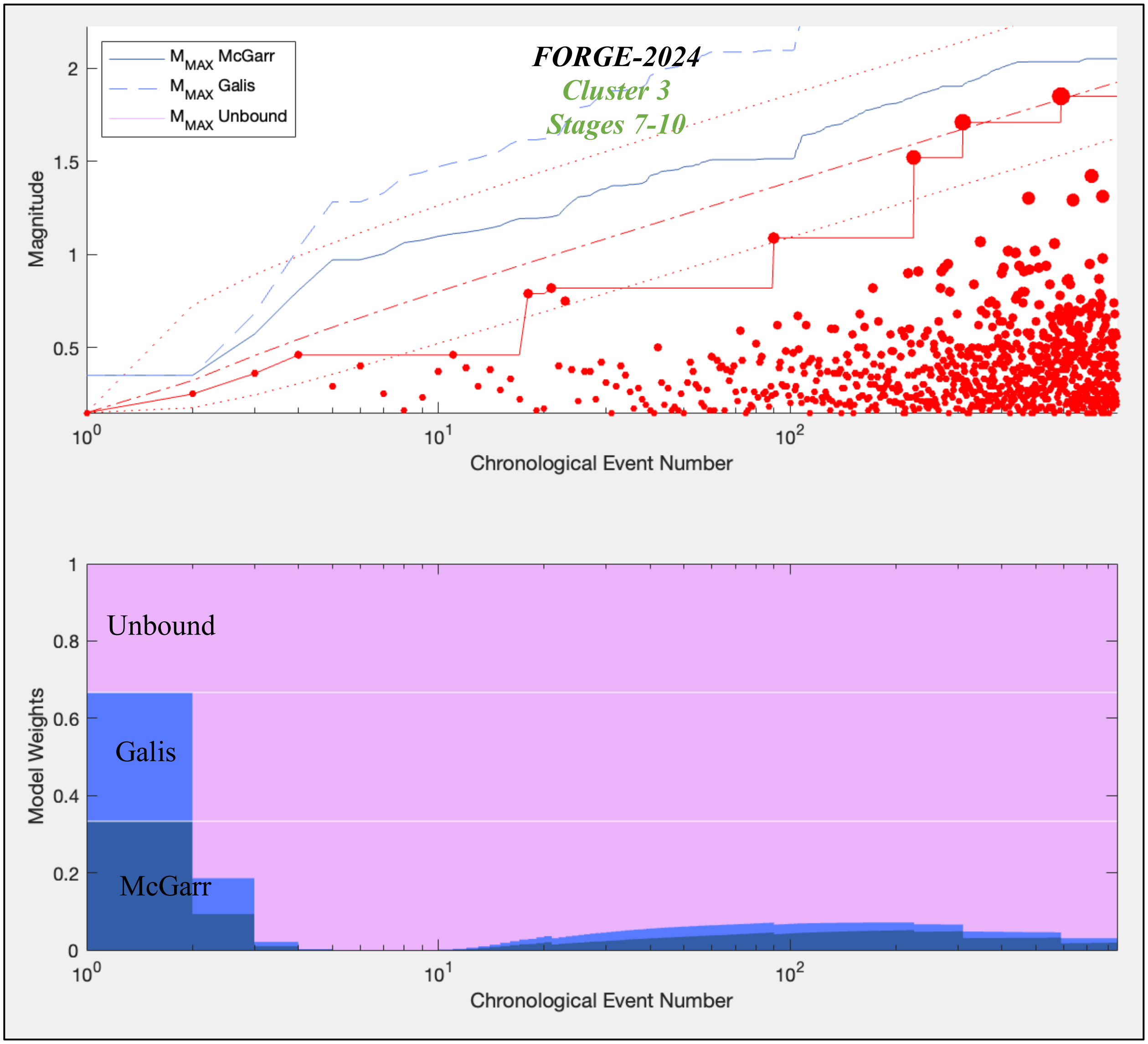

Figure S21. Using the EW-test to discern between M_{MAX} models for cluster 3 at FORGE (2024). In the top panel, the catalogue of earthquake magnitudes (red circles), the observed M_{LRG} sequence (red lines & circles), and expected M_{LRG} at the 10/50/90 percentiles (red dashed lines) are tested using three M_{MAX} assumptions (blue lines). In the bottom panel, AIC/BIC-based ensemble model weights (coloured bars) using all data prior to each new event are shown.

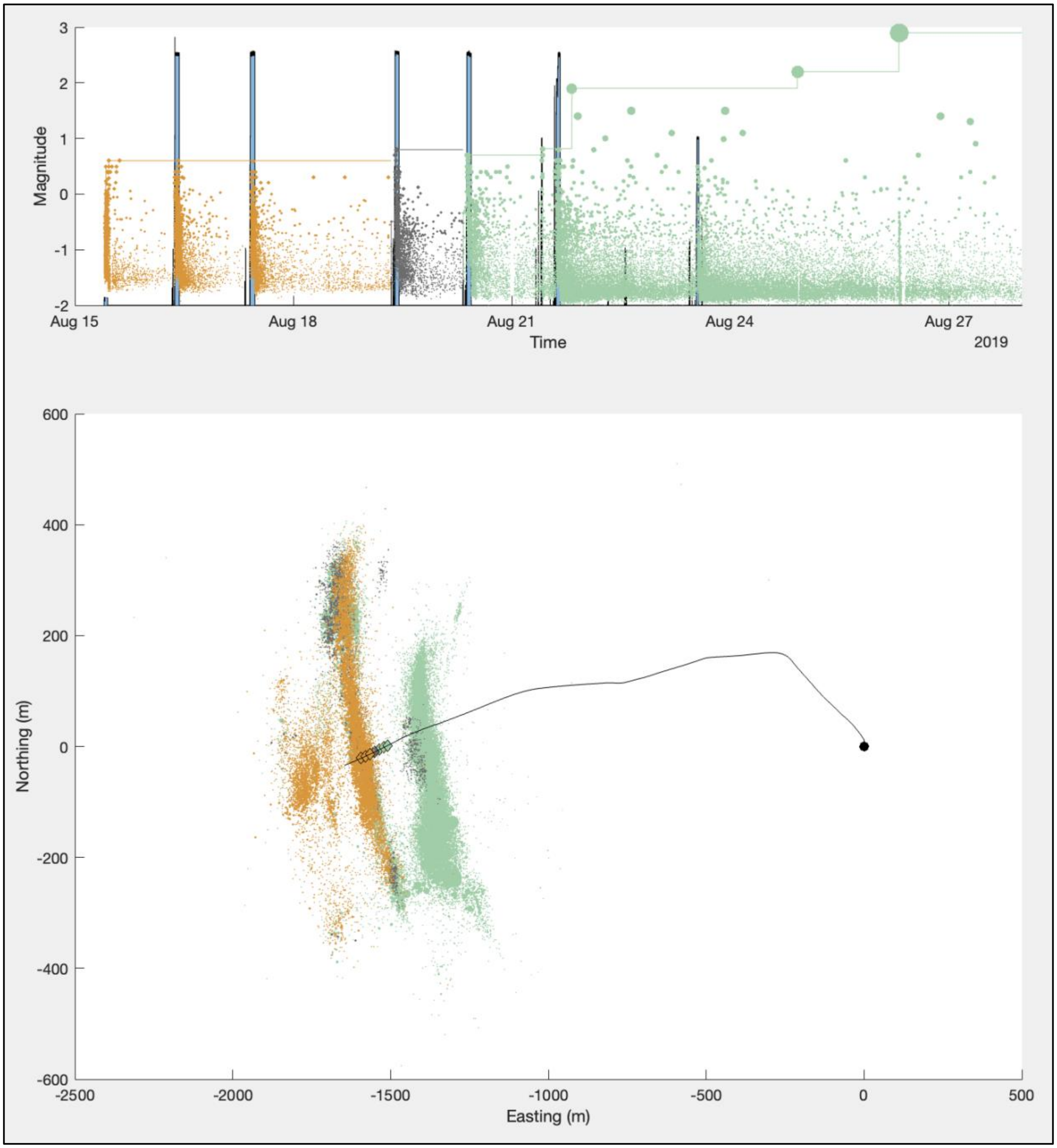

Figure S22. Spatiotemporal clustering of earthquakes during the PNR-2 stimulation. (top panels) The timing of stage stimulations (blue area) against the timings/magnitudes of induced earthquakes (circles) and the sequence of M_{LRG} (coloured line), for each cluster. (bottom panel) The map locations of stage stimulations (diamonds) along the well bore (black line). In all plots, earthquakes are colour coordinated with their respective clusters.

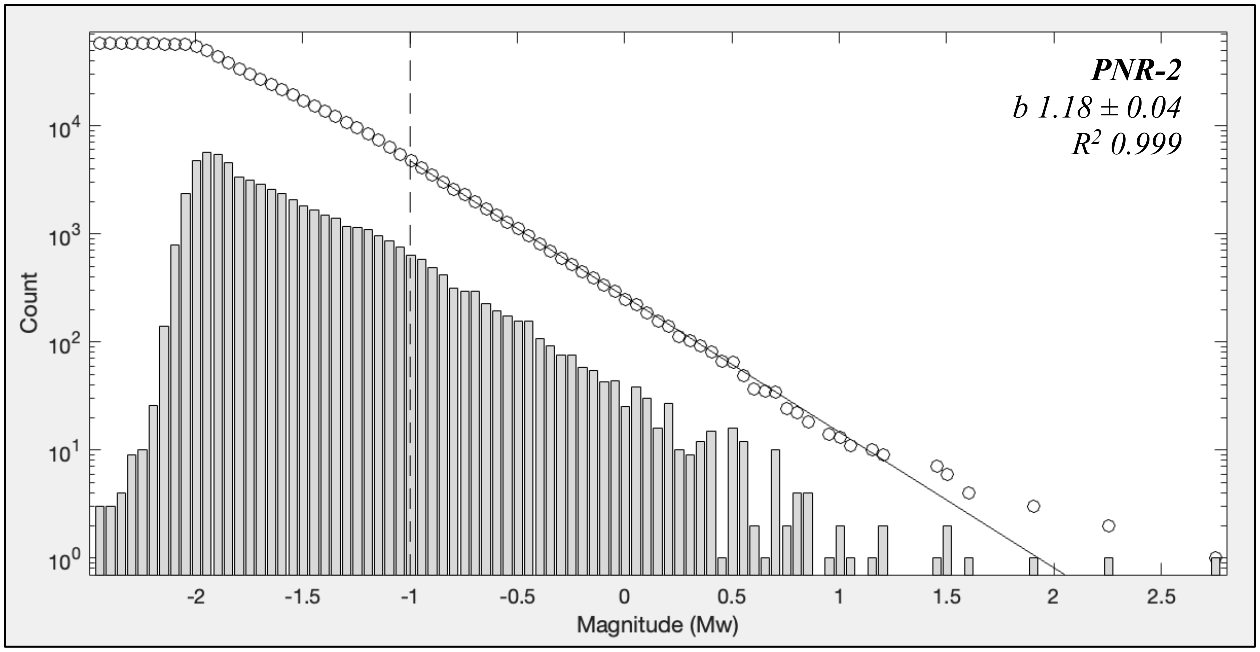

Figure S23. Magnitude statistics at PNR-2 (full). Magnitude frequency distribution of events in the regional catalogue (grey): data counts are plotted as both the cumulative (circles) and non-cumulative (bars) distributions, alongside the best fit to the data (solid line) and the magnitude-of-completeness Mc (dashed line). Data is from the full catalogue at PNR-2.

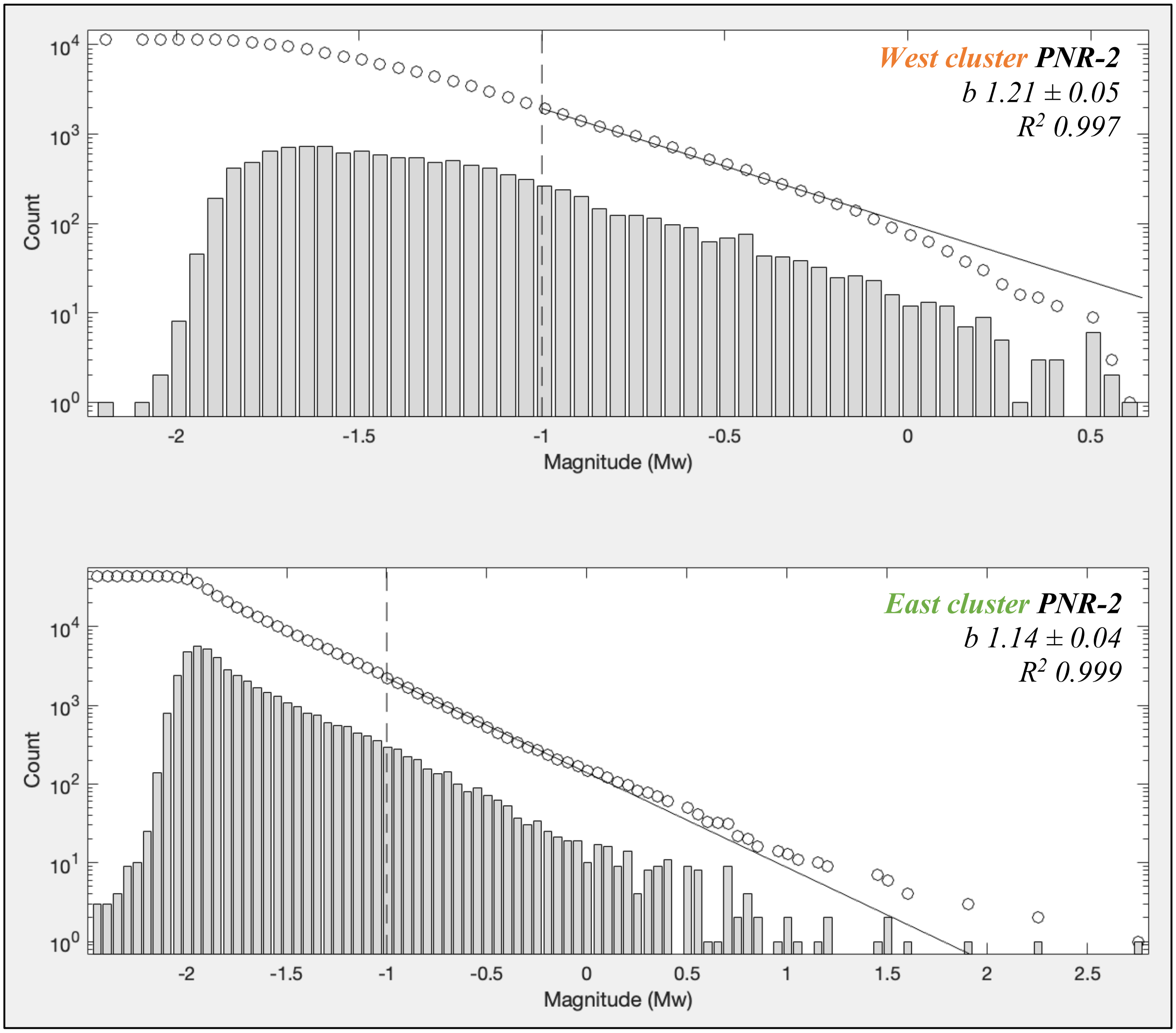

Figure S24. Magnitude statistics at PNR-2 (clustered). Magnitude frequency distribution of events in the regional catalogue (grey): data counts are plotted as both the cumulative (circles) and non-cumulative (bars) distributions, alongside the best fit to the data (solid line) and the magnitude-of-completeness Mc (dashed lines). Data is from the clustered catalogue at PNR-2, for the western-most cluster (stages 1-3; top panel) and the eastern-most cluster (stages 5-7; bottom panel) stimulations.

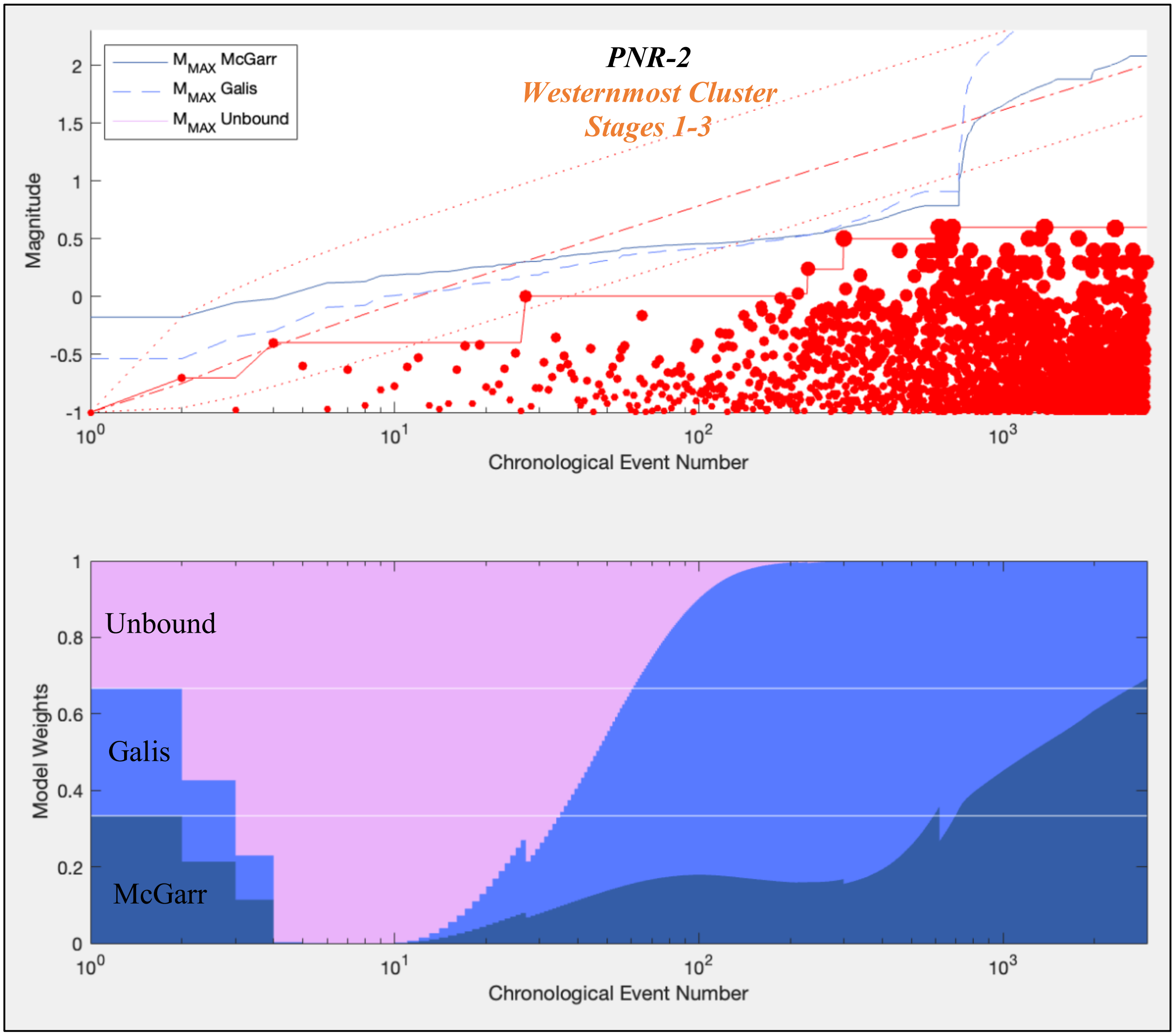

Figure S25. Using the EW-test to discern between M_{MAX} models for the western-most cluster at PNR-2. In the top panel, the catalogue of earthquake magnitudes (red circles), the observed M_{LRG} sequence (red lines & circles), and expected M_{LRG} at the 10/50/90 percentiles (red dashed lines) are tested using three M_{MAX} assumptions (blue lines). In the bottom panel, AIC/BIC-based ensemble model weights (coloured bars) using all data prior to each new event are shown.

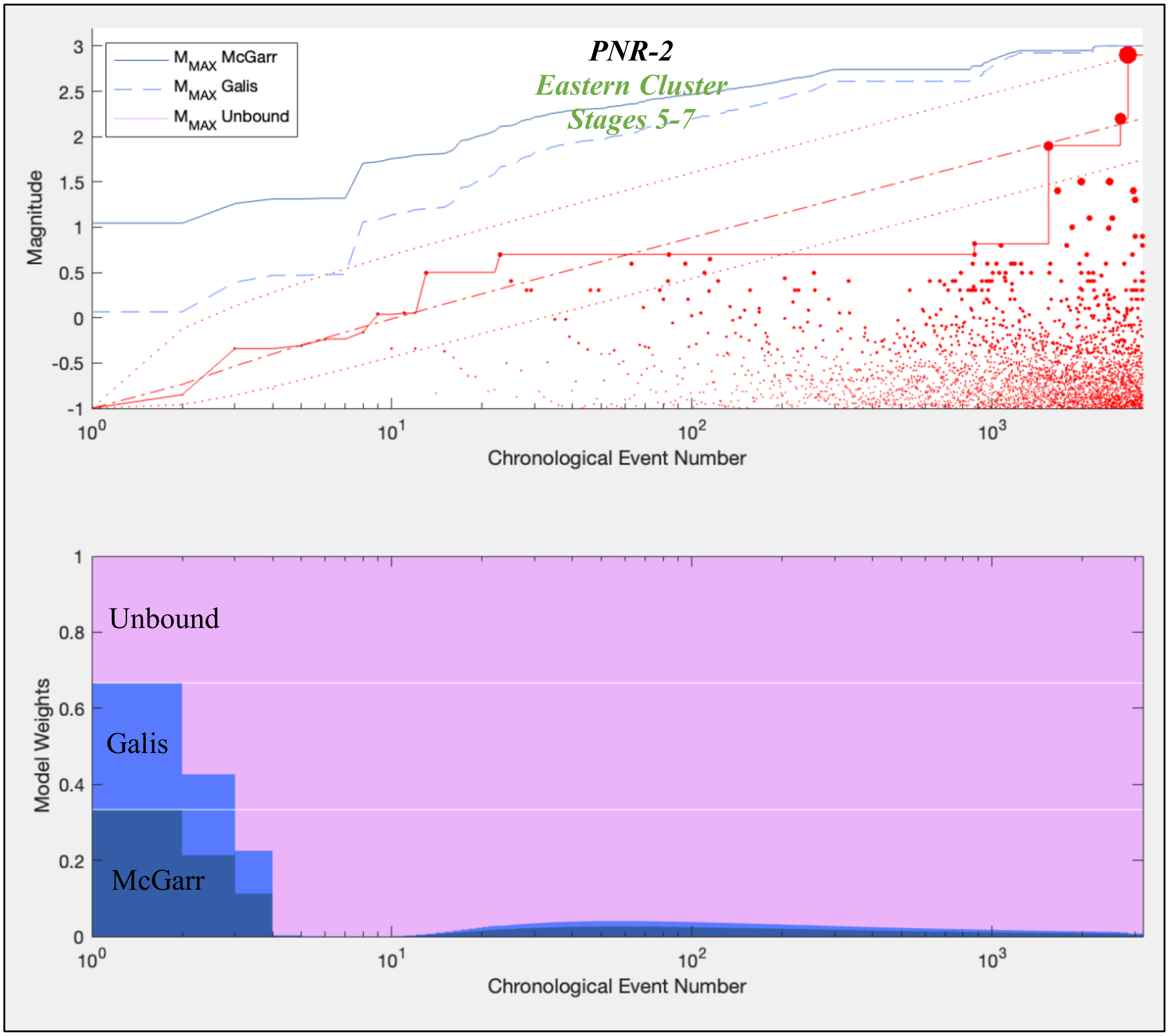

Figure S26. Using the EW-test to discern between M_{MAX} models for the western-most cluster at PNR-2. In the top panel, the catalogue of earthquake magnitudes (red circles), the observed M_{LRG} sequence (red lines & circles), and expected M_{LRG} at the 10/50/90 percentiles (red dashed lines) are tested using three M_{MAX} assumptions (blue lines). In the bottom panel, AIC/BIC-based ensemble model weights (coloured bars) using all data prior to each new event are shown.

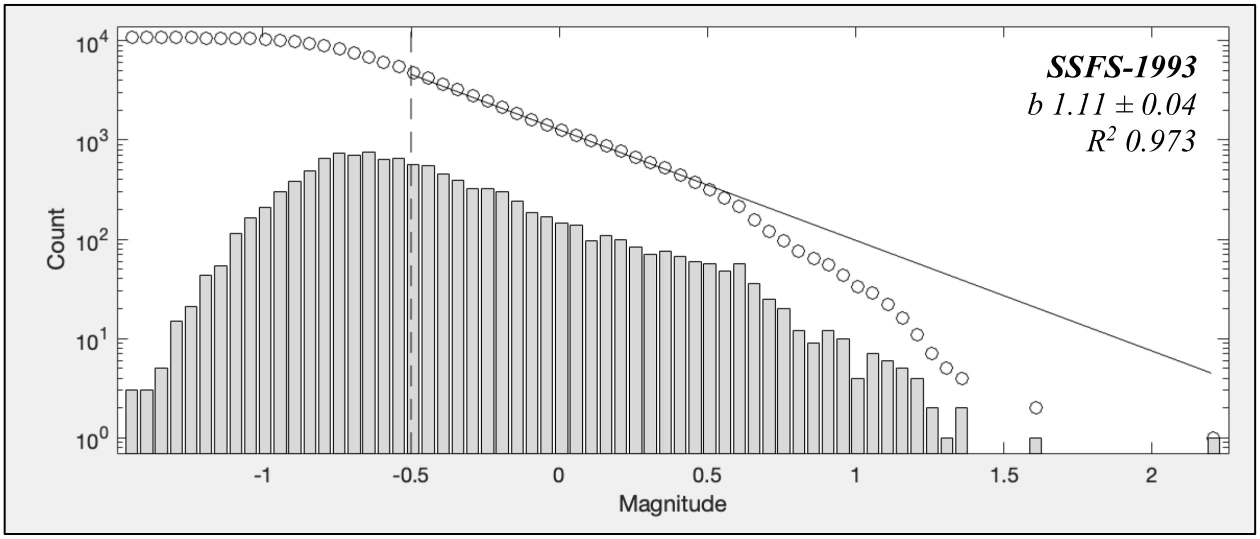

Figure S27. Magnitude statistics at Soultz-sous-Forêts 1993 (full). Magnitude frequency distribution of events in the regional catalogue (grey): data counts are plotted as both the cumulative (circles) and non-cumulative (bars) distributions, alongside the best fit to the data (solid line) and the magnitude-of-completeness Mc (dashed line). Data is from the full catalogue at Soultz-sous-Forêts 1993.

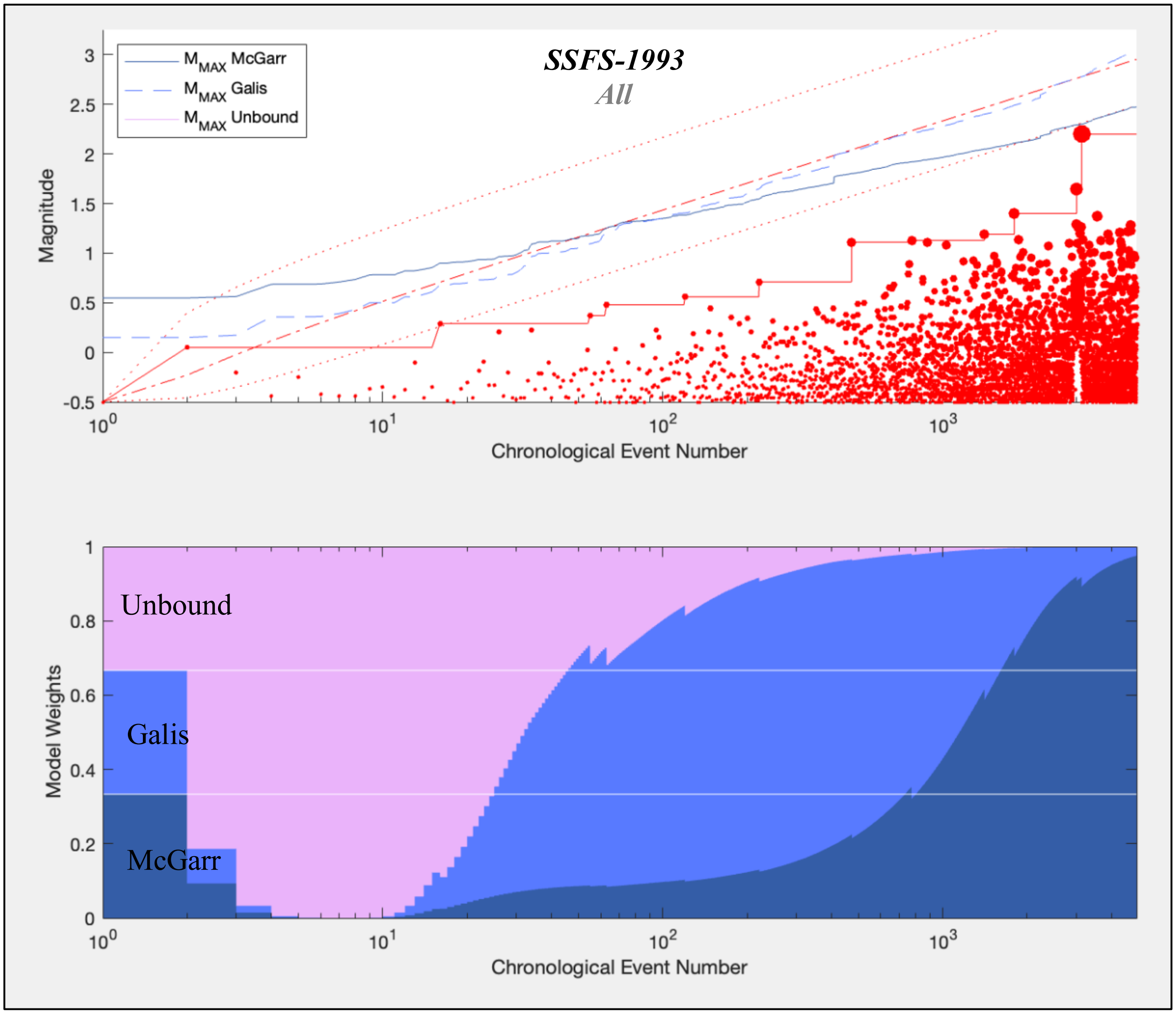

Figure S28. Using the EW-test to discern between M_{MAX} models for all stages at Soultz-sous-Forêts 1993. In the top panel, the catalogue of earthquake magnitudes (red circles), the observed M_{LRG} sequence (red lines & circles), and expected M_{LRG} at the 10/50/90 percentiles (red dashed lines) are tested using three M_{MAX} assumptions (blue lines). In the bottom panel, AIC/BIC-based ensemble model weights (coloured bars) using all data prior to each new event are shown.

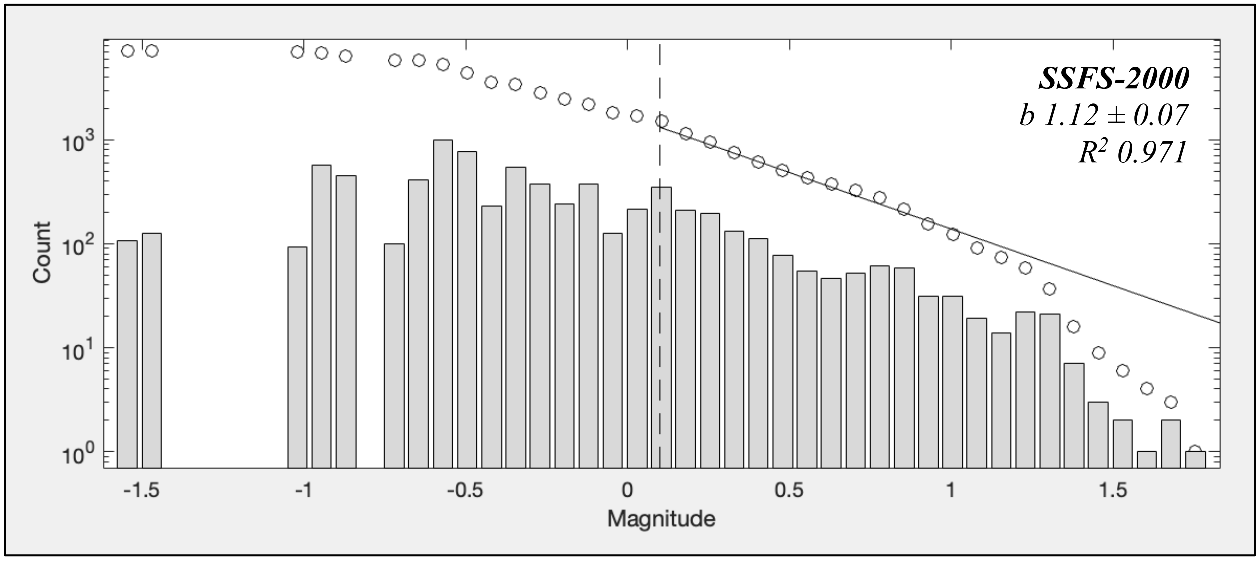

Figure S29. Magnitude statistics at Soultz-sous-Forêts 2000 (full). Magnitude frequency distribution of events in the regional catalogue (grey): data counts are plotted as both the cumulative (circles) and non-cumulative (bars) distributions, alongside the best fit to the data (solid line) and the magnitude-of-completeness Mc (dashed line). Data is from the full catalogue at Soultz-sous-Forêts 2000.

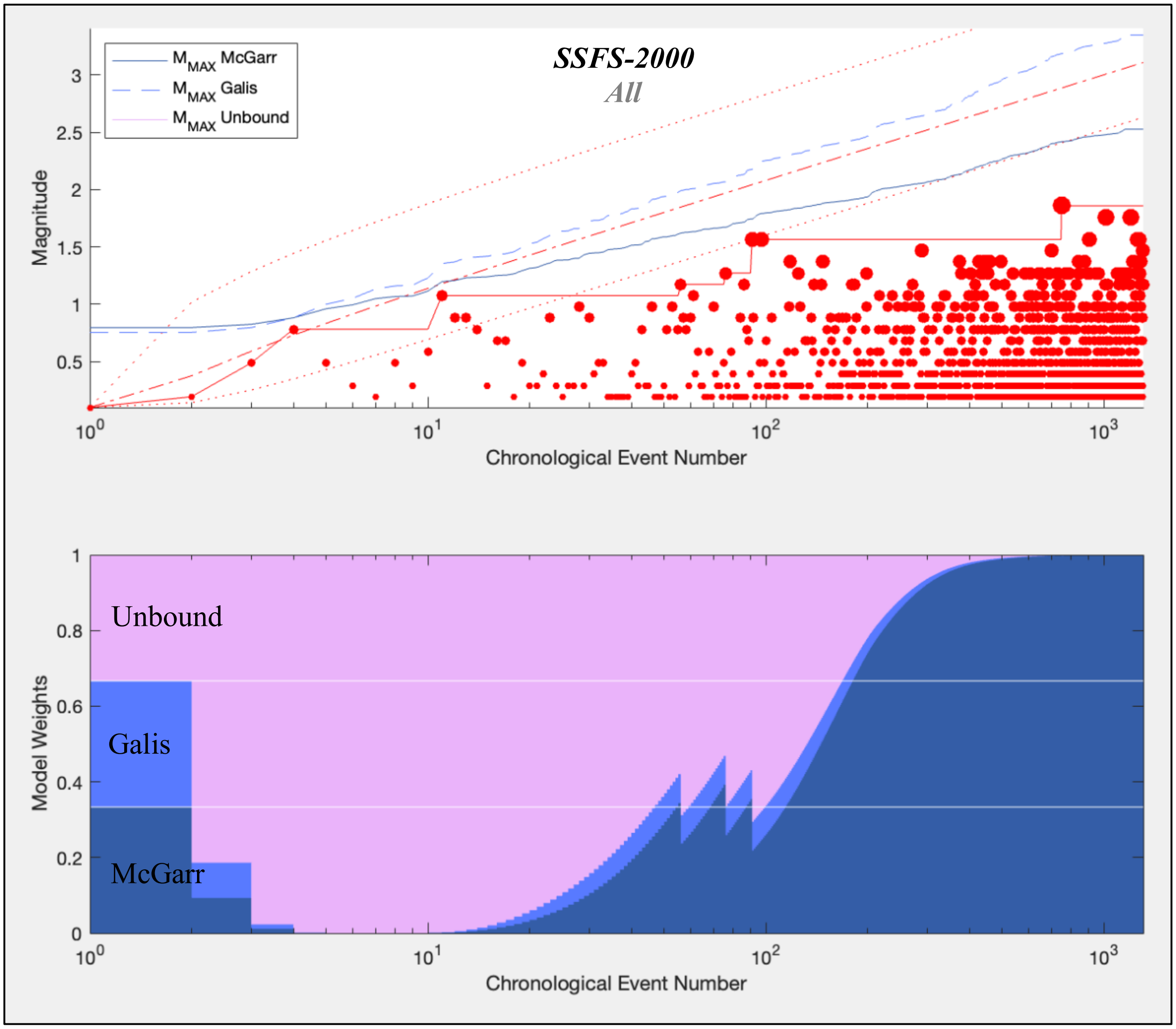

Figure S30. Using the EW-test to discern between MMAX models for all stages at Soultz-sous-Forêts 2000. In the top panel, the catalogue of earthquake magnitudes (red circles), the observed MLRG sequence (red lines & circles), and expected MLRG at the 10/50/90 percentiles (red dashed lines) are tested using three MMAX assumptions (blue lines). In the bottom panel, AIC/BIC-based ensemble model weights (coloured bars) using all data prior to each new event are shown.

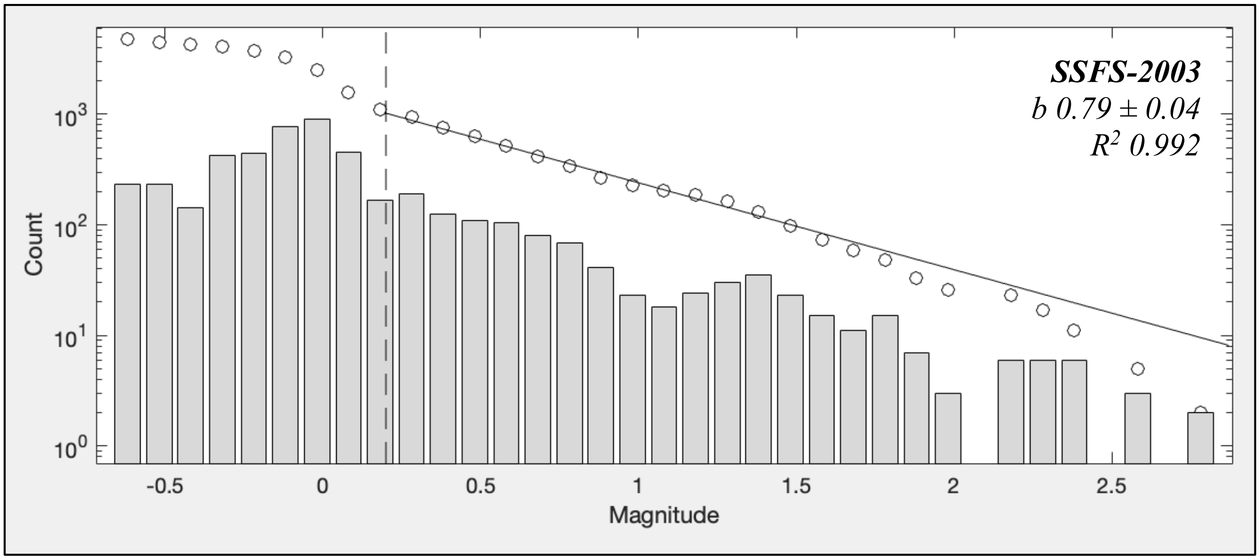

Figure S31. Magnitude statistics at Soultz-sous-Forêts 2003 (full). Magnitude frequency distribution of events in the regional catalogue (grey): data counts are plotted as both the cumulative (circles) and non-cumulative (bars) distributions, alongside the best fit to the data (solid line) and the magnitude-of-completeness Mc (dashed line). Data is from the full catalogue at Soultz-sous-Forêts 2003.

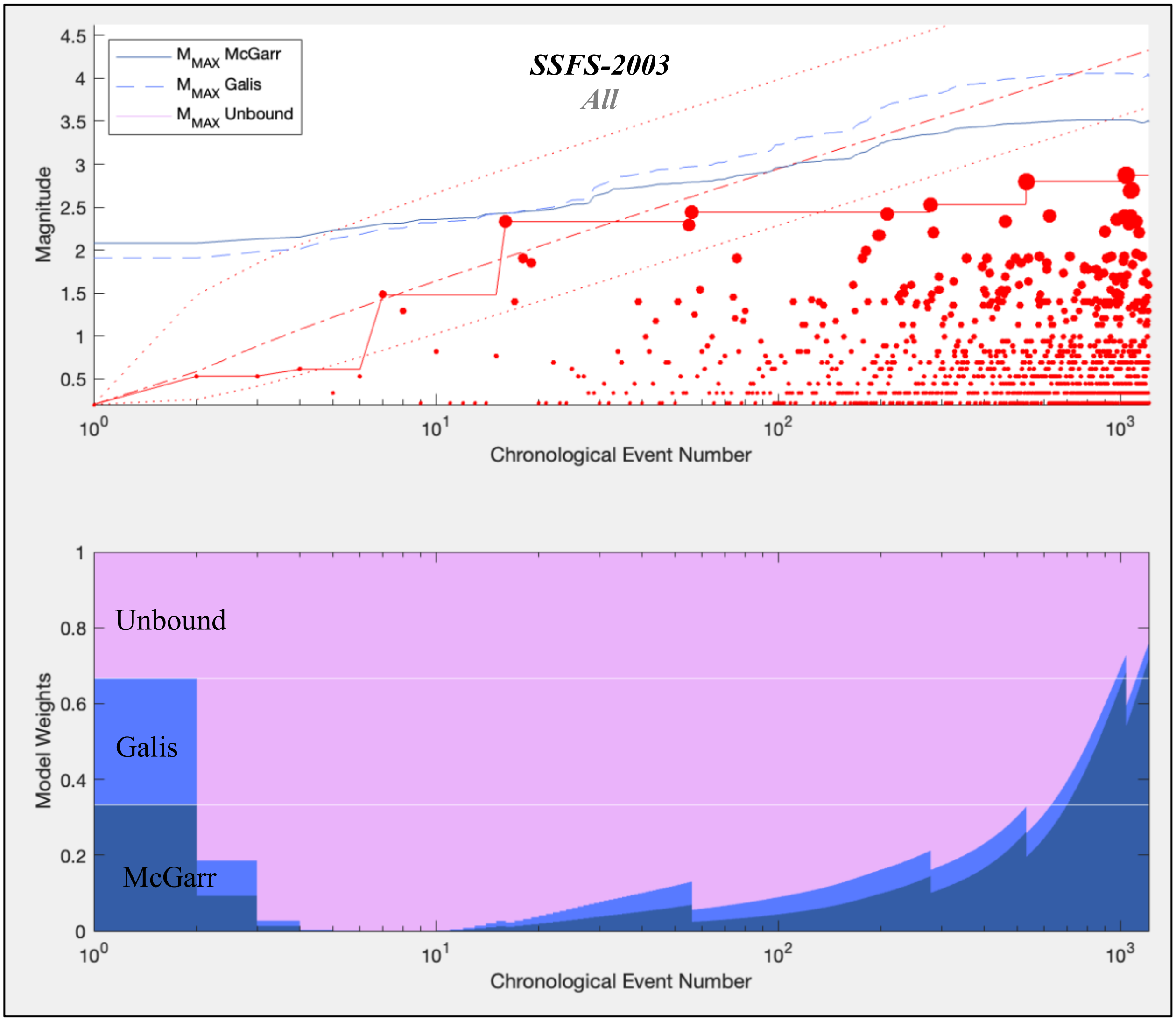

Figure S32. Using the EW-test to discern between M_{MAX} models for all stages at Soultz-sous-Forêts 2003. In the top panel, the catalogue of earthquake magnitudes (red circles), the observed M_{LRG} sequence (red lines & circles), and expected M_{LRG} at the 10/50/90 percentiles (red dashed lines) are tested using three M_{MAX} assumptions (blue lines). In the bottom panel, AIC/BIC-based ensemble model weights (coloured bars) using all data prior to each new event are shown.

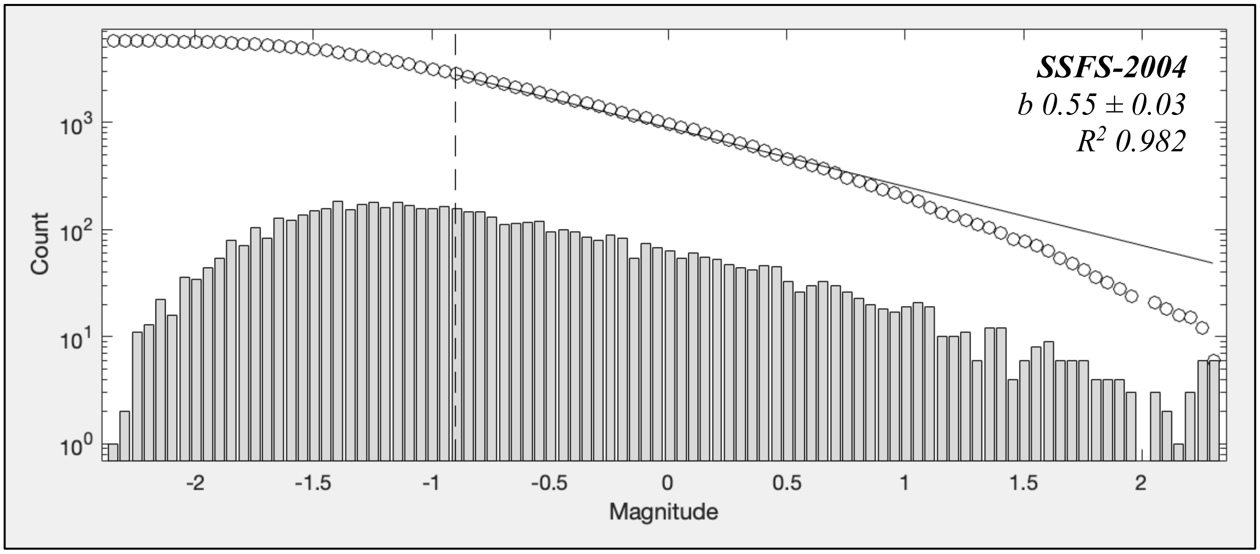

Figure S33. Magnitude statistics at Soultz-sous-Forêts 2004 (full). Magnitude frequency distribution of events in the regional catalogue (grey): data counts are plotted as both the cumulative (circles) and non-cumulative (bars) distributions, alongside the best fit to the data (solid line) and the magnitude-of-completeness Mc (dashed line). Data is from the full catalogue at Soultz-sous-Forêts 2004.

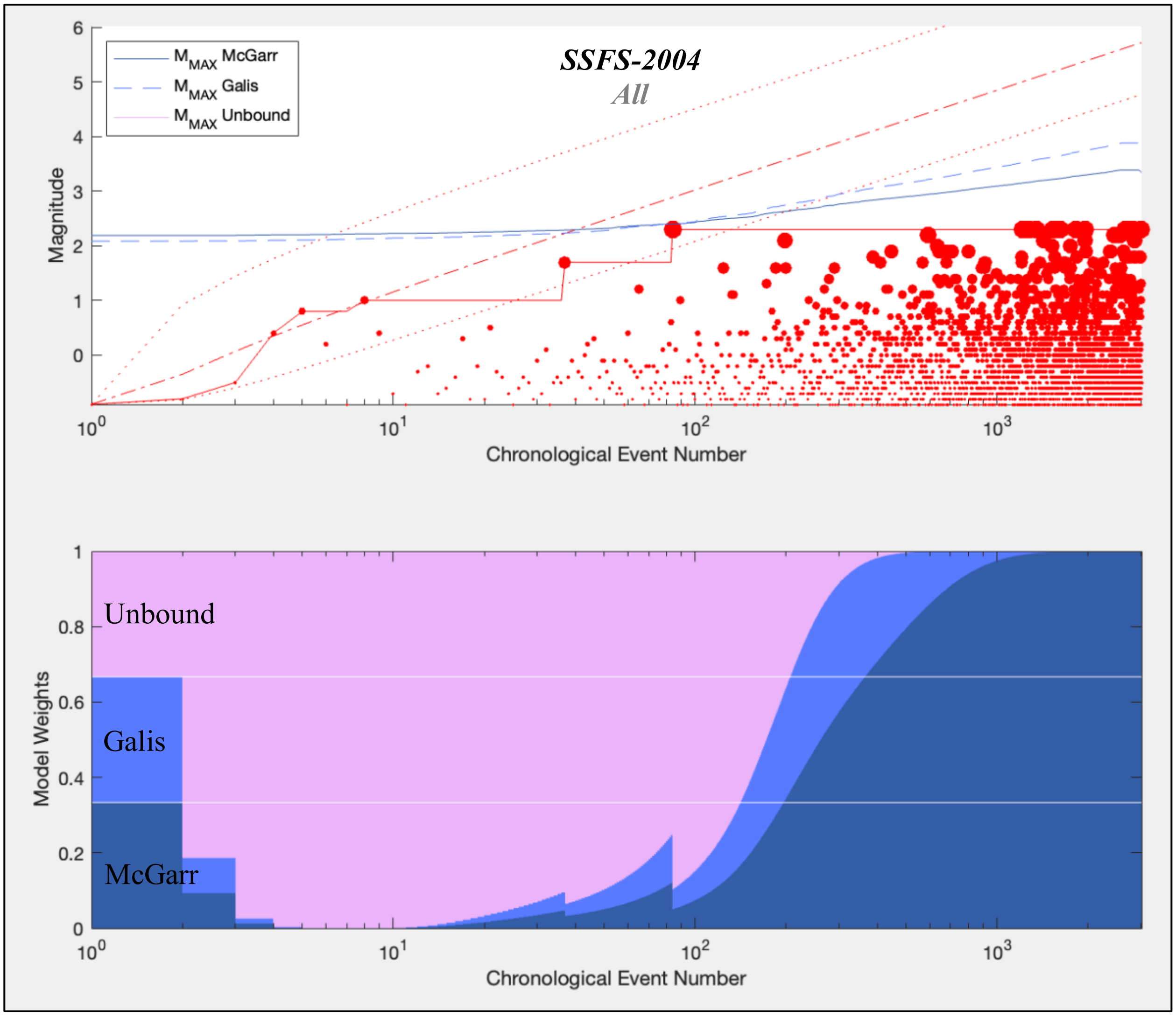

Figure S34. Using the EW-test to discern between M_{MAX} models for all stages at Soultz-sous-Forêts 2004. In the top panel, the catalogue of earthquake magnitudes (red circles), the observed M_{LRG} sequence (red lines & circles), and expected M_{LRG} at the 10/50/90 percentiles (red dashed lines) are tested using three M_{MAX} assumptions (blue lines). In the bottom panel, AIC/BIC-based ensemble model weights (coloured bars) using all data prior to each new event are shown.

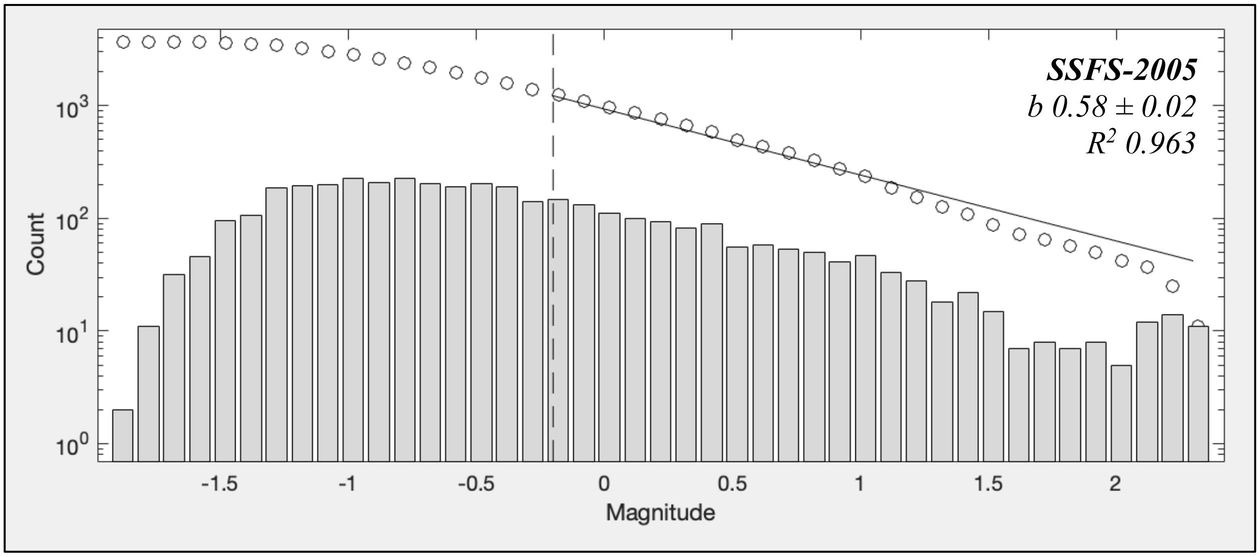

Figure S35. Magnitude statistics at Soultz-sous-Forêts 2005 (full). Magnitude frequency distribution of events in the regional catalogue (grey): data counts are plotted as both the cumulative (circles) and non-cumulative (bars) distributions, alongside the best fit to the data (solid line) and the magnitude-of-completeness Mc (dashed line). Data is from the full catalogue at Soultz-sous-Forêts 2005.

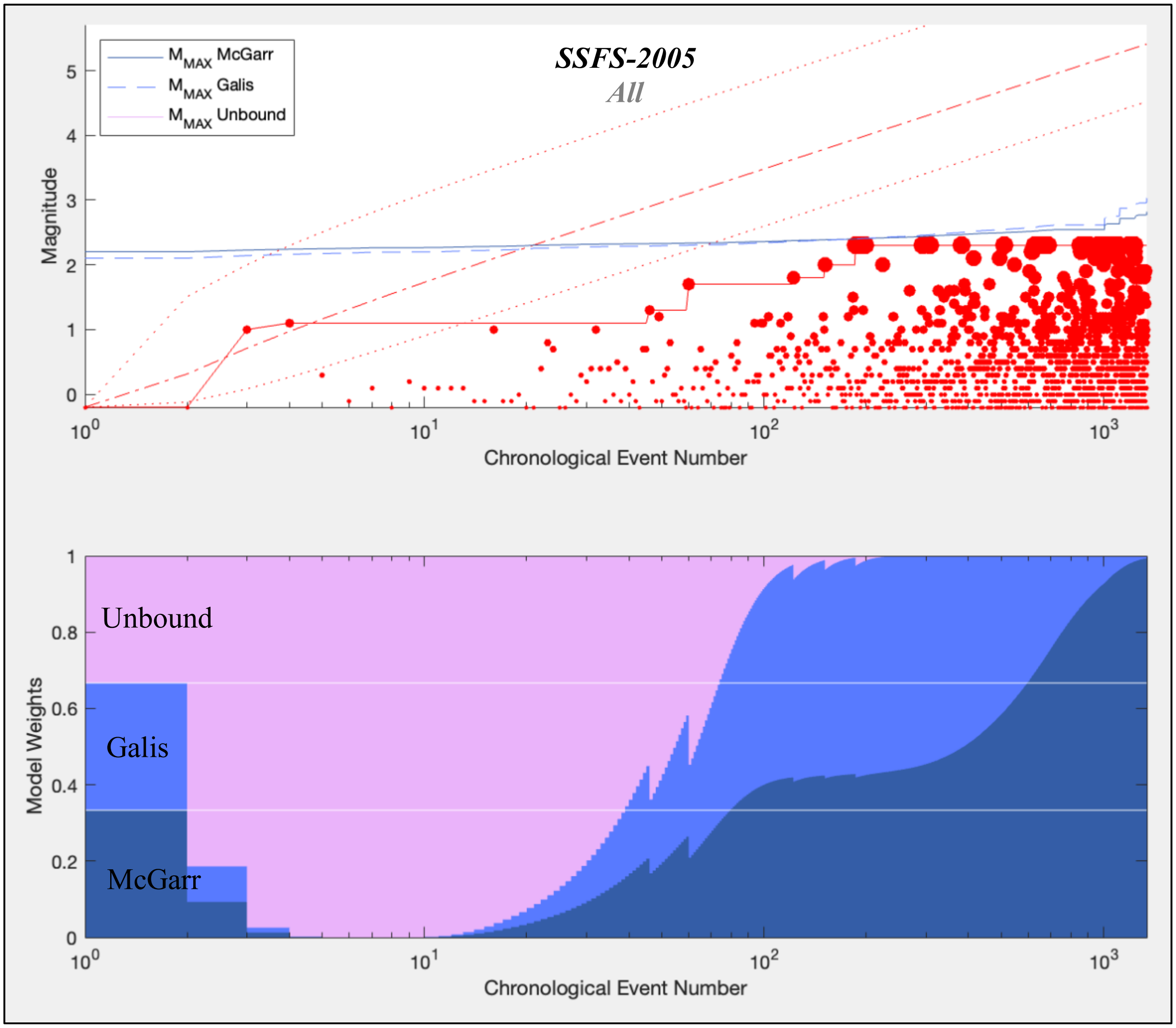

Figure S36. Using the EW-test to discern between M_{MAX} models for all stages at Soultz-sous-Forêts 2005. In the top panel, the catalogue of earthquake magnitudes (red circles), the observed M_{LRG} sequence (red lines & circles), and expected M_{LRG} at the 10/50/90 percentiles (red dashed lines) are tested using three M_{MAX} assumptions (blue lines). In the bottom panel, AIC/BIC-based ensemble model weights (coloured bars) using all data prior to each new event are shown.

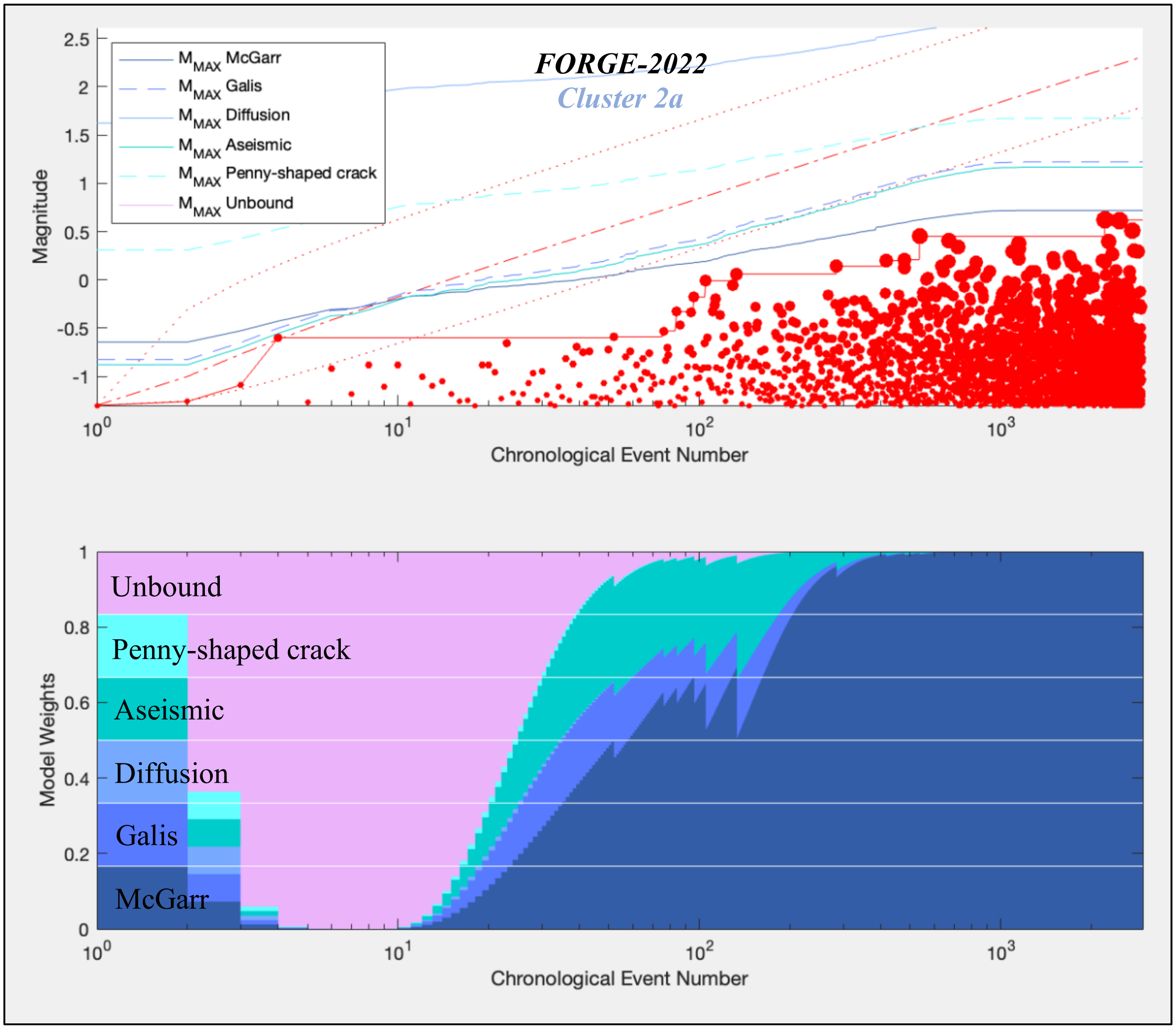

Figure S37. Using the EW-test to discern between additional M_{MAX} models for cluster 2a, stage 3 (2022) at FORGE. In the top panel, the catalogue of earthquake magnitudes (red circles), the observed M_{LRG} sequence (red lines & circles), and expected M_{LRG} at the 10/50/90 percentiles (red dashed lines) are tested using three M_{MAX} assumptions (blue lines). In the bottom panel, AIC/BIC-based ensemble model weights (coloured bars) using all data prior to each new event are shown.

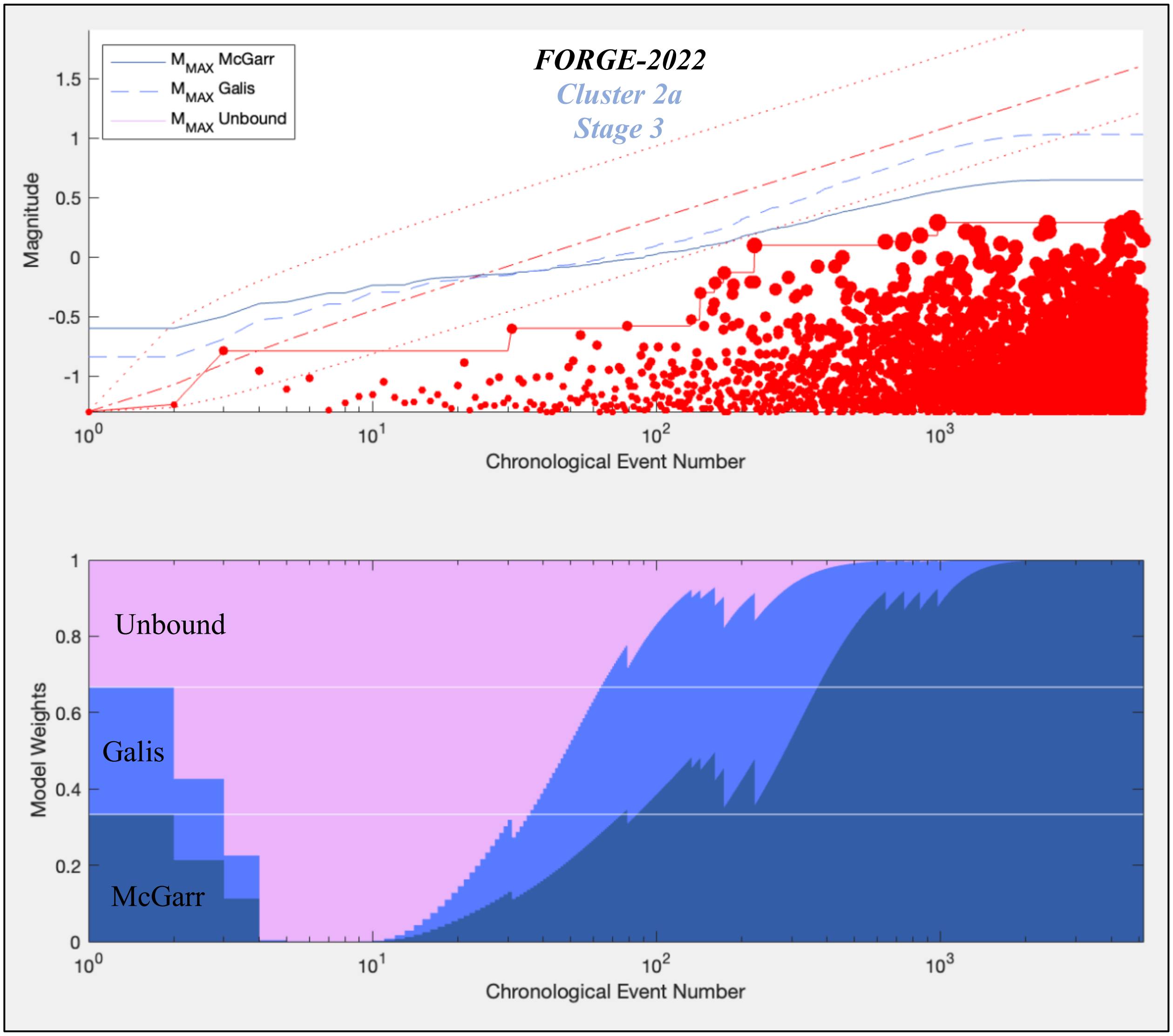

Figure S38. Using the EW-test to discern between M_{MAX} models for cluster 2a (2022) at FORGE, with an alternative catalogue. In the top panel, the catalogue of earthquake magnitudes (red circles), the observed M_{LRG} sequence (red lines & circles), and expected M_{LRG} at the 10/50/90 percentiles (red dashed lines) are tested using three M_{MAX} assumptions (blue lines). In the bottom panel, AIC/BIC-based ensemble model weights (coloured bars) using all data prior to each new event are shown. This plot is the same as Figure S19, just using a different catalogue.

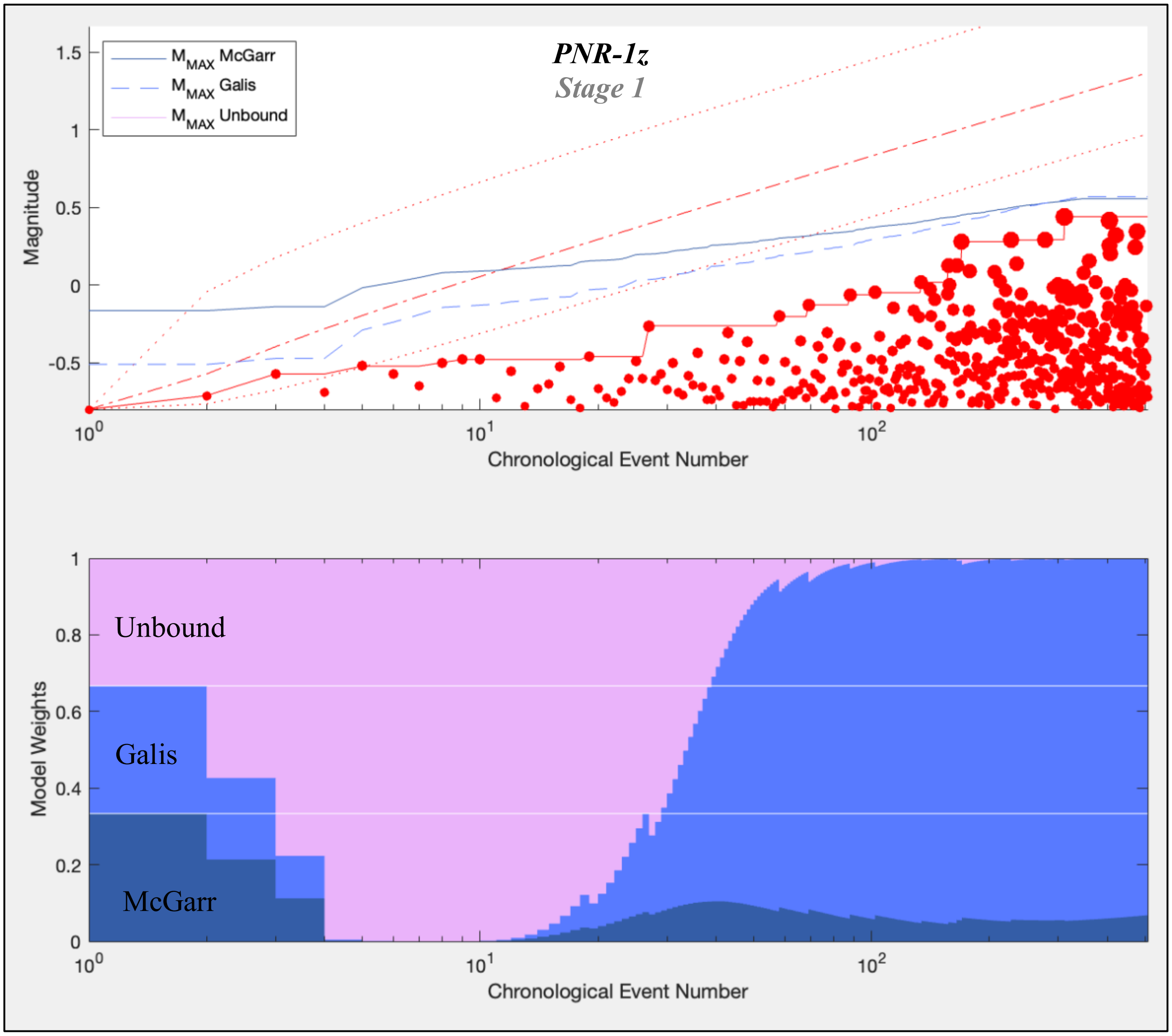

Figure S39. Using the EW-test to discern between M_{MAX} models, for a subset of PNR-1z stages. In the top panel, the catalogue of earthquake magnitudes (red circles), the observed M_{LRG} sequence (red lines & circles), and expected M_{LRG} at the 10/50/90 percentiles (red dashed lines) are tested using three M_{MAX} assumptions (blue lines). In the bottom panel, AIC/BIC-based ensemble model weights (coloured bars) using all data prior to each new event are shown.

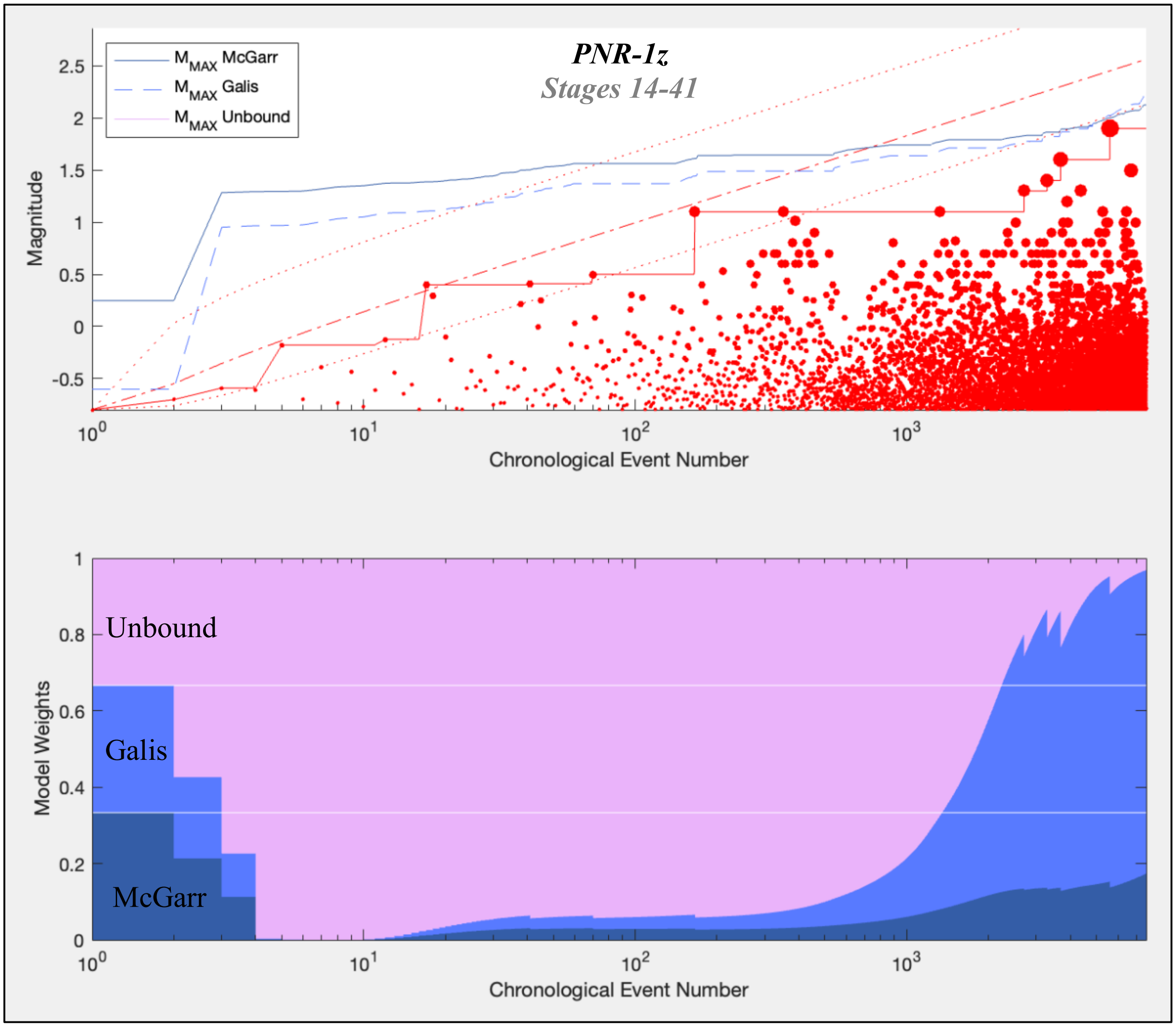

Figure S40. Using the EW-test to discern between MMAX models, for a subset of PNR-1z stages. In the top panel, the catalogue of earthquake magnitudes (red circles), the observed MLRG sequence (red lines & circles), and expected MLRG at the 10/50/90 percentiles (red dashed lines) are tested using three MMAX assumptions (blue lines). In the bottom panel, AIC/BIC-based ensemble model weights (coloured bars) using all data prior to each new event are shown.

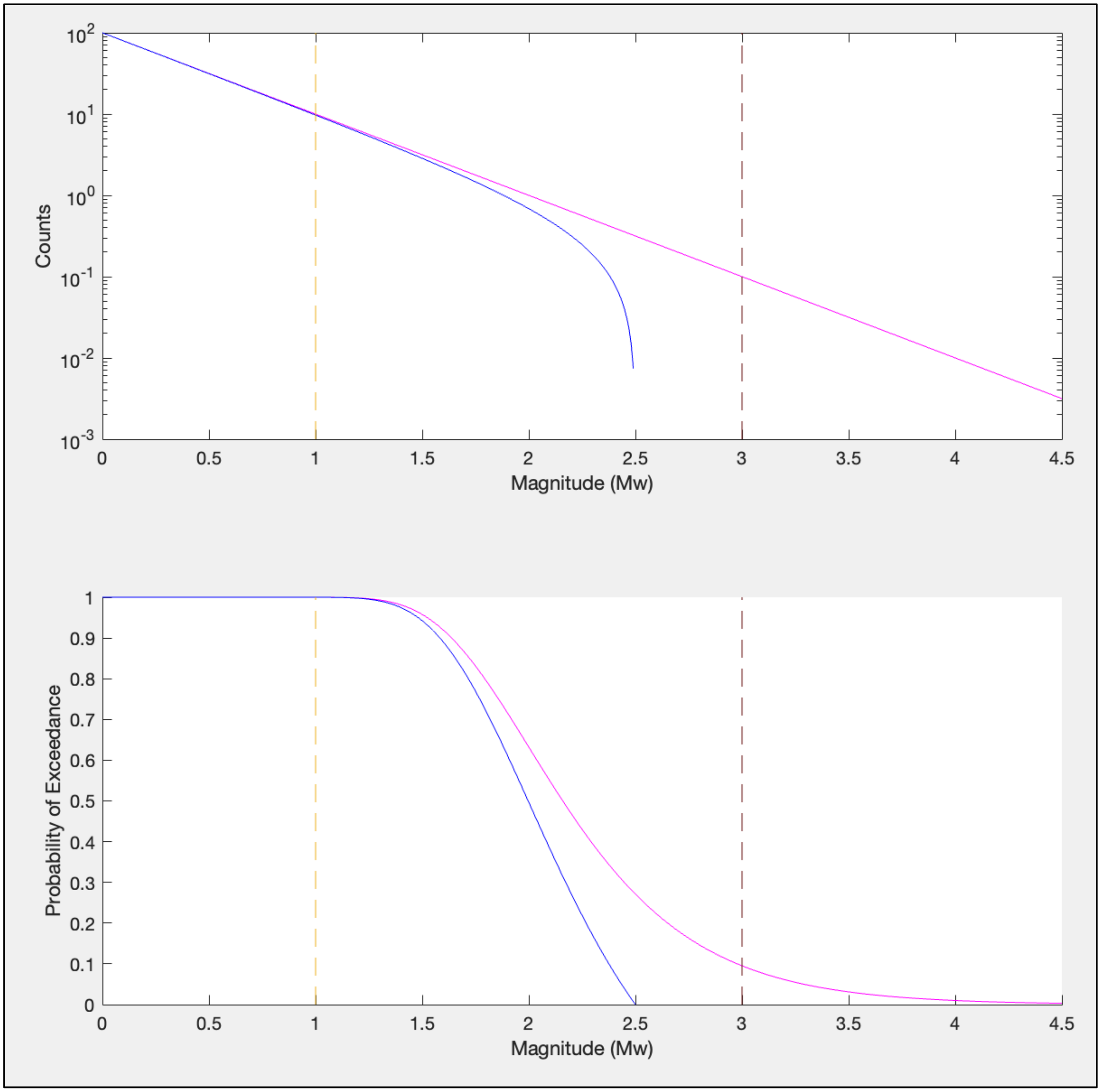

Figure S41. Differences in expected magnitudes for bound and unbound cases. The cumulative GR-FMD counts for the bound (blue line) and unbound (magenta line) cases are shown in the top panel. In the bottom panel, the probability of exceedance of an earthquake magnitude is shown for the bound (blue line) and unbound (magenta line) cases, given the GR-MFD data above. The hypothetical traffic light protocol thresholds are also shown (red/yellow dashed lines).

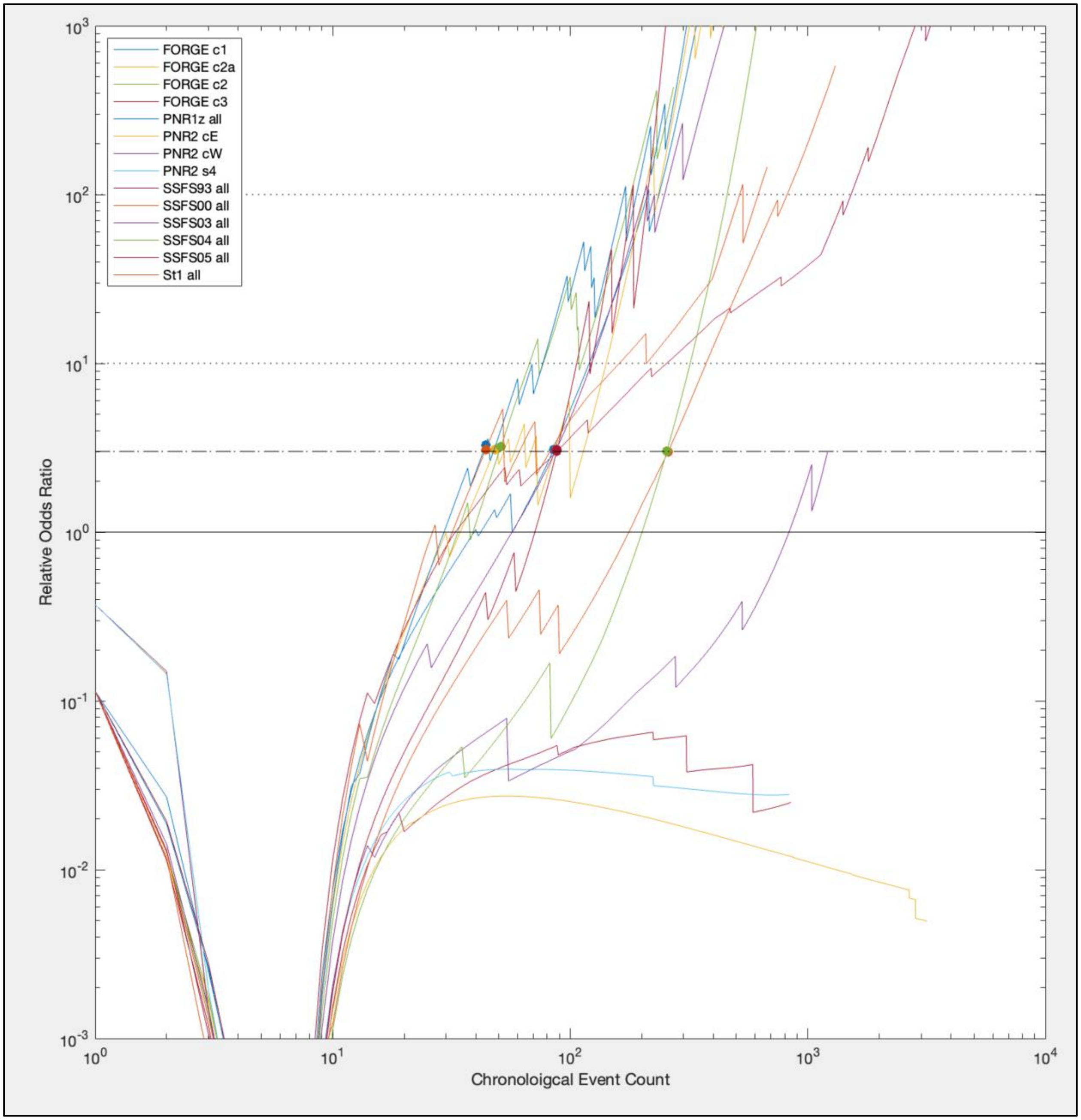

Figure S42. Discerning bound clusters via the EW-test. EW-test odds ratio for each cluster (lines) and the first point (circles) that passes a statistical significance threshold (3-to-1, 10-to-1, 100-to-1; dashed & dotted lines).

Supplementary References

Akaike, H. (1998). Information theory and an extension of the maximum likelihood principle. In *Selected papers of hirotugu akaike* (pp. 199-213). New York, NY: Springer New York.

Berger, V. W., & Zhou, Y. (2014). Kolmogorov–Smirnov test: Overview. *Wiley Statsref: Statistics Reference Online*, https://doi.org/10.1002/9781118445112.stat06558.

Clarke, H., Verdon, J. P., Kettlety, T., Baird, A. F., & Kendall, J. M. (2019). Real-time imaging, forecasting, and management of human-induced seismicity at Preston New Road, Lancashire, England. *Seismological Research Letters*, *90*(5), 1902-1915, https://doi.org/10.1785/0220190110.

Dorbath, L., Cuenot, N., Genter, A., & Frogneux, M. (2009). Seismic response of the fractured and faulted granite of Soultz-sous-Forêts (France) to 5 km deep massive water injections. *Geophysical Journal International*, *177*(2), 653-675, https://doi.org/10.1111/j.1365-246X.2009.04030.x.

Drif, K., Lengliné, O., Kinscher, J., & Schmitbuhl, J. (2024). Induced seismicity controlled by injected hydraulic energy: The case study of the EGS Soultz-sous-Forêts site. *Journal of Geophysical Research: Solid Earth*, *129*(6), e2023JB028190, https://doi.org/10.1029/2023JB028190.

Dyer, B., Karvounis, D., & Bethmann, F. (2023). Microseismic event catalogues from the well 16A(78)-32 stimulation in April, 2022 in Utah FORGE. *ISC Seismological Dataset Repository*. https://doi.org/10.31905/52CC4QZB.

Holschneider, M., Zöller, G., & Hainzl, S. (2011). Estimation of the maximum possible magnitude in the framework of a doubly truncated Gutenberg–Richter model. *Bulletin of the Seismological Society of America*, *101*(4), 1649-1659, https://doi.org/10.1785/0120100289.

Kass, R. E., & Raftery, A. E. (1995). Bayes factors. *Journal of the American Statistical Association*, 90(430), 773-795, https://doi.org/10.2307/2291091.

Galis, M., Ampuero, J. P., Mai, P. M., & Cappa, F. (2017). Induced seismicity provides insight into why earthquake ruptures stop. *Science Advances*, *3*(12), eaap7528, https://doi.org/10.1126/sciadv.aap7528.

Gutenberg, B., & Richter, C. F. (1944). Frequency of earthquakes in California. *Bulletin of the Seismological society of America*, *34*(4), 185-188, https://doi.org/10.1785/BSSA0340040185.

Hallo, M., Oprsal, I., Eisner, L., & Ali, M. Y. (2014). Prediction of magnitude of the largest potentially induced seismic event. *Journal of Seismology*, *18*, 421-431, https://doi.org/10.1007/s10950-014-9417-4.

Hanks, T. C., and H. Kanamori (1979), A moment magnitude scale, *Journal of Geophysical Research*, 84, 2348-2350, https://doi.org/10.1029/JB084iB05p02348.

Kagan, Y. Y. (2002). Seismic moment distribution revisited: I. Statistical results. *Geophysical Journal International*, *148*(3), 520-541, https://doi.org/10.1046/j.1365-246x.2002.01594.x.

Kwiatek, G., Saarno, T., Ader, T., Bluemle, F., Bohnhoff, M., Chendorain, M., ... & Wollin, C. (2019). Controlling fluid-induced seismicity during a 6.1-km-deep geothermal stimulation in Finland. *Science Advances*, *5*(5), eaav7224, https://doi.org/10.1126/sciadv.aav7224.

Leptokaropoulos, K., Cielesta, S., Staszek, M., Olszewska, D., Lizurek, G., Kocot, J., ... & Szepieniec, T. (2019). IS-EPOS: a platform for anthropogenic seismicity research. *Acta Geophysica*, *67*(1), 299-310, https://doi.org/10.1007/s11600-018-0209-z.

Marzocchi, W., & Sandri, L. (2003). A review and new insights on the estimation of the b-value and its uncertainty. *Annals of geophysics*, 46(6), 1271-1282, http://hdl.handle.net/2122/1017.

McGarr, A. (2014). Maximum magnitude earthquakes induced by fluid injection. *Journal of Geophysical Research: solid earth*, *119*(2), 1008-1019, https://doi.org/10.1002/2013JB010597.

McQuarrie, A. D. (1999). A small-sample correction for the Schwarz SIC model selection criterion. *Statistics & Probability Letters*, *44*(1), 79-86, https://doi.org/10.1016/S0167-7152(98)00294-6.

Niemz, P., Pankow, K., Isken, M.P., Whidden, K., McLennan, J., & Moore, J. (2025). Mapping fracture zones with nodal geophone patches: Insights from induced microseismicity during the 2024 stimulations at Utah FORGE. *Seismological Research Letters*, https://doi.org/10.1785/0220240300.

Schultz, R., Atkinson, G., Eaton, D. W., Gu, Y. J., & Kao, H. (2018). Hydraulic fracturing volume is associated with induced earthquake productivity in the Duvernay play. *Science*, *359*(6373), 304-308, https://doi.org/10.1126/science.aao0159.

Schultz, R. (2024). Inferring maximum magnitudes from the ordered sequence of large earthquakes, *Philosophical Transactions of the Royal Society A: Mathematical, Physical and Engineering Sciences*, 38, 20230185, https://doi.org/10.1098/rsta.2023.0185.

Schwarz, G. (1978). Estimating the dimension of a model. *The Annals of Statistics*, 461-464.

Shapiro, S. A., Dinske, C., Langenbruch, C., & Wenzel, F. (2010). Seismogenic index and magnitude probability of earthquakes induced during reservoir fluid stimulations. *The Leading Edge*, *29*(3), 304-309, https://doi.org/10.1190/1.3353727.

Shi, P., Grigoli, F., Lanza, F., Beroza, G. C., Scarabello, L., & Wiemer, S. (2022). MALMI: An automated earthquake detection and location workflow based on machine learning and waveform

migration. *Seismological Research Letters*, *93*(5), 2467-2483, https://doi.org/10.1785/0220220071.

Sugiura, N. (1978). Further analysis of the data by Akaike's information criterion and the finite corrections. *Communications in Statistics-Theory and Methods*, *7*(1), 13-26, https://doi.org/10.1080/03610927808827599.

Wagenmakers, E. J., & Farrell, S. (2004). AIC model selection using Akaike weights. *Psychonomic bulletin & review*, *11*, 192-196, https://doi.org/10.3758/BF03206482.

van der Elst, N. J., Page, M. T., Weiser, D. A., Goebel, T. H., & Hosseini, S. M. (2016). Induced earthquake magnitudes are as large as (statistically) expected. *Journal of Geophysical Research: Solid Earth*, *121*(6), 4575-4590, https://doi.org/10.1002/2016JB012818.

Zhuang, J., Harte, D., Werner, M.J., Hainzl, S., & Zhou, S. (2012). Basic models of seismicity: temporal models, *Community Online Resource for Statistical Seismicity Analysis*, v1.0, p 42, https://doi.org/10.5078/corssa-79905851.
