# Supplementary material for: The bound growth of induced earthquakes could de-risk hydraulic fracturing
Source: Commun Earth Environ. 2025 Nov 22;6(1):995. doi: 10.1038/s43247-025-02881-2 (PMC12685737; doi:10.1038/s43247-025-02881-2)
Supplement: Supplementary file 3 — Reporting summary [file 43247_2025_2881_MOESM3_ESM.docx]

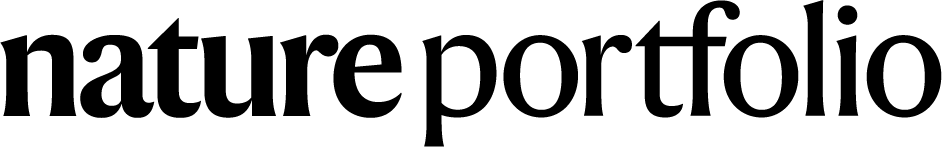
Corresponding author(s): Ryan Schultz

Last updated by author(s): 2025 09 24

Reporting Summary

Nature Portfolio wishes to improve the reproducibility of the work that we publish. This form provides structure for consistency and transparency in reporting. For further information on Nature Portfolio policies, see our Editorial Policies and the Editorial Policy Checklist.

Please do not complete any field with "not applicable" or n/a. Refer to the help text for what text to use if an item is not relevant to your study. For final submission: please carefully check your responses for accuracy; you will not be able to make changes later.

## Statistics

For all statistical analyses, confirm that the following items are present in the figure legend, table legend, main text, or Methods section.

n/a


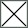

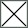

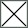


Confirmed


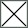
 The exact sample size (*n*) for each experimental group/condition, given as a discrete number and unit of measurement


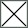
 A statement on whether measurements were taken from distinct samples or whether the same sample was measured repeatedly The statistical test(s) used AND whether they are one- or two-sided

*Only common tests should be described solely by name; describe more complex techniques in the Methods section.*


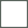
 A description of all covariates tested


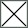
 A description of any assumptions or corrections, such as tests of normality and adjustment for multiple comparisons

A full description of the statistical parameters including central tendency (e.g. means) or other basic estimates (e.g. regression coefficient) AND variation (e.g. standard deviation) or associated estimates of uncertainty (e.g. confidence intervals)

For null hypothesis testing, the test statistic (e.g. *F*, *t*, *r*) with confidence intervals, effect sizes, degrees of freedom and *P* value noted

1

nature portfolio | reporting summary

*April 2023*

*Give P values as exact values whenever suitable.*

For Bayesian analysis, information on the choice of priors and Markov chain Monte Carlo settings

For hierarchical and complex designs, identification of the appropriate level for tests and full reporting of outcomes Estimates of effect sizes (e.g. Cohen's *d*, Pearson's *r*), indicating how they were calculated

*Our web collection on statistics for biologists contains articles on many of the points above.*

## Software and code

All codes to perform the analysis (and recreate figures/results) are custom Matlab routines. They are provided online in a GitHub repository.

Policy information about availability of computer code Data collection

Data analysis

See above.

For manuscripts utilizing custom algorithms or software that are central to the research but not yet described in published literature, software must be made available to editors and reviewers. We strongly encourage code deposition in a community repository (e.g. GitHub). See the Nature Portfolio guidelines for submitting code & software for further information.

## Data

Policy information about availability of data

All manuscripts must include a data availability statement. This statement should provide the following information, where applicable:

- Accession codes, unique identifiers, or web links for publicly available datasets
- A description of any restrictions on data availability
- For clinical datasets or third party data, please ensure that the statement adheres to our policy

See the above point for software and code. This repository also provides the underlying data. The corresponding statement is provided in the manuscript.

2

nature portfolio | reporting summary

*April 2023*

## Research involving human participants, their data, or biological material

Policy information about studies with human participants or human data. See also policy information about sex, gender (identity/presentation), and sexual orientation and race, ethnicity and racism.

N/A

Reporting on sex and gender

Reporting on race, ethnicity, or other socially relevant groupings

N/A

Population characteristics

N/A

Recruitment

N/A

Ethics oversight

N/A

Note that full information on the approval of the study protocol must also be provided in the manuscript.

# Field-specific reporting

Please select the one below that is the best fit for your research. If you are not sure, read the appropriate sections before making your selection.


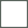
 Life sciences
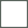
 Behavioural & social sciences
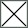
 Ecological, evolutionary & environmental sciences

For a reference copy of the document with all sections, see nature.com/documents/nr-reporting-summary-flat.pdf

# Ecological, evolutionary & environmental sciences study design

All studies must disclose on these points even when the disclosure is negative.

Study description

We used new statistical tests to infer if earthquake sizes were somehow restricted in their growth.

Research sample

Earthquakes recorded during the stimulation/injection at enhanced geothermal systems and hydraulic fracturing operations.

Sampling strategy

N/A

Data collection

Data was collected from online repositories.

Timing and spatial scale

Each operation took on the order of weeks to months for stimulation and earthquakes were within a few kms of the operation.

Data exclusions

N/A

Reproducibility

Both the data and the codes to produce the figures/results have been shared.

Randomization

N/A

Blinding

N/A

Did the study involve field work? Yes No

# Reporting for specific materials, systems and methods

We require information from authors about some types of materials, experimental systems and methods used in many studies. Here, indicate whether each material, system or method listed is relevant to your study. If you are not sure if a list item applies to your research, read the appropriate section before selecting a response.

3

nature portfolio | reporting summary

*April 2023*

Materials & experimental systems Methods


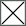

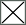

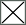

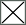

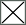

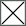

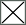


n/a Involved in the study Antibodies Eukaryotic cell lines

Palaeontology and archaeology Animals and other organisms Clinical data

Dual use research of concern

Plants


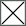

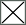

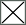


n/a Involved in the study

ChIP-seq

Flow cytometry

MRI-based neuroimaging

## Plants

Seed stocks

N/A

Novel plant genotypes

*Describe the methods by which all novel plant genotypes were produced. This includes those generated by transgenic approaches, gene editing, chemical/radiation-based mutagenesis and hybridization. For transgenic lines, describe the transformation method, the number of independent lines analyzed and the generation upon which experiments were performed. For gene-edited lines, describe the editor used, the endogenous sequence targeted for editing, the targeting guide RNA sequence (if applicable) and how the editor was applied.*

*Describe any authentication procedures for each seed stock used or novel genotype generated. Describe any experiments used to*

*assess the effect of a mutation and, where applicable, how potential secondary effects (e.g. second site T-DNA insertions, mosiacism, off-target gene editing) were examined.*

Authentication


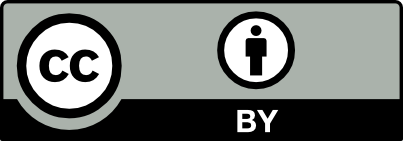
This checklist template is licensed under a Creative Commons Attribution 4.0 International License, which permits use, sharing, adaptation, distribution and reproduction in any medium or format, as long as you give appropriate credit to the original author(s) and the source, provide a link to the Creative Commons license, and indicate if changes were made. The images or other third party material in this article are included in the article's Creative Commons license, unless indicated otherwise in a credit line to the material. If material is not included in the article's Creative Commons license and your intended use is not permitted by statutory regulation or exceeds the permitted use, you will need to obtain permission directly from the copyright holder. To view a copy of this license, visit <http://creativecommons.org/licenses/by/4.0/>
